# Identification of the *PmWEEP* locus controlling weeping traits in *Prunus mume* through an integrated genome-wide association study and quantitative trait locus mapping

**DOI:** 10.1038/s41438-021-00573-4

**Published:** 2021-06-01

**Authors:** Xiaokang Zhuo, Tangchun Zheng, Suzhen Li, Zhiyong Zhang, Man Zhang, Yichi Zhang, Sagheer Ahmad, Lidan Sun, Jia Wang, Tangren Cheng, Qixiang Zhang

**Affiliations:** 1 Beijing Advanced Innovation Center for Tree Breeding by Molecular Design, Beijing Forestry University, 100083 Beijing, China; 2 Beijing Key Laboratory of Ornamental Plants Germplasm Innovation & Molecular Breeding, National Engineering Research Center for Floriculture, Beijing Laboratory of Urban and Rural Ecological Environment, Engineering Research Center of Landscape Environment of Ministry of Education, Key Laboratory of Genetics and Breeding in Forest Trees and Ornamental Plants of Ministry of Education, School of Landscape Architecture, Beijing Forestry University, 100083 Beijing, China

**Keywords:** Genetic markers, Genome-wide association studies

## Abstract

Weeping *Prunus mume* (mei) has long been cultivated in East Asia for its specific ornamental value. However, little is known about the regulatory mechanism of the weeping trait in mei, which limits molecular breeding for the improvement of weeping-type cultivars. Here, we quantified the weeping trait in mei using nested phenotyping of 214 accessions and 342 F_1_ hybrids. Two major associated loci were identified from the genome-wide association study (GWAS), which was conducted using 3,014,409 single nucleotide polymorphisms (SNPs) derived from resequencing, and 8 QTLs and 55 epistatic loci were identified from QTL mapping using 7,545 specific lengths amplified fragment (SLAF) markers. Notably, an overlapping *PmWEEP* major QTL was fine mapped within a 0.29 Mb region on chromosome 7 (Pa7), and a core SNP locus closely associated with the weeping trait was screened and validated. Furthermore, a total of 22 genes in the *PmWEEP* QTL region were expressed in weeping or upright mei based on RNA-seq analysis. Among them, only a novel gene (*Pm024213*) containing a thioredoxin (Trx) domain was found to be close to the core SNP and specifically expressed in buds and branches of weeping mei. Co-expression analysis of *Pm024213* showed that most of the related genes were involved in auxin and lignin biosynthesis. These findings provide insights into the regulatory mechanism of the weeping trait and effective molecular markers for molecular-assisted breeding in *Prunus mume*.

## Introduction

In the plant kingdom, different plant architectures provide each plant with a special ability to adapt to complex environments and compete for light and nutrients. In agriculture, plant architecture is important to crop or orchard growth, management, productivity, and landscaping^1^. This trait is mainly determined by genetic factors and environmental conditions. In the past decade, the development of genomic sequencing technologies has greatly enabled genetic studies of the regulatory mechanism of plant architecture, particularly woody tree architecture^2,3^. To date, several candidate genes associated with controlling tree architecture have been identified^4–7^.

The weeping trait is a special type of tree architecture that is popular in numerous fruit trees, such as peach, apple, cherry, and mulberry^8^. Compared with standard trees, weeping trees generally show downward growth, a shorter height, and a larger branch orientation. Over the last few decades, the weeping trait has been a major focus due to its ornamental value and potential agricultural application^9–11^. However, genetic studies of weeping traits face numerous challenges due to their variable heredity in woody plants. Weeping traits were shown to be controlled by recessive loci in mei (*Prunus mume*)^12^, dominant loci in apple (*Malus domestica*)^10^, both recessive and dominant loci in chestnut (*Castanea crenata*)^13^, and interactions between recessive and epistatic loci in peach (*Prunus persica*)^9^. Recently, *Ppa013325* containing a sterile alpha motif (SAM) domain was found to be associated with the weeping trait in peach, but silencing this gene in plum led to aberrant outward growth that was less consistent with the weeping trait^4^. *LfiGRAS1*, which is involved in GA signaling, can significantly influence the weeping trait in crape myrtle^14^. Thus, the genetic and regulatory mechanisms of weeping traits are quite diverse and complex in woody plants. To date, few genes related to weeping traits have been identified in perennial woody plants because of their complex inheritance patterns, self-incompatibility, and long generation times.

Mei is a woody plant that has high ornamental and economic value and is widely distributed and used in landscape architecture in Asia. Mei has acquired favorable ornamental characteristics, including colorful corollas, pleasant fragrances, and various types of flowers and architecture^15^. The long history of domestication and the distant interspecies or intraspecies hybridization in mei provides a unique set of diverse germplasm collections to study the genetic architecture underlying its complex traits^16,17^. More recently, whole-genome resequencing of 351 accessions and a high-density genetic map of weeping mei have provided a foundation for the identification of weeping-related genes by integrated genome-wide association study (GWAS), quantitative trait locus (QTL) mapping, and selective sweep^12,18^.

In this study, we integrated GWAS and QTL mapping strategies to identify QTLs controlling the weeping trait in mei. An overlapping major QTL marker locus (*PmWEEP*) was identified and validated in representative landraces. Subsequently, a unique candidate gene, *Pm024213* (*PmTrx*), was screened by RNA-seq and validated by qRT-PCR. This study explores the genetic architecture of weeping traits in mei and provides a worthy reference for the exploration of weeping traits in other woody plants.

## Materials and methods

### Plant materials

A population was established by crossing ‘Liuban’ mei (upright type) as the female parent with ‘Fentai Chuizhi’ mei (weeping type) as the male parent. We used a full-sib family of 342 hybrid individuals for the QTL analysis. The F_1_ progenies were grown in 2012 in Deqing County, Zhejiang Province, China (30.53°N, 119.97°E) with a randomized complete block design. We selected another 214 representative landraces of mei to perform a GWAS. These landraces were collected and conserved in Wuhan Mei Garden, Hubei Province, China (30.52°N, 114.31°E).

Genomic DNA was isolated from fresh leaves using the cetyl trimethyl ammonium bromide (CTAB) method^19^. Genomic libraries were prepared according to the manufacturer’s instructions (Illumina). The buds and branches of weeping and upright mei were collected to validate the gene expression level using qRT-PCR. Samples for the tissue-specific expression analysis were collected from 5 to 10 cm grafting shoots and young leaves. The shoots were divided into six parts (Fig. S1). Samples from each individual plant were collected with three biological repetitions and stored at −80 °C until use.

### Assessment and quantitative analysis of weeping traits

The F_1_ segregating population exhibited diverse phenotypic variation in tree architecture, which was classified into upright, intermediate and weeping types (Fig. S2). The nested phenotyping method was used to quantify their characteristics^20^. Secondary branches with a length of 1.0–1.50 m from adult trees before leaf flushing were sampled for measuring the curved angles (T1–T5) and A2 branch angle in February. The A1 branch angle was measured from young branches 20–30 cm in length in April. Branches growing in four different directions on each tree were recorded using a digital camera (D90, Nikon Corporation, Japan), and at least three branches were selected per direction per sample. The branches were divided into five sections (defined as T1–T5), and the curved angles in different sections were extracted using ImageJ software (Fig. S3). The branch angles at the growth stage (defined as A1) and dormancy stage (defined as A2) were also measured as weeping-related traits (Fig. S3). Statistical analysis of the phenotypes was performed using R 3.4.4 software (https://cran.r-project.org/src/base/R-3), and the mean value of each individual was used to perform QTL mapping and GWAS.

### Hormone treatments and histological analysis

Healthy grafted weeping trees were subjected to hormone treatments (0.1% 3-indoleacetic acid (IAA) and 0.1% gibberellic acid (GA) dispersed in lanolin) on the abaxial sides of the young branches. After 24 h, we observed the growth orientation of tender shoots. Furthermore, histological analysis of the young branches of weeping mei and upright mei was performed according to the methods described by Guo et al. ^21^.

### Genotyping of the hybrid and landrace populations

The reference genome for specific length amplified fragment sequencing (SLAF-seq) and resequencing was obtained from the *P. mume* genome project (http://prunusmumegenome.bjfu.edu.cn/). The SLAF-seq strategy was used to determine pedigree genotypes and construct a genetic map as described in our previous study^12^. Finally, 7,545 markers derived from three segregating types (〈hk × hk〉, 〈lm × ll〉, and 〈nn × np〉) were used for QTL analysis. The coding criteria of the markers were described in the QTL IciMapping user’s manual^22^. The marker coding files are shown in Data S1.

The resequencing data of mei were downloaded from the NCBI database (accession number: RP093801; Bio-Project: PRJNA352648). Data preprocessing and SNP calling and filtering were performed by the methods described in our previous study^18^. A total of 3,014,409 SNPs were used for association analysis with the following parameters: a minor allele frequency (MAF) > 0.05, missing data value < 0.1 and linkage disequilibrium (LD) values (correlation coefficient, *r*^2^) < 0.2 and up to ~50 kb. The density of SNPs on each chromosome was evenly distributed (Fig. S4).

### QTL mapping and epistasis analysis

We used the inclusive composite interval mapping (ICIM) method with IciMapping v4.0 software to perform QTL mapping and epistasis analysis^22^. QTL mapping analysis and epistasis mapping were performed using the ICIM-ADD mapping method and ICIM-EPI mapping method, respectively. The parameters were set to the defaults. The epistatic loci explaining <3% of the phenotypic variation were ignored^23^. The relationships between epistatic loci were plotted using Cytoscape 3.7.2 software (https://cytoscape.org/).

### GWAS

We estimated population stratification using ADMIXTURE 1.3^24^ and EIG-6.1.4 (https://data.broadinstitute.org/alkesgroup/EIGENSOFT/). According to the cross-validation error and scree plot, we selected a population number (*k*) = 14 (the cross-validation error was minimal) and the first 10 principal component scores (PCs; the variance was minimal) as the optimum values for the population structure and principal components, respectively (Fig. S5). We used the corresponding population structure matrix (*Q*) and PCs for subsequent GWAS. A kinship matrix (*K*) was calculated using SPAGeDi version 1.4b software^25^. The association analysis was performed using a multinomial logistic model (MLM) with TASSEL v5.0 software^26^. Both the *Q* + *K* and PCs + *K* models were used to correct the population structure and kinship. We adjusted the *P*-value with the Bonferroni correction method and considered *P* ≤ 1.7 × 10^−8^ as significant for SNPs. Manhattan and quantile-quantile (*Q*–*Q*) plots were constructed using the ‘CMplot’ R package, and LD was assessed using PLINK 1.9^27^ and Haploview software^28^.

### Genome-wide selective sweep analysis

We further identified the footprints of positive selection in weeping mei. Using VCFtools v.0.1.15^29^, we calculated the genome-wide nucleotide diversity (*Pi*), allele frequency (Freq), and genetic differentiation (*F*_ST_) between the weeping subpopulation and upright subpopulation for each SNP and estimated the mean statistics for each 10-kb nonoverlapping window across the genome. Distribution patterns and Manhattan plots of *F*_ST_ and *Pi* were subsequently generated using the ‘CMplot’ R package.

### Validation of significant SNPs through genotyping

A Sequenom MassARRAY analysis was performed to validate the significant GWAS-derived SNPs located within the major QTL intervals in the hybrid population (289 individuals) and the landrace population (69 accessions) using a MassARRAY compact system (Sequenom, San Diego, CA). Moreover, QTL-derived markers were validated in a landrace population (99 accessions) using the same methods. Primers for these markers (flanking the SNP site for PCR amplification and extension) were designed for genotyping. The primers and genomic sequences are listed in Table S1 and Appendix S1, respectively. PCR was performed in 384-well plates using HotStart *Taq* DNA polymerase (Qiagen). The steps of the PCR program and application of shrimp alkaline phosphatase (SAP) were conducted in accordance with the protocols in the Sequenom iPLEX Application Guide (Version 1, Sequenom, San Diego, CA). Primer extension was performed using an iPLEX^TM^ Reagent Kit (Sequenom), and the genotype was detected using a matrix-assisted laser desorption/ionization-time-of-flight (MALDI-TOF) mass spectrometer for SNP genotyping.

### RNA preparation, RNA-seq, and transcriptome analysis

RNA-seq was conducted using a bulked segregant analysis (BSA) strategy^30^. Tissues were collected from the buds and young branches of F_1_ seedlings with contrasting phenotypes when dormant buds sprouted out to 3–5 cm. We divided the samples into four groups (weeping vs. upright and branches vs. buds), and 20 genotypes were pooled, with three biological replicates per group. Total RNA was extracted from each sample using EasySpin RNA Reagent (Aidlab Biotechnologies, China) according to the manufacturer’s instructions. The RNA concentration and integrity were assessed using an Agilent 2100 system. Libraries were generated using a NEBNext Ultra^TM^ RNA Library Prep Kit for Illumina. The amplified fragments were sequenced using an Illumina HiSeq X-10 platform. All raw data were stored in the Genome Sequence Archive (GSA) under project ID CRA001273.

Clean reads were obtained by analyzing their Q20 and Q30 values and their GC content. High-quality sequences were mapped to the *P. mume* reference genome and normalized into fragments per kilobase of transcript per million mapped reads (FPKM) values. Differentially expressed genes (DEGs) were analyzed using the DESeq R package. The Benjamini–Hochberg procedure was used to correct the *P*-values to maintain a false discovery rate (FDR) < 0.001. Genes with an adjusted *P*-value < 0.05 and an absolute fold change (FC) ≥ 1.5 (|log_2_Ratio| ≥ 0.585) were defined as DEGs. Functional annotation of all DEGs was performed using the Gene Ontology (GO) and Kyoto Encyclopedia of Genes and Genomes (KEGG) databases. MapMan software (http://mapman.gabipd.org/) was used to identify DEG categories using TAIR version 10 (http://www.arabidopsis.org/) as a reference, with a *P*-value < 0.05 for ontology. An interactive network of coexpressed genes was constructed using the String database^31^. The transmembrane domains and subcellular localization were predicted using TMHMM server v.2.0 (http://www.cbs.dtu.dk/services/TMHMM/) and Plant-mPLoc (http://www.csbio.sjtu.edu.cn/bioinf/plant-multi/) online tools, respectively.

### Validation of DEGs using qRT-PCR

Total RNA was extracted using the same method described for RNA-seq. First-strand cDNA templates were synthesized from ~2 μg of total RNA using a PrimeScript^TM^ RT Reagent Kit (TaKaRa, Beijing, China). Specific primers were designed using an online tool (http://sg.idtdna.com/scitools/Applications/RealTimePCR/) and checked using the Primer-BLAST tool on NCBI (https://www.ncbi.nlm.nih.gov/tools/primer-blast/). The *protein phosphatase 2A* (*PP2A*; *Pm006362*) gene was used as an internal reference^32^. Detailed information about the primers is presented in Table S2. The programs were conducted using a CFX96 Real-Time PCR Detection System (Bio-Rad, Hercules, CA, USA) with SYBR Premix Ex*Taq* II (TaKaRa). Relative expression levels were calculated using the delta–delta CT method^33^. Each sample was analyzed in three biological repeats.

## Results

### Correlation analysis of the weeping and upright phenotypes

The variation in tree architecture across the segregating population indicated that the weeping trait in mei is controlled by multiple genes (Fig. S2). We measured seven weeping-related subtraits in the F_1_ population (342 progenies) and GWAS panel (214 accessions) using the nested phenotyping method to overcome the limitation of a low phenotype resolution and improve the ability to identify potentially major and minor genes. Based on the results of the phenotypic variation analysis, the coefficient of variation increased from the T1 to T5 subtraits, but the branch angle varied less in the dormant stage than in the growth stage (Table S3). The phenotype analysis revealed strong correlations between the seven subtraits (Pearson’s correlation coefficient *r* > 0.59) in the F_1_ population and GWAS panel (Fig. S6), suggesting that these subtraits potentially represent the characteristics of weeping traits. The frequency distributions of the seven subtraits showed two peak in the F_1_ populations and approximately normal distributions in the GWAS panel (Fig. S7).

### Detection of an overlapping QTL via GWAS, selective sweep mapping and QTL mapping

#### GWAS

**Fig. 1:**
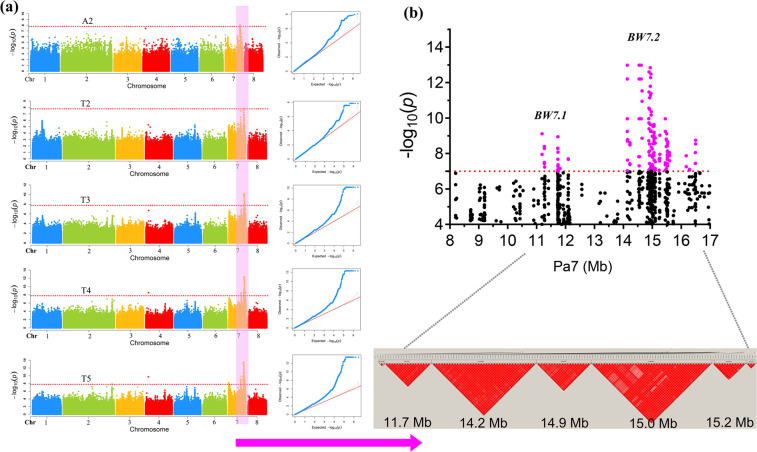
GWAS of the weeping trait in *P. mume*. **a** Manhattan plots and *Q*–*Q* plots for five significantly associated subtraits. **b** Pairwise LD blocks (*r*^2^ > 0.9) of SNPs (*P*-value < 1 × 10^−7^) located in the *BW7.1* and *BW7.2* loci

Subpopulation structures may confound GWAS, resulting in a reduced mapping resolution and an increased error of false positives^34^. We used the *Q* + *K* and PCs + *K* models to correct the subpopulation structure. When expected values and observed values were analyzed based on the *Q*–*Q* plots, the *Q* + *K* model provided more satisfactory results than the PCs + K model (Fig. S8). Significant SNPs (*P* < 1.7 × 10^−8^) on Pa7 associated with five subtraits, except for the A1 and T1 subtraits, were identified (Fig. 1a). Significant SNPs clustered in the 11.1–11.8 and 14.1–15.5 Mb regions were defined as *BRANCH AND WEEPING ON CHROMOSOME 7.1* (*BW7.1*) and *BW7.2*, respectively (Fig. 1b). Strong LD (*r*^2^ > 0.9) was detected between the *BW7.1* and *BW7.2* regions (Fig. 1b). Among these significant SNPs, 125 SNPs were repeatedly associated with more than two subtraits (Table S4).

#### Genome-wide selective sweep mapping

**Fig. 2:**
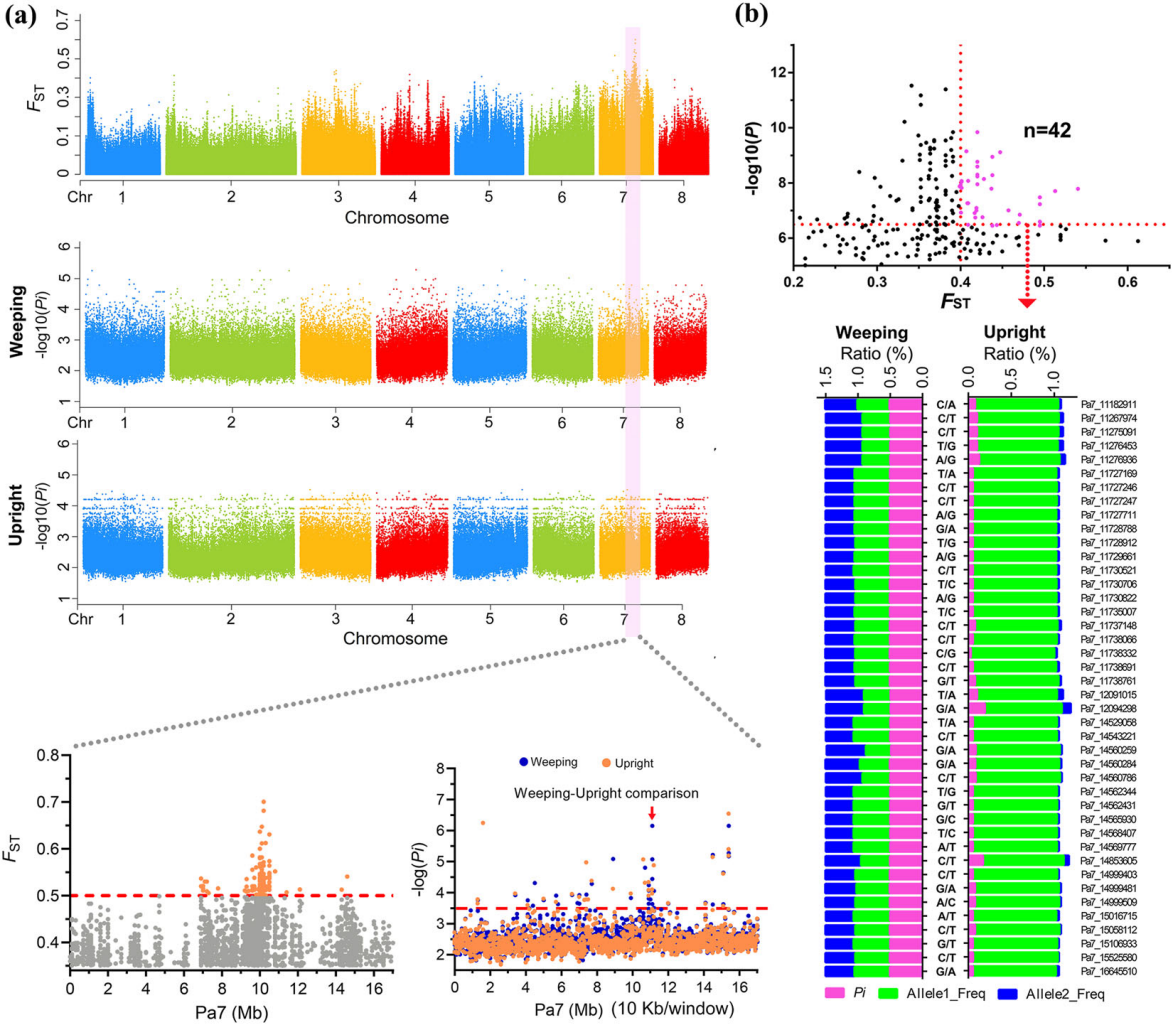
Analyses of the signatures of genome selection between weeping and upright mei. **a** Genomic landscape of the *F*_ST_ values and *Pi* values in the genomes of the weeping subpopulation and upright subpopulation. **b** Significant alleles were selected through the combination of the *F*_ST_ value and GWAS *P*-value. SNPs with higher GWAS *P*-values and *F*_ST_ values also presented substantial differences in the *Pi* value and allele frequency between weeping and upright trees

Significant footprints were identified at approximately 9.21–10.83 Mb (*F*_ST_ ≥ 0.5) and 10.74–12.18 Mb (−log(*Pi*) ≥ 3.5) on Pa7 by calculating *F*_ST_ and *Pi* (Fig. 2a, Data S2). The top position of *F*_ST_ and *Pi* between weeping and upright groups was located at 10.25 and 11.1 Mb, respectively. We further compared the difference in variant number between weeping and upright mei with −log(*Pi*) ≥ 3.5, and five specific regions in weeping trees appeared at 10.91–11.18 Mb (Table S5), which overlapped with the *BW7.1* region. This result indicates that the crucial regions controlling weeping traits were probably ~11 Mb on Pa7. To further screen reliable SNPs, 42 SNPs were selected with higher GWAS *P*-values and *F*_ST_ values (Fig. 2b); these are presented in Table S6. Their *Pi* value and allele frequency also showed great differences between weeping and upright trees (Fig. 2b).

#### QTL mapping

**Fig. 3:**
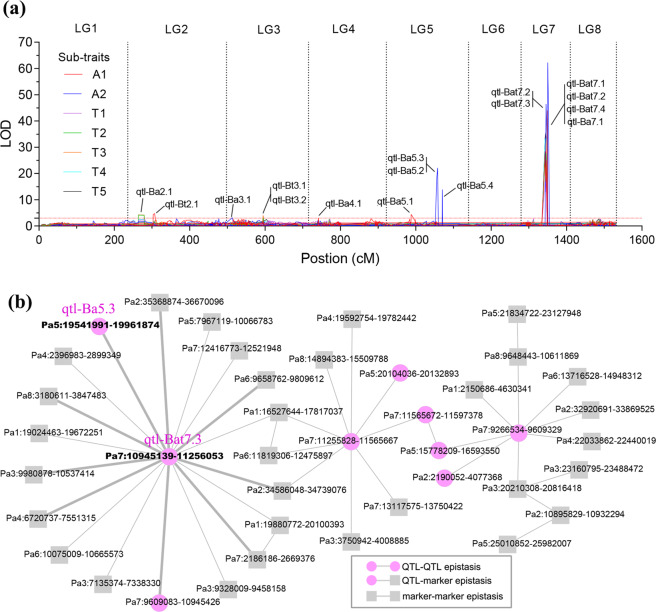
Identification of QTL peaks for seven subtraits of the weeping trait using inclusive composite interval mapping. **a** Distribution of major and minor effects on linkage groups. The dashed line indicates a LOD value > 3. **b** Analysis of epistatic interactions. The bold gray line indicates epistatic loci that were repeatedly detected for more than two subtraits

QTL mapping was performed using 342 five-year-old F_1_ hybrids with 7,545 SLAF markers to further evaluate the genetic architecture of weeping traits in mei. Sixteen significant QTL peaks (logarithm of odds (LOD) threshold values > 3, *r*^2^ > 1.0%) were identified on five linkage groups (LGs), which explained 1.2–55.3% of phenotypic variation (Fig. 3a and Table S7). Among them, nine peaks were uniquely detected by branch angles (A1 and A2 subtraits), and two peaks were simultaneously detected by six subtraits on chromosome 7. Only the qtl-Bat7.4 (Pa7:11255828–11565667) peaks were detected by all seven subtraits (Table S7). Thus, these subtraits were significantly associated with the weeping trait. Combining QTL peaks that were close to each other, one major QTL and seven minor QTLs were determined (Table S7). In addition, we identified 55 epistatic pairs for seven subtraits (explaining 3–31.6% of the phenotypic variation) and determined their interactions (Fig. 3b and Table S8). The Pa7:10945139–11256053 region was a key epistatic locus overlapping with qtl-Bat7.3 and *BW7.1* (Fig. 3a), which was cross-validated by both QTL mapping and GWAS. Furthermore, this region also presented strong epistasis with qtl-Ba5.3 (specifically mapped by branch angles) (Fig. 3b), thereby indicating the existence of an epistatic interaction that controls the weeping trait in mei.

### Validation of the overlapping major QTL through marker genotyping

**Fig. 4:**
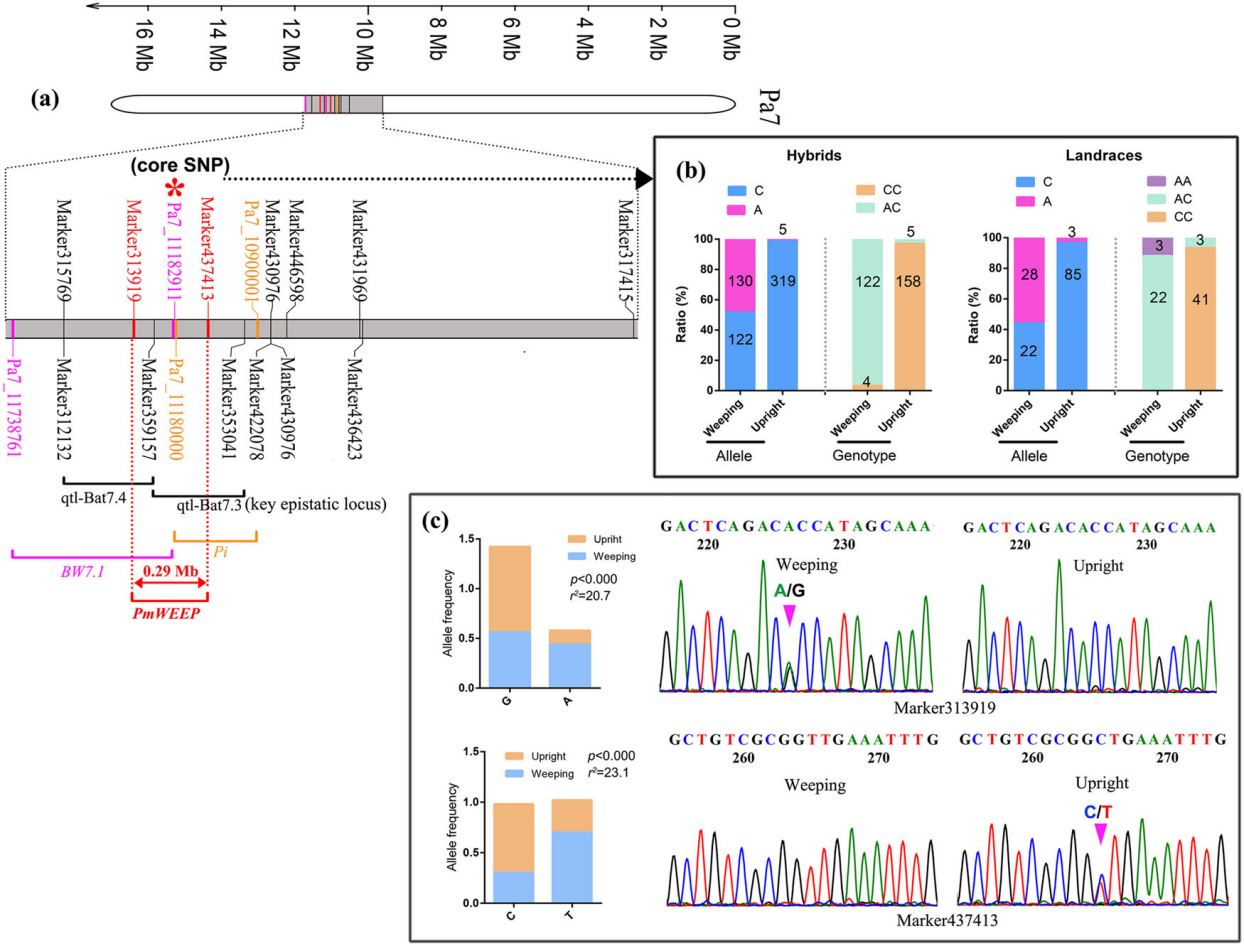
Identification and validation of the *PmWEEP* major QTL interval. **a** Distribution of closely linked markers derived from GWAS and QTL mapping. An overlapping *PmWEEP* region was identified. **b** Validation of significant GWAS-derived markers and **c** QTL mapping-derived markers located in the *PmWEEP* region. GWAS-derived markers were validated in a biparental population (289) and landraces (69); QTL mapping-derived markers were validated in landraces (129)

The integration of GWAS (*BW7.1* locus 11.1–11.8 Mb), selective sweep mapping (10.90–11.18 Mb), QTL mapping (qtl-Bat7.3 (10.94–11.25 Mb) and qtl-Bat7.4 (11.25–11.56 Mb)), and epistatic analysis (10.94–11.25 Mb) allowed us to identify an overlapping major QTL located at 11.1–11.25 Mb on Pa7 (Fig. 4a). Moreover, we selected 19 SNPs derived from GWAS across significantly associated loci and eight closely linked SLAF markers derived from QTL mapping to further validate the overlapping QTL region in a new population using a genotyping strategy (Appendix S1 and Data S3). Most of these markers were closely linked to the weeping trait (Figs. S9 and S10). The most significant GWAS-derived SNP was Pa7_11182911 (A/C allele); 158 of 163 upright hybrids (96.9%) and 41 of 44 upright landraces (93.2%) exhibited the C/C homozygous allele, while 144 (122 hybrids + 22 accessions) of 151 weeping trees (95.4%) exhibited the A/C heterozygous allele (Fig. 4b). Thus, Pa7_11182911 was defined as one of the core SNPs associated with the weeping trait in mei. The genotyping of SLAF-derived markers also showed that the top two significant markers, Marker437413 (Pa7:11037771) and Marker313919 (Pa7:11324965), were located quite close to the core SNP (Fig. 4a, c). The overlapping locus was extended to avoid the potential loss of candidates and finally determined to be located at the 11.03–11.32 Mb region (defined as *PmWEEP*) on Pa7 (Fig. 4a).

### Identification of a key gene in the *PmWEEP* region using RNA-seq and qRT-PCR

**Fig. 5:**
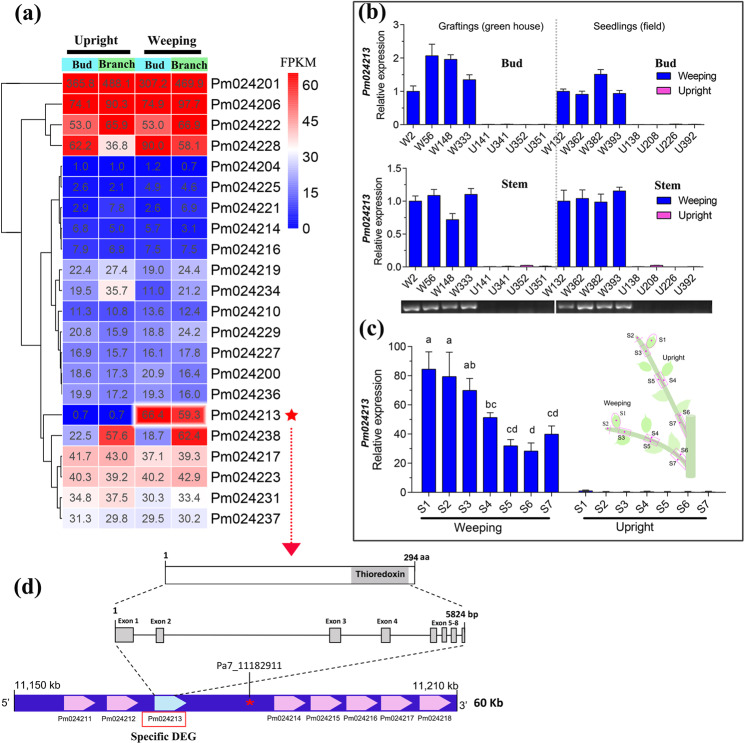
Analysis of the expression patterns of candidate genes. **a** 22 of 39 candidate genes located in the *PmWEEP* region were expressed in the buds or branches of mei. The red star represents the specifically expressed gene. **b** Validation of the specifically expressed gene (*Pm024213*) using qRT-PCR. *W* indicates weeping individuals; *U* indicates upright individuals. Graftings indicate 1-year-old grafted plants grown in the greenhouse (Beijing); seedlings indicate 5-year-old seedling plants grown in the field (Zhejiang). **c** Analysis of tissue-specific expression of *Pm024213* in seven tissues from grafting shoots. S1–S7 indicate different tissues of shoots of weeping and upright plants represented in the schematic diagram (right). The different lowercase letters represent significant differences at the 0.01 level. **d** Gene structure and protein domains of *Pm024213*. Gray boxes and black lines indicate exons and introns, respectively

Thirty-nine candidate genes were identified within the *PmWEEP* region. The functional annotations and positions of these genes are shown in Data S4. Gene expression profiling using RNA-Seq showed that 22 of 39 candidate genes were expressed in buds or branches in at least one of the pools (FPKM ≥ 1.0) (Data S4). Among the expressed genes, only three upregulated DEGs (*Pm024213*, *Pm024225* and *Pm024228*) and two downregulated DEGs (*Pm024214* and *Pm024234*) were identified in weeping mei (Data S4 and Fig. 5a). Notably, *Pm024213* (Pa7:11168079–11173903), which was very close to the core SNP (Pa7:11182911), presented a striking upregulation in weeping pools; its average FPKM values were 66.4 and 59.2 in buds and branches, respectively. However, the FPKM values of both branches and buds were less than 1.0 in upright mei (Fig. 5a).

qRT-PCR was performed using hybrids from 1-year-old grafted trees (grown in a greenhouse) and 5-year-old seedling trees (grown in the field) with contrasting phenotypes to further confirm the expression of *Pm024213*. The results showed that *Pm024213* was specifically expressed in weeping trees, and its expression pattern was not affected by the environment or rootstocks (Fig. 5b). Additionally, tissue-specific expression analysis further revealed higher expression levels of *Pm024213* in young tissues than in old tissues, and tissue-specific expression occurred in all sections of weeping shoots (Fig. 5c).

### Functional and structural characteristics of *Pm024213*

The gene structure of *Pm024213* revealed eight exons and seven introns (Fig. 5d). The functional annotation indicated that *Pm024213* was a novel gene with unique roles. Domain searching of Pm024213 revealed a significant match with a thioredoxin (Trx) domain in *M. domestica* (Appendix S2). The Trx protein family is a class of redox proteins present in all organisms. In plants, Trx proteins are mainly involved in thylakoid energy transduction, gene expression, metabolism and growth and development through the light-mediated activation of many key enzymes in the chloroplast, cytoplasm, and mitochondria and the reversible formation of disulfide/dithiol^35,36^. Bioinformatics analysis of the transmembrane domains and subcellular localization of Pm024213 showed that Pm024213 was predicted to be located in chloroplasts and contain multiple transmembrane domains (Fig. S11). The neighbor joining (NJ) tree of 13 species was constructed using MEGA 6.0 software^37^. The phylogenetic relationships showed that Pm024213 was closer to *Prunus yedoensis* and *Prunus avium* than to other species (Fig. S12).

### Construction of potential regulatory relationships based on *Pm024213* coexpression analysis

**Fig. 6:**
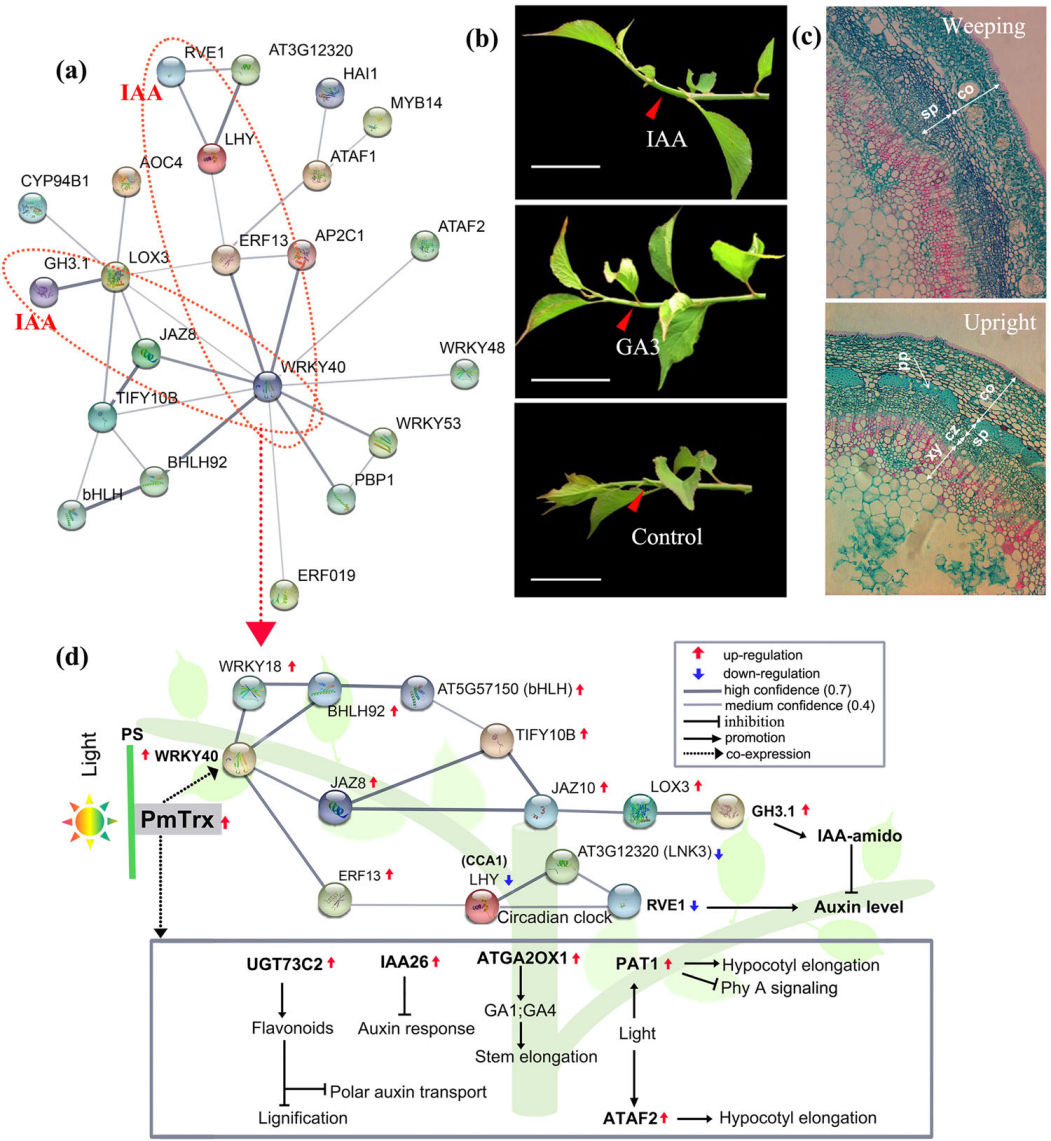
Construction of regulatory networks putatively associated with the control of the weeping trait in *P. mume*. **a** Predicted interaction networks of 35 genes located in epistatic loci. The two red dashed ovals represent pathways associated with IAA regulation. **b** IAA and GA_3_ were applied to the abaxial surfaces of the elongating stem of weeping shoots. **c** Histology of young branches of weeping mei and upright mei. pp phloem fibers, xy secondary xylem, cz cambial zone, sp secondary phloem, co cortex. Bars = 200 μm. **d** Hypothetical regulatory networks for weeping the trait, including auxin regulation, hypocotyl elongation, and lignin biosynthesis cues. The annotations of these genes are listed in Table 1

Based on the results of the RNA-seq analysis, we further detected genes that were coexpressed with *Pm024213* (*PmTrx*) using *k*-means clustering^38^. Eighty-five genes probably had a relationship with *Pm024213* (Fig. S13a and Table S9). Most of these genes are associated with MapMan categories of transcriptional regulation and hormones (Fig. S14). The epistatic analysis showed that the *PmWEEP* QTL strongly interacted with multiple loci (Fig. 3b). We further investigated the genomic position of these 85 genes. Thirty-five genes were found at or near the epistatic loci (Fig. S13b and Table S9), and 22 of these 35 genes exhibited strong interactions according to the STRING database^31^. Two regulatory pathways associated with auxin levels were identified in the interaction network (Fig. 6a and d).

GA application has been shown to rescue the weeping phenotype^39,40^, and asymmetric auxin transport also affects asymmetric organ growth^41,42^. Both GA and IAA were applied to the abaxial surfaces of the young branches in weeping mei to investigate their role in the weeping trait (Fig. 6b). IAA treatments significantly altered the branch growth trajectory compared to GA_3_ treatments, which caused moderate upward growth. In our previous study, the IAA concentration in young branches was significantly higher in upright mei than in weeping mei^43^. In addition, several coexpressed genes were also involved in lignification, the auxin response and hypocotyl elongation (Fig. 6d). Histochemical staining results revealed the aberrant development of phloem and cortex cells, loss of phloem fibers, irregular arrangement of cells and slow development of secondary xylem in the young branches of weeping trees (Fig. 6c). The above results further support that the coexpressed genes were related to auxin levels, stem elongation, and lignin biosynthesis in the network. Notably, most of these genes were located at epistatic interaction loci and reported to be associated with stem development (Table 1), suggesting that these genes are probably related to *Pm024213* (Table 1). Based on the regulatory network and reported studies, we propose a hypothetical regulatory model of the weeping trait that is associated with auxin levels, stem elongation, and lignin biosynthesis (Fig. 6d).

**Table 1:** Coexpressed genes that are potentially involved in controlling weeping traits in the hypothetical regulatory network

Gene ID	Gene name	Annotated function	References	Bud		
				log2(FC)^a^	qRT-PCR^b^	Regulation
*Pm012630*	*ATAF2*^c^	No apical meristem (NAM) protein: Auxin biosynthesis; hypocotyl growth	^72,73^	2.70	8.18	Up
*Pm021243*	*GH3.1*^c^	IAA-amido synthases: Hypocotyl and root length	^61,62^	2.91	6.96	Up
*Pm011163*	*ATGA2OX1*^d^	Gibberellin 2-beta-hydroxylase 1: Stem elongation	^67^	3.27	3.02	Up
*Pm023083*	*UGT73C2*^d^	UDP-glycosyltransferase: Cell wall lignification; polar auxin transport	^70,74^	2.87	2.38	Up
*Pm013791*	*WRKY40*^d^	WRKY transcription factor 40: Indolic secondary metabolites; light-harvesting	^75^	4.15	2.21	Up
*Pm029452*	*PAT*1	Chitin-inducible gibberellin-responsive protein 1: Response to far-red light; hypocotyl elongation	^69^	2.02	3.37	Up
*Pm005182*	*IAA26*^c^	Phytochrome-associated protein 1: Repressors of auxin response genes	^66^	1.94	2.51	Up
*Pm012998*	*RVE1*^c^	REVEILLE 1: The circadian clock and auxin pathways	^63^	−3.26	0.21	Down
*Pm028731*	*LHY*	LATE ELONGATED HYPOCOTYL: Circadian rhythm	^76^	−5.60	0.43	Down
*Pm024213*	*PmTrx*^d^	Putative thioredoxin protein: Light activation of enzymes	^36^	6.89	79.34	Up

^a^Indicates the fold-change of differentially expressed genes (DEGs) from RNA-seq

^b^Indicates the relative expression levels of DEGs validated by qRT-PCR

^c^Represents candidate genes located in epistatic interaction loci

^d^Represents epistatic interaction loci detected by more than two subtraits

## Discussion

### Resolution of the weeping phenotype substantially contributes to the detection of QTLs

Although tree architecture plays pivotal roles in esthetic beauty and the management of orchard crops, the understanding of the genetic architecture underlying shoot development remains limited. Several studies have been performed to explore the genetic architecture of weeping, pillar and columnar tree architectures^12,18,44,45^. However, few minor-effect QTLs have been detected due to single discrete phenotypic categorization, a low density of markers, or a small population. Due to the effect of low-resolution phenotypes, previous studies just detected a large major QTL associated with the weeping trait on chromosome 7 in mei^12^. However, the progenies with various intermediate characteristics implied the existence of minor-effect QTLs in mei (Fig. S2). In peach, *Ppa013325* was found to be a regulator of the weeping trait, but RNAi-mediated silencing of a plum homolog did not completely result in weeping characteristics^4^. In crape myrtle, silencing the GA-related *LfiGRAS1* gene also did not completely alter weeping growth habits^14^. Therefore, we inferred that minor-effect QTLs also play an essential role in weeping traits.

In the present study, the quantification of the weeping trait with the nested method successfully detected major-effect and minor-effect QTLs and epistasis effect loci (Figs. 1 and 3). Notably, an overlapping major QTL (*PmWEEP*) was detected in different populations with cross-validation methods. The overlapping QTL was further validated in a new population via a genotyping approach (Fig. 4). Seven small effect loci and 55 epistatic loci related to the control of weeping traits were first detected in mei. These results were attributed to the combination of the high-resolution phenotype, high-density genetic map from SLAF-seq, high-quality SNPs from resequencing, and large populations. The subdivision of the weeping trait into multiple measurable subtraits might minimize the variance and maximize the ability to detect differences between subtraits in GWAS and QTL mapping studies, which has been widely used in rice for panicle traits^46^. Significant QTLs or associated SNPs are repeatedly detected by multiple subtraits using nested phenotyping, which increases the reliability of QTL mapping results. In our study, the *PmWEEP* locus was detected by multiple subtraits in both QTL mapping and GWAS.

Moreover, qtl-Ba5.3 located on chromosome 5 was specifically detected based on branch angles and exhibited significant epistatic interactions with qtl-Bat7.3 on Pa7 (Fig. 3). The QTL–QTL epistatic interaction provides evidence for the presence of a genetic interaction between branch angle and weeping growth habit. This supports the hypothesis of a genetic interaction between pillar and weeping traits, leading to the “archer” phenotype in peach^9^. Therefore, the novel phenotyping of the weeping trait and high-resolution phenotypes substantially contribute to the identification of alleles controlling the weeping trait in mei.

### *Pm024213* located in the PmWEEP locus putatively controls the weeping trait

The GWAS results showed that the *BW7.1* and *BW7.2* loci were strongly associated with the weeping phenotype. However, only the *BW7.1* locus overlapped with qtl-Bat7.3 (also a key epistatic locus), qtl-Bat7.4 and selective sweep loci (Fig. 4a). Additionally, the top significant markers validated in a new population were all located at or near the *BW7.1* locus (Fig. 4). This evidence indicates that the *BW7.1* locus is putatively responsible for controlling the weeping trait. Mei has a long domestication history in China, and weeping cultivars may be selected for their ornamental appeal. Although significant SNPs derived from the *BW7.2* locus were also closely linked to the weeping trait in mei (Figs. 1b and S9), we hypothesized that the significant *BW7.2* locus may be associated with the subpopulation structure and extensive LD caused by human selection. A similar phenomenon has also been observed in other species by GWAS^34,47^. Based on the results described above, the overlapping *BW7.1* region (*PmWEEP*) was finally determined to be located at 11.03–11.32 Mb on chromosome 7.

Twenty-two candidates were expressed in the *PmWEEP* region. However, only *Pm024213* presented significant upregulation and specific expression in weeping trees based on RNA-seq analysis (Fig. 5a). The relative expression level and tissue-specific expression patterns further validated this result (Fig. 5b and c). Moreover, *Pm024213* is quite close to the core SNP Pa7_11182911 (within 9 kb). Therefore, we suggest that *Pm024213* is the most promising candidate gene responsible for the weeping trait in mei.

*Pm024213* is predicted to be an uncharacterized membrane protein located in chloroplasts and contains a Trx domain (Appendix S2). The Trx protein family is present in a wide range of prokaryotic and eukaryotic organisms with unprecedented versatility in plants^36^. The structures and functions of Trxs are differ across plant species. One of the functions involves the light-induced activation of many key enzymes in the chloroplast, cytoplasm, and mitochondrion via the reversible formation of disulfide/dithiol^35,36^. In poplar, tension wood (TW) formation requires reactive oxygen species (ROS) induced through the accumulation of thioredoxin h (Trx h) proteins^48^. The production of ROS is critical for auxin-induced gravitropic signaling in roots^49^. In addition, the induction of *Trx h* can respond to gravitropic stimuli via amyloplast sedimentation at the base of poplar stems^50^. An in situ immunolocalization assay further showed that Trx h1 colocalized with the amyloplasts of endoderm cells in the stem, which has been suggested to be essential for shoot gravitropism^50,51^. In this study, we found that the development of endoderm cells in the stem was abnormal in weeping mei (Fig. 6c), but IAA treatments prominently recovered the upright growth trajectory of weeping mei (Fig. 6b). In other weeping trees, gravity perception in the endodermis serves as a critical factor in the regulation of shoot growth orientation^4,14^. This evidence implies that *PmTrx* is involved in the auxin-mediated statolith process for stem gravitropism in controlling the weeping trait in mei^52^.

Although there is no clear report of *Trx* transgenic plants with weeping traits, new insights into the role of redox-related proteins in conferring plant architecture in poplar and apple provide some evidence for a role of *Trx* in weeping trait formation. For instance, upregulation of a putative 2OG-Fe(II) oxygenase gene was caused by insertion of a Gypsy-like retrotransposon, resulting in the columnar phenotype of apple^7^. A proteomics study indicated that chloroplast Trxs can promote starch degradation^53^. The sedimentation of starch-filled amyloplasts could trigger gravity signal transduction, resulting in gravity response and gravitropic bending in the elongation zone^54^. *Pm024213* is predicted to be a chloroplast *Trx*. Whether high expression of this gene in weeping mei reduces the sedimentation of amyloplasts and influences gravity signal transduction needs further exploration.

### Functional characteristics of genes coexpressed with *Pm024213*

To explore potential pathways and biological processes in weeping mei, we identified the DEGs coexpressed with *Pm024213*. A total of 85 DEGs were identified. Remarkably, 35 of the 85 genes were located in or near epistatic loci and presented strong interactions via STRING database predictions. In this interaction network, two pathways were associated with IAA regulation (Fig. 6a, d), suggesting the potential relationship among *PmTrx*, IAA, and gravity perception in the regulation of weeping traits.

Auxin is recognized as a crucial regulator of plant growth, development and defense^55^. Several genes in the pathways mentioned above are known to be associated with auxin. *WRKY40* (*Pm013791*), a very significant gene that interacts with multiple genes in these networks, is located at the Pa4:6720737–7551315 locus and presents a strong epistatic interaction with the Pa7:10945139–11256053 (qtl-Bat7.3) locus (Fig. 3b). Members of the WRKY gene family are known to be involved in biotic and abiotic stress responses^56^. *WRKY40* has been reported to be involved in the production of indolic secondary metabolites, balancing the function of light-harvesting chlorophyll a/b-binding proteins and modulating distinct plant hormone pathways^57–59^. *GH3.1* and *REVEILLE 1* (*RVE1*) were downstream IAA-related regulatory genes (Fig. 6b and Table 1). *GH3.1* encodes an IAA-amino synthase, and the constitutive expression of *GH3.1* in rice substantially decreases auxin levels and plant height^60^. In *Arabidopsis*, the *GH3* homologs *DWARF IN LIGHT 1* (*DFL1*) and *YADOKARI1* (*YDK1*) are involved in the light response and hypocotyl and root elongation^61,62^. Upregulation of the *GH3.1* gene in weeping trees may reduce the IAA level and attenuate the light response.

*NIGHT LIGHT–INDUCIBLE AND CLOCK-REGULATED* (*LNK*) and *LATE ELON-GATED HYPOCOTYL* (*LHY*) are the upstream genes of *RVE1*. *LHY* belongs to the MYB transcription factor family and is involved in modulating gene expression, thereby affecting the circadian clock of plant growth and development^63^. These three genes play important roles in IAA homeostasis in plants^63–65^. Interestingly, they were all commonly downregulated in weeping mei (Fig. 6d and Table S9), causing reduced free auxin levels^63^. In addition, *IAA26* (*Pm005182*), a repressor of early auxin response genes^66^, was upregulated in weeping mei (Table 1). Therefore, IAA-related coexpressed genes mainly contribute to reducing IAA levels, affecting auxin-mediated gravity perception or light response processes.

In addition, several coexpressed genes related to gibberellin, hypocotyl elongation, and lignification were also likely to be involved in the regulation of weeping traits. For example, overexpression of *GA2-oxidase* (*Pm011163*) affects stem elongation in poplar^67^. *ATAF2* suppresses cytochrome P450-mediated brassinosteroid inactivation, resulting in elongated hypocotyl phenotypes in *Arabidopsis*^68^. *PAT1* is involved in phytochrome A signal transduction and hypocotyl elongation^69^. *UGT73C2* (*Pm023083*), which belongs to the UDP-glycosyltransferase family involved in monolignol glycosylation and cell wall lignification, was significantly upregulated. The loss of its homologous *GT72B1* potentially results in increased lignification and lignin biosynthesis^70,71^. In this study, we found aberrant development of phloem and cortex cells in the stem of weeping mei (Fig. 6c). Thus, these coexpressed genes involved in the development of xylem or phloem may also influence the weeping trait in mei.

With the above results in mind, a strategy integrating GWAS and QTL mapping is effective to identify the genes regulating the weeping trait. An overlapping *PmWEEP* locus was identified to be responsible for the weeping trait. *Pm024213*, containing a Trx domain, is a key candidate gene in the *PmWEEP* locus. Evidence implies that *PmTrx* is involved in regulating the weeping trait in mei via auxin-mediated gravity perception or light response processes. In addition, a large number of coexpressed genes are also involved in regulating auxin levels, hypocotyl elongation, and lignin biosynthesis. Therefore, our study will be beneficial to understand the genetic architecture of the weeping trait and molecular marker-assisted breeding in mei.

## Supplementary information

Supporting table

Supporting Appendix1

Supporting Appendix2

Supporting Appendix3

Supporting figure

Dataset1

Dataset2

Dataset3

Dataset4

## Data Availability

The raw whole-genome resequencing data involved in this study is available at the NCBI with the accession number SRP093801 (Bio-Project: PRJNA352648). The SLAF-seq raw data are available at the NCBI under project accession number PRJNA273338. The RNA data in this study have been deposited in the National Genomics Data Center (NGDC) with the accession number PRJCA001153 (Bio-Project: CRA001273). All other relevant supporting data are available in the Supplementary files included with this article.
