# Supplementary material for: Identification of the PmWEEP locus controlling weeping traits in Prunus mume through an integrated genome-wide association study and quantitative trait locus mapping
Source: Hortic Res. 2021 Jun 1;8:131. doi: 10.1038/s41438-021-00573-4 (PMC8167129; doi:10.1038/s41438-021-00573-4)
Supplement: Supplementary file 1 — Supporting table [file 41438_2021_573_MOESM1_ESM.pdf]

Table S1 A list of primers used to genotype selected markers with the MassARRAY compact system.

SNP_ID	Source of markers	Chr	Position	Ref.	Alt.	PCR Primer1 (F)	PCR primer2 (R)	Direction of extension primer	Single nucleotide extension primer
Pa7_11182911	GWAS	7	11182911	C	A	ACGTTGGATGGGTGTGTTTCTTTCTAACGAG	ACGTTGGATGTGCTTGTCAAACACAGTCCG	R	ACTAACCTCATTTCATAAGTTGA
Pa7_11727711	GWAS	7	11727711	A	G	ACGTTGGATGGGCAAGAGAAAGCTTCCTAC	ACGTTGGATGTGCATATGCTCCAGAGAGTG	F	agcacGAGAAAGCTTCCTACCTTATT
Pa7_11728912	GWAS	7	11728912	T	G	ACGTTGGATGTGCCTTCGTAGCAAATGCAG	ACGTTGGATGTGAGAACGTATTTGAGGTGC	R	cctgGCATAACTTCTTCTTCAGTTC
Pa7_11729661	GWAS	7	11729661	A	G	ACGTTGGATGAGCTAGCAGCATTGCAGTTC	ACGTTGGATGACGACAATGGAGTCAGCTAC	R	cacGTTCATACAGCACAACAA
Pa7_11866123	GWAS	7	11866123	G	C	ACGTTGGATGATTGCCGGATGGAGATTTGG	ACGTTGGATGCTCGATCGTACTCTTCGATG	R	ACTCTTCGATGATATTAGCTAACTC
Pa7_11918846	GWAS	7	11918846	T	C	ACGTTGGATGGACATATTTTTACATGAACC	ACGTTGGATGCACCAACCTTTCTGAAATAGC	F	CATGAACCATCAAACCAGAT
Pa7_11936850	GWAS	7	11936850	A	G	ACGTTGGATGTTTGACATCCTGTAGACGCC	ACGTTGGATGTTTGGTGCCAGATACTGGTG	F	tACGCCAACCTGCAATCA
Pa7_11969160	GWAS	7	11969160	C	T	ACGTTGGATGGGCCAATGCGAACCCTAATT	ACGTTGGATGTCAAACACACGGACAGGAAC	F	ggcgAACAACGATGCGTAAGACGAT
Pa7_14544359	GWAS	7	14544359	T	A	ACGTTGGATGTGTTGTTGATACCCGTCTTC	ACGTTGGATGTCTTACCAAGGTGGTCATCG	R	TGGGTGGAGCTTGACAT
Pa7_14562431	GWAS	7	14562431	G	T	ACGTTGGATGAATACCCCCTTCCCTCTTTC	ACGTTGGATGTGTCTTGCAAGGAGGAAAAG	F	ccttCCTCTCTCTCCGTAAAAA
Pa7_14903156	GWAS	7	14903156	C	A	ACGTTGGATGTGCACCAATGCCACTGTAAG	ACGTTGGATGCACATCTGCTAAATGCGTTG	F	acccATGCCACTGTAAGTATTTC
Pa7_14944852	GWAS	7	14944852	A	G	ACGTTGGATGCCTCCGGAGGCATAGTTTAC	ACGTTGGATGGATAGCATGGGCCTCTCAAG	F	cAGATGGATCCAGAAAGGC
Pa7_14956008	GWAS	7	14956008	C	A	ACGTTGGATGGGACTGAGAAGAAGAGAGTG	ACGTTGGATGGCAAAGGGAACTAAAGAGCG	F	gagtTGACGTGAGACTGCTGCT

Pa7_14972283	GWAS	7	14972283	A	G	ACGTTGGATGACTGCCCTGCATCAAAGGAC	ACGTTGGATGTCCTGGAAAAGGGCTATCTC	F	ggggTGCATCAAAGGACTAATTT
Pa7_15008826	GWAS	7	15008826	A	G	ACGTTGGATGTAGGACCATCAGGTGCATTG	ACGTTGGATGGGTACTCTGATAGGCCAAAG	R	GCCAAAGATGGACTACAC
Pa7_15017649	GWAS	7	15017649	G	A	ACGTTGGATGTCCACCAACAGGTTTCTAAG	ACGTTGGATGCTGCACTCAAAACTTGTAACC	F	gGAGATTTTTCGGCTAAAATTTC
Pa7_15101968	GWAS	7	15101968	G	A	ACGTTGGATGCAAGGTTTCAAACCCTTGGC	ACGTTGGATGAGACCCGACTTGTTGCTTAC	R	TGTTGCTTACTCTTGCC
Pa7_15196176	GWAS	7	15196176	C	T	ACGTTGGATGGCGTAAAGTCCAACCATTTC	ACGTTGGATGAGAGTTCTGTGAAAATGGAC	F	ccaatTCCAACCATTTCTATCACA
Pa7_15909400	GWAS	7	15909400	C	T	ACGTTGGATGAAATGTAGCCTGCACCGCAC	ACGTTGGATGGTGCCCCGAGTCTATTTTTC	R	cctTCTTGCCCGATTCCTAT
Marker313919	QTL	7	11325178	G	A	ACGTTGGATGCCAATGCAATCAAGTGTAGC	ACGTTGGATGACGTTGGTTGATGGTATCAG	F	cccCTACAATCAAAAGACTCAGAC
Marker334902	QTL	7	11386582	C	A	ACGTTGGATGCACAGAAATGGAAAACATTG	ACGTTGGATGGCACCTGTAAGTTCAACCTC	R	tTAAGTTCAACCTCTTGATTATATA
Marker339371	QTL	7	11068679	C	A	ACGTTGGATGCGGTGCTAATCAGGAGAACG	ACGTTGGATGCCAGCTTTGAACCACTTTGC	R	taaggTTTCTGTTGCAGTCTGTTGT
Marker359157	QTL	7	11255818	T	G	ACGTTGGATGTTCGCTTAAAAAGTGTGACC	ACGTTGGATGACCACATTTACATAACACCG	R	ccTACATAACACCGTACACTAT
Marker398293	QTL	7	10944436	G	A	ACGTTGGATGGATCCCACCATATTCAGTTC	ACGTTGGATGAGAGAGGCAAAAAATAAGGG	R	GTTTAAACTGTGTCTATACAAATC
Marker436423	QTL	7	10540797	G	A	ACGTTGGATGGCCCCAAATAATTGGCCAAC	ACGTTGGATGGTAGATCCTAAATTCCTAGTC	F	ctttCCAACTCTGTTCTATTTAGTCC
Marker437413	QTL	7	11037771	C	T	ACGTTGGATGTTGTTCCCTTATTAGAGCTG	ACGTTGGATGACCCCATTCAATGCAAGCTC	F	tTTAGAGCTGTCGCGG
Marker442696	QTL	7	11066835	G	A	ACGTTGGATGGAAGAGAGTGGAAAGCAGAC	ACGTTGGATGGGTTTATCTATCATGCTTGG	R	CTTGGTCTCCCCCTT

Table S2 Primer sequences used for RT-qPCR.

Gene ID	Homologous gene	Primer name	Sequence	Amplicon Length	TM
**Pm024213**	*PmTrx*	F	CAAGGATGACTATGACAGGGC	127	60
		R	CCTCAGCCTACGAAGCTTAAA		
**Pm005182**	*IAA26*	F	AGCCTTCCCATTTTCAGTGTC	145	60
		R	TGATCAGAGCCATGAACTGC		
**Pm011163**	*ATGA2OX1*	F	CAAAACCAGACTCCAAACAGC	113	60
		R	GGCCTCAGATTCCAACCTG		
**Pm012630**	*ATAF2*	F	CGGATGAGGAGTTGGTCAATTA	116	60
		R	GGTAGCTGCCAAGGATCAAA		
**Pm012998**	*RVE1*	F	CCATTCCATAAGCCACAAGC	134	60
		R	CATTTGTCACCGCTTTCCTC		
**Pm013791**	*WRKY40*	F	ACAATCATTCCCACCCTTCTC	143	60
		R	AGCACTGGACTTGGATTTGG		
**Pm021243**	*CH3.1*	F	TGAGGACTTGATGGATTACGC	140	60
		R	GCGGTAAAGATGGGCTAAAATG		
**Pm023083**	*UGT73C2*	F	GACGAGGTGATTGAGTGGTTAG	108	60
		R	CTCTGCCAGTATTGAGAACTCC		
**Pm028731**	*LHY*	F	GAGAAGGAGGCACACGATAAA	135	60
		R	TCGAGGATAAGGATTGCTTGG		
**Pm029452**	*PAT1*	F	CCGCATCCACATTATAGACTTCC	139	60
		R	ACGGGCATATTGAGAAAGAGG		

Table S3 Phenotypic variations in seven weeping sub-traits in the F1 population and GWAS panel of *P. mume* .

Population	Trait	NO.	Max	Min	average	SD	C.V.(%)
LP	A1	219	94.29	28.88	58.33	31.58	54.13
	A2	342	104.85	28.65	66.39	18.80	28.32
	T1	228	64.43	25.45	44.58	21.77	48.84
	T2	228	81.94	18.37	45.32	23.82	52.56
	T3	228	102.44	15.90	47.46	27.57	58.09
	T4	228	109.64	12.01	49.39	31.27	63.31
	T5	228	118.37	9.92	51.64	34.97	67.71
AP	A1	214	89.22	21.48	50.67	12.84	25.35
	A2	214	86.56	30.82	53.72	11.89	22.13
	T1	214	120.22	13.87	44.03	15.50	35.20
	T2	214	137.13	12.30	44.85	20.10	44.80
	T3	214	150.46	12.45	45.96	24.44	53.18
	T4	214	161.28	9.97	45.93	27.91	60.78
	T5	214	159.67	6.90	45.75	31.44	68.73

LP,linkage Population; AP, associated population; A1, A2, and T1 to T5 indicate the subtraits descriped in Figure S3; NO., number of samples; SD, standard deviation; C.V., cofficient of phenotypic variation.

Table S4 Significant SNPs that were repeatedly associated with more than two sub-traits.

Sub-traits	Marker	Chromosome	Position	df	F	*P*-value
A2	Pa7_14217507	7	14217507	1	34.993	1.54E-08
A2	Pa7_14217509	7	14217509	1	34.993	1.54E-08
A2	Pa7_14217567	7	14217567	1	35.840	1.07E-08
A2	Pa7_14217897	7	14217897	1	34.783	1.75E-08
A2	Pa7_14218493	7	14218493	1	35.443	1.31E-08
A2	Pa7_14223169	7	14223169	1	35.067	1.56E-08
A2	Pa7_14225796	7	14225796	1	35.040	1.59E-08
T3	Pa7_14129379	7	14129379	1	47.196	8.13E-11
T3	Pa7_14134891	7	14134891	2	25.791	1.11E-10
T3	Pa7_14617059	7	14617059	2	25.791	1.11E-10
T3	Pa7_14617210	7	14617210	1	47.410	7.36E-11
T4	Pa7_11182911	7	11182911	2	20.094	1.1343E-08
T4	Pa7_11267403	7	11267403	2	21.018	5.2768E-09
T4	Pa7_11727246	7	11727246	1	38.670	2.8987E-09
T4	Pa7_11727247	7	11727247	1	38.670	2.8987E-09
T4	Pa7_11730204	7	11730204	1	38.670	2.8987E-09
T4	Pa7_11735990	7	11735990	1	38.670	2.8987E-09
T4	Pa7_11736217	7	11736217	1	38.670	2.8987E-09
T4	Pa7_14136457	7	14136457	2	22.199	2.0016E-09
T4	Pa7_14166974	7	14166974	1	36.549	7.3918E-09
T4	Pa7_14197225	7	14197225	1	39.267	2.3392E-09
T4	Pa7_14205958	7	14205958	1	38.750	2.8007E-09
T4	Pa7_14212408	7	14212408	1	38.750	2.8007E-09
T4	Pa7_14218353	7	14218353	1	35.544	1.299E-08
T4	Pa7_14222311	7	14222311	1	37.422	5.8356E-09
T4	Pa7_14223599	7	14223599	1	37.607	5.3949E-09
T4	Pa7_14223731	7	14223731	1	37.768	4.864E-09
T4	Pa7_14223762	7	14223762	1	38.032	4.3092E-09
T4	Pa7_14617535	7	14617535	1	51.319	1.4924E-11
T5	Pa7_169981	7	169981	2	20.950	5.5852E-09
T5	Pa7_14225796	7	14225796	1	42.580	6.5972E-10
T5	Pa7_14225828	7	14225828	1	44.397	3.0839E-10
T5	Pa7_14225853	7	14225853	1	42.866	5.7953E-10
T5	Pa7_14229948	7	14229948	1	43.229	4.1899E-10
T5	Pa7_14235493	7	14235493	1	64.203	1.0227E-13
T5	Pa7_14236467	7	14236467	1	50.821	1.8261E-11
T5	Pa7_14261326	7	14261326	1	66.685	3.6085E-14
T5	Pa7_14541806	7	14541806	2	34.891	1.0458E-13
T5	Pa7_14645788	7	14645788	2	30.436	2.9812E-12
T5	Pa7_14645837	7	14645837	2	30.436	2.9812E-12
T5	Pa7_14878492	7	14878492	2	25.822	1.0841E-10
T5	Pa7_14881413	7	14881413	1	47.818	6.2244E-11
T5	Pa7_14881634	7	14881634	2	27.539	2.8029E-11

T5	Pa7_14887124	7	14887124	2	33.710	2.5141E-13
T5	Pa7_14900317	7	14900317	1	51.369	1.462E-11
T5	Pa7_14900759	7	14900759	1	47.877	6.077E-11
T5	Pa7_14900984	7	14900984	1	53.306	6.698E-12
T5	Pa7_14901094	7	14901094	1	54.559	4.0557E-12
T5	Pa7_14901221	7	14901221	1	66.685	3.6085E-14
T5	Pa7_14901423	7	14901423	1	55.357	2.9507E-12
T5	Pa7_14901431	7	14901431	1	55.357	2.9507E-12
T5	Pa7_14903156	7	14903156	1	66.685	3.6085E-14
T5	Pa7_14904490	7	14904490	2	29.273	7.2883E-12
T5	Pa7_14907988	7	14907988	2	20.608	7.4086E-09
T5	Pa7_14914486	7	14914486	1	66.685	3.6085E-14
T5	Pa7_14922257	7	14922257	1	66.685	3.6085E-14
T5	Pa7_14932166	7	14932166	2	34.449	1.4515E-13
T5	Pa7_14940427	7	14940427	2	33.191	5.2485E-13
T5	Pa7_14944852	7	14944852	1	66.685	3.6085E-14
T5	Pa7_14956008	7	14956008	2	33.352	3.2864E-13
T5	Pa7_14957283	7	14957283	2	26.404	6.8385E-11
T5	Pa7_14957287	7	14957287	2	26.404	6.8385E-11
T5	Pa7_14957349	7	14957349	2	26.404	6.8385E-11
T5	Pa7_14962781	7	14962781	2	30.023	4.0917E-12
T5	Pa7_14963210	7	14963210	1	66.685	3.6085E-14
T5	Pa7_14963380	7	14963380	1	66.685	3.6085E-14
T5	Pa7_14965870	7	14965870	1	66.685	3.6085E-14
T5	Pa7_14966294	7	14966294	2	33.352	3.2864E-13
T5	Pa7_14972283	7	14972283	2	33.352	3.2864E-13
T5	Pa7_14979828	7	14979828	1	50.112	2.4356E-11
T5	Pa7_14981165	7	14981165	1	44.968	2.025E-10
T5	Pa7_14994881	7	14994881	2	22.973	1.0659E-09
T5	Pa7_14995599	7	14995599	2	22.973	1.0659E-09
T5	Pa7_15008826	7	15008826	1	66.685	3.6085E-14
T5	Pa7_15015061	7	15015061	1	39.132	2.3785E-09
T5	Pa7_15017649	7	15017649	1	40.097	1.5751E-09
T5	Pa7_15019735	7	15019735	1	40.097	1.5751E-09
T5	Pa7_15020606	7	15020606	1	44.113	2.8927E-10
T5	Pa7_15023929	7	15023929	1	60.334	4.1561E-13
T5	Pa7_15025785	7	15025785	2	24.815	2.4459E-10
T5	Pa7_15051882	7	15051882	1	40.788	1.1743E-09
T5	Pa7_15058077	7	15058077	2	24.836	2.3779E-10
T5	Pa7_15058112	7	15058112	2	23.469	7.131E-10
T5	Pa7_15058136	7	15058136	2	24.836	2.3779E-10
T5	Pa7_15058352	7	15058352	2	23.595	6.4419E-10
T5	Pa7_15059677	7	15059677	2	22.575	1.4737E-09
T5	Pa7_15084029	7	15084029	1	66.685	3.6085E-14
T5	Pa7_15086873	7	15086873	1	66.685	3.6085E-14
T5	Pa7_15087768	7	15087768	1	55.300	3.0182E-12

T5	Pa7_15089087	7	15089087	1	66.685	3.6085E-14
T5	Pa7_15090256	7	15090256	1	66.685	3.6085E-14
T5	Pa7_15097886	7	15097886	1	60.334	4.1561E-13
T5	Pa7_15097987	7	15097987	1	41.186	9.9168E-10
T5	Pa7_15100343	7	15100343	1	51.034	1.6748E-11
T5	Pa7_15101968	7	15101968	1	66.685	3.6085E-14
T5	Pa7_15102030	7	15102030	2	22.717	1.3131E-09
T5	Pa7_15102273	7	15102273	1	54.046	4.9789E-12
T5	Pa7_15102480	7	15102480	1	54.443	4.2472E-12
T5	Pa7_15103250	7	15103250	1	66.685	3.6085E-14
T5	Pa7_15108075	7	15108075	1	66.685	3.6085E-14
T5	Pa7_15108640	7	15108640	1	66.685	3.6085E-14
T5	Pa7_15196176	7	15196176	1	60.334	4.1561E-13
T5	Pa7_15197888	7	15197888	1	60.334	4.1561E-13
T5	Pa7_15197889	7	15197889	1	60.334	4.1561E-13
T5	Pa7_15211587	7	15211587	1	60.334	4.1561E-13
T5	Pa7_15224611	7	15224611	1	60.334	4.1561E-13
T5	Pa7_15257716	7	15257716	2	23.128	9.4042E-10
T5	Pa7_15447986	7	15447986	2	25.803	1.1004E-10
T5	Pa7_15455701	7	15455701	1	42.556	5.5594E-10
T5	Pa7_15483191	7	15483191	2	22.509	1.5548E-09
T5	Pa7_15489199	7	15489199	1	42.556	5.5594E-10
T5	Pa7_15513292	7	15513292	1	42.556	5.5594E-10
T5	Pa7_15516119	7	15516119	1	42.556	5.5594E-10
T5	Pa7_15516862	7	15516862	1	42.556	5.5594E-10
T5	Pa7_15517341	7	15517341	2	22.260	1.9051E-09
T5	Pa7_15521078	7	15521078	2	21.737	2.9229E-09
T5	Pa7_15524064	7	15524064	1	42.556	5.5594E-10
T5	Pa7_15524238	7	15524238	2	21.772	2.839E-09
T5	Pa7_15524259	7	15524259	2	21.772	2.839E-09
T5	Pa7_15529191	7	15529191	2	22.086	2.1955E-09
T5	Pa7_15533558	7	15533558	1	42.556	5.5594E-10
T5	Pa7_15535077	7	15535077	1	42.556	5.5594E-10
T5	Pa7_15537997	7	15537997	1	42.556	5.5594E-10
T5	Pa7_15594733	7	15594733	2	19.900	1.3327E-08
T5	Pa7_16496348	7	16496348	2	22.331	1.7976E-09

Table S5 Comparison of variants in the *BW7.1* overlapping region (10.90–11.18 Mb) between weeping and upright sub-populations.

Chr	Start	End	Weeping		Upright	
			No. of variants	-log(*Pi*)	No. of variants	-log(*Pi*)
Pa7	10900001	10910000	22	3.643	--	--
Pa7	11030001	11040000	14	3.776	--	--
Pa7	11090001	11100000	5	4.441	21	4.109
Pa7	11100001	11110000	91	3.632	43	3.978
Pa7	11120001	11130000	6	5.070	--	--
Pa7	11130001	11140000	1	6.149	--	--
Pa7	11160001	11170000	42	4.055	7	4.891
Pa7	11170001	11180000	79	3.508	--	--

Pi, nucleotide diversity; No., number; Chr, chromosome

Table S6 Identification of significant SNPs by integrating GWAS P-value (cut-off < 3 × 10−7) and FST (cut-off > 0.4).

SNP ID	Chr	Position	-log10(*P*)	F_{ST}	*Pi* (Weeping)	*Pi* (Upright)	Fre (Weeping)		Fre (Upright)	
							Allele1	Allele2	Allele1	Allele2
Pa7_11182911	7	11182911	9.110	0.447	0.509	0.077	C:0.5	A:0.5	C:0.968421	A:0.0315789
Pa7_11267974	7	11267974	7.481	0.495	0.499	0.101	C:0.431034	T:0.568966	C:0.95	T:0.05
Pa7_11275091	7	11275091	7.230	0.495	0.499	0.101	C:0.431034	T:0.568966	C:0.947368	T:0.0526316
Pa7_11276453	7	11276453	6.596	0.495	0.499	0.101	T:0.431034	G:0.568966	T:0.939474	G:0.0605263
Pa7_11276936	7	11276936	6.549	0.470	0.499	0.125	A:0.431034	G:0.568966	A:0.936842	G:0.0631579
Pa7_11727169	7	11727169	8.300	0.420	0.503	0.052	T:0.551724	A:0.448276	T:0.971053	A:0.0289474
Pa7_11727246	7	11727246	9.838	0.420	0.503	0.052	C:0.551724	T:0.448276	C:0.976316	T:0.0236842
Pa7_11727247	7	11727247	9.838	0.420	0.503	0.052	C:0.551724	T:0.448276	C:0.976316	T:0.0236842
Pa7_11727711	7	11727711	8.233	0.420	0.503	0.052	A:0.551724	G:0.448276	A:0.971053	G:0.0289474
Pa7_11728788	7	11728788	7.950	0.420	0.503	0.052	G:0.551724	A:0.448276	G:0.971053	A:0.0289474
Pa7_11728912	7	11728912	8.281	0.438	0.506	0.052	T:0.534483	G:0.465517	T:0.973684	G:0.0263158
Pa7_11729661	7	11729661	8.767	0.420	0.503	0.052	A:0.551724	G:0.448276	A:0.971053	G:0.0289474
Pa7_11730521	7	11730521	8.066	0.401	0.499	0.052	C:0.568966	T:0.431034	C:0.971053	T:0.0289474
Pa7_11730706	7	11730706	8.942	0.438	0.506	0.052	T:0.534483	C:0.465517	T:0.973684	C:0.0263158
Pa7_11730822	7	11730822	8.272	0.438	0.506	0.052	A:0.534483	G:0.465517	A:0.973684	G:0.0263158
Pa7_11735007	7	11735007	7.095	0.419	0.503	0.052	T:0.551724	C:0.448276	T:0.973684	C:0.0263158
Pa7_11737148	7	11737148	7.266	0.409	0.506	0.077	C:0.534483	T:0.465517	C:0.960526	T:0.0394737
Pa7_11738066	7	11738066	8.592	0.420	0.503	0.052	C:0.551724	T:0.448276	C:0.976316	T:0.0236842
Pa7_11738332	7	11738332	7.005	0.457	0.507	0.029	C:0.537037	G:0.462963	C:0.971264	G:0.0287356
Pa7_11738691	7	11738691	7.000	0.419	0.503	0.052	C:0.551724	T:0.448276	C:0.968254	T:0.031746
Pa7_11738761	7	11738761	8.075	0.409	0.506	0.077	G:0.534483	T:0.465517	G:0.971053	T:0.0289474
Pa7_12091015	7	12091015	7.701	0.513	0.494	0.101	T:0.413793	A:0.586207	T:0.928947	A:0.0710526
Pa7_12094298	7	12094298	6.746	0.418	0.494	0.191	G:0.413793	A:0.586207	G:0.894737	A:0.105263
Pa7_14529058	7	14529058	7.818	0.401	0.499	0.052	T:0.568966	A:0.431034	T:0.978947	A:0.0210526

Pa7_14543221	7	14543221	7.818	0.401	0.499	0.052	C:0.568966	T:0.431034	C:0.978947	T:0.0210526
Pa7_14560259	7	14560259	7.788	0.541	0.486	0.086	G:0.392857	A:0.607143	G:0.977654	A:0.0223464
Pa7_14560284	7	14560284	6.852	0.471	0.506	0.081	G:0.465517	A:0.534483	G:0.975543	A:0.0244565
Pa7_14560786	7	14560786	6.586	0.495	0.499	0.086	C:0.431034	T:0.568966	C:0.980226	T:0.019774
Pa7_14562344	7	14562344	7.818	0.401	0.499	0.052	T:0.568966	G:0.431034	T:0.978947	G:0.0210526
Pa7_14562431	7	14562431	8.043	0.401	0.499	0.052	G:0.568966	T:0.431034	G:0.981579	T:0.0184211
Pa7_14565930	7	14565930	7.818	0.401	0.499	0.052	G:0.568966	C:0.431034	G:0.978947	C:0.0210526
Pa7_14568407	7	14568407	7.818	0.401	0.499	0.052	T:0.568966	C:0.431034	T:0.978947	C:0.0210526
Pa7_14569777	7	14569777	7.818	0.401	0.499	0.052	A:0.568966	T:0.431034	A:0.978947	T:0.0210526
Pa7_14853605	7	14853605	6.924	0.401	0.503	0.169	C:0.448276	T:0.551724	C:0.939474	T:0.0605263
Pa7_14999403	7	14999403	7.790	0.437	0.506	0.052	C:0.534483	T:0.465517	C:0.981481	T:0.0185185
Pa7_14999481	7	14999481	6.759	0.426	0.508	0.077	G:0.517241	A:0.482759	G:0.97619	A:0.0238095
Pa7_14999509	7	14999509	7.268	0.408	0.506	0.077	A:0.534483	C:0.465517	A:0.978836	C:0.021164
Pa7_15016715	7	15016715	7.930	0.400	0.499	0.052	A:0.568966	T:0.431034	A:0.971053	T:0.0289474
Pa7_15058112	7	15058112	9.147	0.407	0.506	0.077	C:0.534483	T:0.465517	C:0.981579	T:0.0184211
Pa7_15106933	7	15106933	7.817	0.401	0.499	0.052	G:0.568966	T:0.431034	G:0.981579	T:0.0184211
Pa7_15525580	7	15525580	8.141	0.428	0.506	0.053	C:0.534483	T:0.465517	C:0.986188	T:0.0138122
Pa7_16645510	7	16645510	6.909	0.417	0.503	0.052	G:0.551724	A:0.448276	G:0.965789	A:0.0342105

P, GWA P-value; *F* ST, genetic differentiation; *Pi* , ucleotide diversity; Fre, allele frequency; Chr, chromosome

Table S7 Quantitative locus detection for seven sub-traits in the F1 population derived from 'Liuban' × 'Fentai Chuizhi'.

Sub-traits	No. of overlaping intervals	Segregating type	Significant peak	QTL name	LGs	Range of interval size (cM)	Peak marker	Peak position (cM)	Chr position	LOD	PVE(%)
T2/T3	2	<hkxhk>	qtl-Bt2.1	QTL-LG2.1	2	33.46-37.96	Marker1423266	36.7	Pa2:2190052-4077368	4.1-4.2	1.8-2.2
A1	1	<hkxhk>	qtl-Ba2.1	QTL-LG2.2	2	65.96-68.96	Marker1087019	67.7	Pa2:7712494-8302782	4.8	2.3
A2	1	<hkxhk>	qtl-Ba3.1	QTL-LG3.1	3	16.69-17.94	Marker157936	17.2	Pa3:449053-774157	3.2	3.3
T3	1	<hkxhk>	qtl-Bt3.1	QTL-LG3.2	3	97.94-99.94	Marker41967	98.7	Pa3:12066065-12234547	4.0	5.1
T3	1	<hkxhk>	qtl-Bt3.2	QTL-LG3.2	3	100.44-100.94	Marker158471	100.7	Pa3:12234317-12332976	3.2	2.4
A1	1	<hkxhk>	qtl-Ba4.1	QTL-LG4.1	4	27.55-28.55	Marker688337	28.3	Pa4:1969911-2272773	3.0	3.5
A1	1	<hkxhk>	qtl-Ba5.1	QTL-LG5.1	5	74.52-77.02	Marker978200	74.8	Pa5:11553222-15778445	4.5	1.2
A2	1	<mlxmm>	qtl-Ba5.2	QTL-LG5.2	5	133.90-134.40	Marker857908	134.2	Pa5:19918394-19922253	18.3	4.9
A2	1	<mlxmm>	qtl-Ba5.3	QTL-LG5.2	5	135.90-136.90	Marker973436	136.2	Pa5:19921980-19962433	22.1	4.7
A2	1	<mlxmm>	qtl-Ba5.4	QTL-LG5.2	5	148.40-148.90	Marker980906	148.7	Pa5:21391637-21401427	13.9	2.8
A1/T1-5	6	<mlxmm>	qtl-Bat7.1	QTL-LG7.1	7	69.25-69.75	Marker431969	69.5	Pa7:10540978-10549292	30.5-44.0	45.1-55.3
A2	1	<mlxmm>	qtl-Ba7.1	QTL-LG7.1	7	71.25-71.75	Marker430976	71.5	Pa7:10801041-10853189	62.2704	19.8922
A1/T1-5	6	<hkxhk>	qtl-Bat7.2	QTL-LG7.1	7	70.24-71.74	Marker422078	71.5	Pa7:9609083-10945426	17.4-33.3	11.9-19.2
A1/T4-5	3	<hkxhk>	qtl-Bat7.3	QTL-LG7.1	7	72.24-72.74	Marker339371	72.5	Pa7:10945139-11256053	16.9-35.4	23.6-24.8
A1-2/T1-5	7	<hkxhk>	qtl-Bat7.4	QTL-LG7.1	7	73.24-73.74	Marker313919	73.5	Pa7:11255828-11565667	18.5-46.5	11-33.9
A2	1	<hkxhk>	qtl-Ba7.2	QTL-LG7.1	7	76.24-76.74	Marker312132	76.5	Pa7:11565444-11565917	29.2	10.8

LGs: Lingkage groups; PVE: Variation explained; LOD: logarithm of odds ; QTL, quantitative trait locus; Chr, chromosome

Peak marker: Peak marker refers to the co-domiant marker that is closest to the QTL peak

Table S8 Fifty-five epistatic loci of the marker-marker, marker-QTL and QTL-QTL types for seven sub-traits.

Sub-traits	Repeats	Genetic position1	LGs	Chr postion 1	Position 2	LG	Chr positon 2	Type of epistasis	LOD	PVE (%)
T1	1	71.213	LG2	Pa2:7712494-8302782	76.21	LG2	Pa2:8914323-8972887	marker-QTL	5.326	7.190
T1	1	86.213	LG2	Pa2:10895829-10932294	176.69	LG3	Pa3:20210308-20816418	marker-marker	5.828	3.881
A1	1	125.505	LG1	Pa1:16527644-17817037	96.14	LG6	Pa6:11819306-12475897	marker-marker	4.207	3.160
T2/T3	2	72.489	LG7	Pa7:10945139-11256053	30.87	LG8	Pa8:3180611-3847483	marker-QTL	23.517	6.766
A1	1	178.265	LG5	Pa5:21834722-23127948	70.87	LG8	Pa8:9648443-10611869	marker-QTL	4.714	3.224
A1	1	62.489	LG7	Pa7:9266534-9609329	70.87	LG8	Pa8:9648443-10611869	marker-QTL	7.201	3.760
T1	1	62.505	LG1	Pa1:5355698-6220557	95.87	LG8	Pa8:12941578-13073308	marker-marker	6.852	4.590
A2	1	74.489	LG7	Pa7:11255828-11565667	106.87	LG8	Pa8:14894383-15509788	marker-QTL	10.158	7.226
T1	1	176.685	LG3	Pa3:20210308-20816418	211.69	LG3	Pa3:23160795-23488472	marker-marker	5.679	6.282
T3/T4/T5	3	75.00	LG7	Pa7:11324965-11386663	100.00	LG7	Pa7:13404451-13470264	QTL-QTL	7.258	31.601
T3/T4/T5	3	24.30	LG3	Pa3:1400831-1430063	75.00	LG7	Pa7:11324965-11386663	marker-QTL	5.010	23.495
T5	1	69.70	LG4	Pa4:11628408-11728131	75.00	LG7	Pa7:11324965-11386663	marker-QTL	5.627	7.457
A1	1	74.489	LG7	Pa7:11255828-11565667	77.49	LG7	Pa7:11565672-11597378	QTL-QTL	6.936	7.395
T1	1	88.265	LG5	Pa5:15778209-16593550	77.49	LG7	Pa7:11565672-11597378	QTL-QTL	5.245	7.139
A2	1	74.489	LG7	Pa7:11255828-11565667	101.49	LG7	Pa7:13117575-13750422	QTL-QTL	11.752	7.336
A1	1	215.213	LG2	Pa2:32920691-33869525	62.49	LG7	Pa7:9266534-9609329	marker-QTL	7.564	4.195
A1	1	175.685	LG3	Pa3:20210308-20816418	62.49	LG7	Pa7:9266534-9609329	marker-QTL	6.476	3.349
T1	1	26.213	LG2	Pa2:2190052-4077368	62.49	LG7	Pa7:9266534-9609329	marker-QTL	5.459	4.961
A1	1	105.135	LG6	Pa6:13716528-14948312	62.49	LG7	Pa7:9266534-9609329	marker-QTL	6.504	3.956
A1	1	47.505	LG1	Pa1:2150686-4630341	62.49	LG7	Pa7:9266534-9609329	marker-QTL	8.479	4.191

A1	1	186.795	LG4	Pa4:22033862-22440019	62.49	LG7	Pa7:9266534-9609329	marker-QTL	7.507	3.952
A1	1	88.265	LG5	Pa5:15778209-16593550	62.49	LG7	Pa7:9266534-9609329	QTL-QTL	8.531	3.838
A2	1	30.00	LG7	Pa7:2611048-2627842	33.00	LG7	Pa7:3157892-3351129	marker-marker	3.670	9.379
T1	1	147.505	LG1	Pa1:19880772-20100393	27.49	LG7	Pa7:2186186-2669376	marker-marker	5.065	3.855
T3	1	72.489	LG7	Pa7:10945139-11256053	87.49	LG7	Pa7:12416773-12521948	marker-QTL	21.473	6.641
T4/T5	2	226.213	LG2	Pa2:35368874-36670096	72.49	LG7	Pa7:10945139-11256053	marker-QTL	36.260	7.400
T2/T3	2	221.213	LG2	Pa2:34586048-34739076	72.49	LG7	Pa7:10945139-11256053	marker-QTL	26.449	6.838
T3/T5	2	86.685	LG3	Pa3:9980878-10537414	72.49	LG7	Pa7:10945139-11256053	marker-QTL	38.549	7.492
T4	1	79.135	LG6	Pa6:10075009-10665573	72.49	LG7	Pa7:10945139-11256053	marker-QTL	27.736	6.294
T2/T3	2	74.135	LG6	Pa6:9658762-9809612	72.49	LG7	Pa7:10945139-11256053	marker-QTL	24.701	6.734
T2	1	122.505	LG1	Pa1:16527644-17817037	72.49	LG7	Pa7:10945139-11256053	marker-QTL	13.869	6.033
T3	1	147.505	LG1	Pa1:19880772-20100393	72.49	LG7	Pa7:10945139-11256053	marker-QTL	24.458	6.874
T4	1	142.505	LG1	Pa1:19024463-19672251	72.49	LG7	Pa7:10945139-11256053	marker-QTL	33.095	6.468
T1/T2	2	67.489	LG7	Pa7:9609083-10945426	72.49	LG7	Pa7:10945139-11256053	QTL-QTL	10.194	13.892
T4/T5	2	27.489	LG7	Pa7:2186186-2669376	72.49	LG7	Pa7:10945139-11256053	marker-QTL	40.808	7.350
T4	1	30.795	LG4	Pa4:2396983-2899349	72.49	LG7	Pa7:10945139-11256053	marker-QTL	21.862	6.407
T4	1	76.685	LG3	Pa3:9328009-9458158	72.49	LG7	Pa7:10945139-11256053	marker-QTL	30.713	6.307
T2/T3	2	60.795	LG4	Pa4:6720737-7551315	72.49	LG7	Pa7:10945139-11256053	marker-QTL	26.383	6.835
T2/T3	2	128.265	LG5	Pa5:19541991-19961874	72.49	LG7	Pa7:10945139-11256053	QTL-QTL	23.055	6.847
T4	1	68.265	LG5	Pa5:7967119-10066783	72.49	LG7	Pa7:10945139-11256053	marker-QTL	18.261	6.258
T2	1	56.685	LG3	Pa3:7135374-7338330	72.49	LG7	Pa7:10945139-11256053	QTL-QTL	14.837	6.117
A2	1	221.213	LG2	Pa2:34586048-34739076	74.49	LG7	Pa7:11255828-11565667	marker-QTL	11.808	7.352

A2	1	40.685	LG3	Pa3:3750942-4008885	74.49	LG7	Pa7:11255828-11565667	marker-QTL	11.750	7.323
A2	1	96.135	LG6	Pa6:11819306-12475897	74.49	LG7	Pa7:11255828-11565667	marker-QTL	13.273	7.565
A2	1	125.505	LG1	Pa1:16527644-17817037	74.49	LG7	Pa7:11255828-11565667	marker-QTL	11.040	7.266
A2	1	159.795	LG4	Pa4:19592754-19782442	74.49	LG7	Pa7:11255828-11565667	QTL-QTL	12.033	7.298
A2	1	139.265	LG5	Pa5:20104036-20132893	74.49	LG7	Pa7:11255828-11565667	QTL-QTL	11.387	7.273
A1	1	69.00	LG7	Pa7:10490308-10541206	96.00	LG7	Pa7:13001499-13153051	marker-QTL	3.193	20.280
T4/T5	2	43.17	LG2	Pa2:5117364-5174687	60.00	LG7	Pa7:8550505-8603275	marker-marker	6.209	7.119
T5	1	65.00	LG6	Pa6:8691302-8858389	60.00	LG7	Pa7:8550505-8603275	marker-marker	5.249	5.865
T5	1	45.00	LG1	Pa1:3795373-3877205	60.00	LG7	Pa7:8550505-8603275	marker-marker	5.159	5.882
T4/T5	2	28.15	LG5	Pa5:2298889-2351333	60.00	LG7	Pa7:8550505-8603275	marker-QTL	5.072	8.193
T1	1	26.685	LG3	Pa3:1684273-1960234	145.80	LG4	Pa4:17250863-17671973	marker-marker	5.895	4.910
A1	1	206.213	LG2	Pa2:31925113-32118996	145.27	LG5	Pa5:20724934-20914352	marker-QTL	4.668	3.097
T1	1	86.213	LG2	Pa2:10895829-10932294	223.27	LG5	Pa5:25010852-25982007	marker-marker	5.399	4.062

Chr, chromosome (Pa); LGs, linkage groups; PVE: Variation explained; LOD: logarithm of odds.

Table S9 Functional classification and FPKM values of 85 genes co-expressed with *Pm024213* .

Gene ID	Ortholog of *A. thaliana*	Chromosome position	Annotations	log2(FC) UB *vs* WB	log2(FC) US *vs* WS	Reg	UB_FPKM	US_FPKM	WB_FPKM	WS_FPKM
Pm029537	TSA	locus=scaffold266:329114:329938:-	tryptophan synthase	5.494	4.624	up	1.222	1.362	55.231	32.383
Pm029538	TSA2	locus=scaffold266:338954:339904:-	tryptophan synthase	5.945	3.896	up	1.288	3.311	79.110	48.074
Pm005694	CM2	locus=Pa2:12399857:12401394:-	tryptophan synthase	3.417	2.340	up	4.449	11.139	49.953	61.808
Pm012630	ATAF2	locus=Pa3:21656184:21657564:-	development	2.702	2.956	up	3.528	3.771	22.961	28.914
Pm013167	DUF581	locus=Pa4:2203863:2204551:+	development	3.178	2.819	up	2.729	1.941	24.939	12.682
Pm004758	ATAF1	locus=Pa2:6999344:7000733:+	development	3.418	2.637	up	4.454	4.354	45.373	27.641
Pm009913	FBS1	locus=Pa3:1626434:1627193:+	development	2.783	1.513	up	9.049	13.045	61.440	37.314
Pm_newGene_2032	ILR-like5	locus=scaffold394: 26174:28866:-	auxin	3.153	3.654	up	1.241	0.880	11.286	10.833
Pm013242	LOX3	locus=Pa4:2707168:2711262:+	jasmonate	2.787	1.929	up	13.224	20.478	90.459	76.840
Pm017697	ERF13	locus=Pa5:13354118:13354774:-	ethylene	2.409	3.535	up	1.395	0.516	7.139	5.701
Pm021243	GH3.1	locus=Pa6:7546969:7548992:+	auxin	2.907	3.034	up	0.902	0.839	10.038	6.770
Pm011163	ATGA2OX1	locus=Pa3:9358924:9360357:+	gibberelin	3.270	1.679	up	0.914	2.560	8.862	8.082
Pm_newGene_1784	alpha/beta-Hydrolase	locus=Pa8:9517166:9517800:-	triacylglycerol lipase	5.155	3.468	up	0.100	0.260	2.799	2.737
Pm018937	FAD8	locus=Pa5:21338565:21340691:-	omega 3 desaturase	2.295	1.831	up	27.250	28.643	133.782	101.124
Pm002909	FAD7	locus=Pa1:22145269:22147437:+	omega 3 desaturase	2.240	1.725	up	11.948	12.961	56.895	42.705
Pm008424	A/N-InvC	locus=Pa2:33026638:33030130:-	degradation sucrose	2.082	2.311	up	25.146	13.995	109.391	68.342
Pm001344	CYP94C1	locus=Pa1:9271677:9273182:-	cytochrome P450	5.252	5.427	up	0.977	1.007	35.326	43.265
Pm020435	GDSL-like	locus=Pa6:2987598:2989522:-	GDSL-motif lipase	1.674	1.476	up	3.751	6.953	11.814	19.232
Pm020941	sks5	locus=Pa6:5823352:5826684:-	oxidases-copper,flavone	2.148	2.342	up	1.218	2.152	5.285	10.815
Pm023083	UGT73C2	locus=Pa7:1997951:1999600:-	UDP glucosyltransferases	2.874	2.340	up	7.489	8.417	53.690	42.404
Pm024494	CYP94B1	locus=Pa7:12713060:12714808:-	cytochrome P450	3.161	3.004	up	1.070	1.967	9.427	15.560

Pm003732	GLC	locus=Pa1:26589796:26593016:+	acyl transferases	2.762	2.121	up	1.456	2.502	9.749	10.784
Pm008627	GGP3	locus=Pa2:34611543:34612436:+	polyamin oxidase	4.080	2.356	up	0.605	3.410	9.300	17.207
Pm016490	PAO1	locus=Pa5:1973452:1981052:+	polyamin oxidase	2.600	3.117	up	2.457	1.171	14.704	10.080
Pm018946	AP2C1	locus=Pa5:21383120:21384870:+	postranslational modification	3.063	2.893	up	1.183	0.874	9.797	6.404
Pm019734	HAI1	locus=Pa5:25427928:25429674:-	postranslational modification	2.134	2.113	up	1.377	1.415	5.917	6.092
Pm004462	AT1G78280	locus=Pa2:5168362:5176512:+	ubiquitin E3	1.982	1.872	up	6.858	8.167	27.403	29.828
Pm004762	AT1G44130	locus=Pa2:7031402:7035584:-	degradation	1.998	1.712	up	8.857	11.938	35.334	38.717
Pm008006	AT1G03220	locus=Pa2:30182252:30183568:-	spartate protease	4.419	3.992	up	1.605	1.787	33.895	28.065
Pm013791	WRKY40	locus=Pa4:6725543:6727134:+	transcription	4.145	3.484	up	1.924	2.689	35.183	30.204
Pm014068	bHLH	locus=Pa4:9083094:9084061:-	transcription	5.297	7.007	up	0.386	0.112	14.871	11.968
Pm018401	WRKY53	locus=Pa5:18120250:18122148:+	transcription	1.511	1.694	up	7.454	7.211	22.531	24.955
Pm023766	ERF027	locus=Pa7:8033365:8034192:+	transcription	5.244	6.072	up	0.188	0.135	5.888	7.450
Pm024716	bHLH	locus=Pa7:13872300:13873018:-	transcription	5.541	5.924	up	0.130	0.038	6.189	2.334
Pm029452	PAT1	locus=scaffold265:330024:331790:-	transcription	2.022	1.983	up	3.753	3.427	15.379	13.370
Pm029511	ERF53	locus=scaffold266:12850:14250:-	transcription	3.849	1.567	up	1.065	2.498	15.493	7.402
Pm028829	ABR1	locus=scaffold188:52018:53467:+	transcription	7.401	Infinity	up	0.024	0.000	4.035	2.778
Pm004265	ERF019	locus=Pa2:3620347:3620847:-	transcription	5.736	5.767	up	0.237	0.114	8.974	5.447
Pm005182	IAA26	locus=Pa2:9685220:9690217:+	transcription	1.935	1.385	up	12.414	17.097	47.729	44.439
Pm005698	WRKY18	locus=Pa2:12424777:12426189:-	transcription	5.058	4.226	up	0.745	0.829	24.136	15.018
Pm008389	WRKY48	locus=Pa2:32805980:32807604:+	transcription	2.864	2.023	up	1.457	2.159	10.245	8.681
Pm008898	AT5G57150 (bHLH)	locus=Pa2:36996444:37000388:+	transcription	2.524	2.895	up	11.249	5.935	65.413	43.915
Pm009457	BHLH92	locus=Pa2:41079994:41081531:-	transcription	6.211	6.939	up	0.321	0.128	23.322	13.125
Pm024074	GT72B1	locus=Pa7:10244660:10245529:-	flavonoids	4.153	3.208	up	0.374	0.447	6.292	3.979
Pm028477	TPS14	locus=scaffold1332:7866:10301:+	isoprenoids	2.959	3.327	up	6.292	4.389	50.056	43.546

Pm009849	TPS14	locus=Pa3:1288340:1290782:+	isoprenoids	2.836	2.593	up	1.353	1.471	9.804	8.693
Pm015276	PBP1	locus=Pa4:18500435:18500806:-	signalling	5.033	2.264	up	0.222	1.126	5.514	3.676
Pm015302	MYB14	locus=Pa4:18702258:18703318:+	signalling	3.530	4.205	up	0.616	0.666	6.657	11.414
Pm003131	ACBP60	locus=Pa1:23298870:23302018:-	signalling	2.490	2.687	up	0.974	0.809	5.494	5.118
Pm005628	GLR2.8	locus=Pa2:12049275:12054231:-	signalling	5.349	5.378	up	0.164	0.164	6.429	6.307
Pm005897	AT1G34300	locus=Pa2:13578135:13580534:-	signalling	1.687	2.069	up	2.381	2.032	7.553	8.457
Pm015504	AOC4	locus=Pa4:19914457:19915219:+	stress	2.190	1.631	up	87.344	103.909	399.610	318.664
Pm007487	EP3	locus=Pa2:25274296:25275763:+	stress	3.084	3.000	up	1.204	1.269	9.680	9.500
Pm018750	TIP1;3	locus=Pa5:20348425:20349664:+	transport	3.629	2.010	up	1.361	0.705	16.429	5.664
Pm030112	PIP2;5	locus=scaffold475:133879:135129:-	transport	4.160	2.130	up	3.037	4.703	53.584	20.989
Pm028901	AT4G27745	locus=scaffold1990:11743:12446:+	not assigned	8.900	Infinity	up	0.030	0.000	14.253	20.055
Pm_newGene_1600	unknown	locus=Pa7:8998950:8999409:-	not assigned	6.619	4.002	up	0.102	0.439	9.448	6.060
Pm_newGene_403	unknown	locus=Pa2:19413058:19413735:-	not assigned	5.941	3.804	up	0.245	2.003	33.676	38.948
Pm_newGene_944	unknown	locus=Pa4:15937421:15937780:-	not assigned	2.162	2.689	up	22.303	14.304	102.785	86.755
Pm012535	DUF506	locus=Pa3:20839291:20840516:+	not assigned	2.433	2.940	up	1.116	1.319	9.122	15.251
Pm013228	TIFY10B	locus=Pa4:2621175:2622142:-	not assigned	2.610	3.325	up	22.082	10.044	136.671	99.144
Pm014876	JAZ8	locus=Pa4:15981857:15982905:-	not assigned	N/A	3.758	up	0.256	0.688	2.358	9.397
Pm016533	unknown	locus=Pa5:2354459:2356156:+	not assigned	2.688	1.762	up	8.715	19.214	62.223	71.217
Pm001985	unknown	locus=Pa1:15548575:15548865:-	not assigned	3.990	2.548	up	1.630	5.305	20.905	28.735
Pm020497	AT5G32470	locus=Pa6:3312969:3315625:+	not assigned	4.938	3.471	up	0.121	0.345	3.039	3.726
Pm022713	AT1G31130	locus=Pa6:20108450:20109448:+	not assigned	2.645	2.024	up	0.934	1.765	5.780	7.096
Pm023994	unknown	locus=Pa7:9786848:9787483:-	not assigned	2.360	1.896	up	3.070	4.153	15.626	14.950
Pm026847	DUF241	locus=Pa8:12146066:12146980:+	not assigned	4.245	2.375	up	0.368	1.072	6.348	5.367
Pm031299	unknown	locus=scaffold907:29100:30138:-	not assigned	6.470	6.003	up	0.492	0.577	39.540	32.150
Pm024213	unknown	locus=Pa7:11168079:11173903:+	not assigned	6.885	6.762	up	0.687	0.706	66.401	59.251

Pm000394	AT4G05030	locus=Pa1:2457212:2457725:+	not assigned	3.977	4.420	up	1.476	0.598	21.961	11.587
Pm004622	AT3G11760	locus=Pa2:6135934:6139258:+	not assigned	4.135	1.929	up	0.251	1.367	4.301	5.477
Pm005539	AT5G12010	locus=Pa2:11560288:11561733:+	not assigned	2.660	2.695	up	4.292	3.851	26.681	24.949
Pm005995	NTMC2T5.2	locus=Pa2:14147604:14151916:+	not assigned	2.418	2.188	up	7.957	9.871	43.265	45.026
Pm006199	DUF668	locus=Pa2:15396219:15400115:-	not assigned	1.617	1.799	up	2.920	2.497	9.026	8.663
Pm007189	JAZ10	locus=Pa2:22259348:22260531:+	not assigned	2.385	2.730	up	23.515	18.177	124.179	120.777
Pm008560	AT2G18690	locus=Pa2:34083944:34084900:-	not assigned	2.734	3.141	up	0.761	0.794	5.109	6.587
Pm009527	AT2G32150	locus=Pa2:41516087:41517860:-	not assigned	2.319	2.341	up	13.214	7.289	68.610	41.886
Pm010428	JAZ8	locus=Pa3:4408893:4409718:+	not assigned	4.715	3.884	up	2.879	4.668	75.533	67.279
Pm_newGene_2259	unknown	locus=scaffold265:591030:591232:+	not assigned	Infinity	infinitely	down	26.093	25.805	0.000	0.000
Pm016684	LNK3	locus=Pa5:3800225:3803963:+	not assigned	-3.178	-2.220	down	14.312	11.278	1.612	2.410
Pm030502	AT3G25570	locus=scaffold56:443360:444427:+	SAM decarboxylase	-2.601	-1.691	down	676.464	525.922	113.155	162.451
Pm012998	RVE1	locus=Pa4:1017340:1019721:-	transcription	-3.258	-2.613	down	24.675	26.724	2.596	4.366
Pm028731	LHY	locus=scaffold162:908301:914154:-	transcription	-5.602	-2.849	down	88.710	66.481	1.804	9.256
Pm027248	CCoAMT	locus=Pa8:14284701:14286205:+	lignin biosynthesis	-5.943	-2.691	down	13.334	8.810	0.260	1.376

Reg, regulation; FC, fold-change; UB, bud pools of upright trees; WB, bud pools of weeping trees; US, stem pools of upright trees; WS, stem pools of weeping trees.

(grey shading) indicates the co-expressed genes located in epistatic loci
