# Supplementary material for: Identification of the PmWEEP locus controlling weeping traits in Prunus mume through an integrated genome-wide association study and quantitative trait locus mapping
Source: Hortic Res. 2021 Jun 1;8:131. doi: 10.1038/s41438-021-00573-4 (PMC8167129; doi:10.1038/s41438-021-00573-4)
Supplement: Supplementary file 2 — Supporting Appendix1 [file 41438_2021_573_MOESM2_ESM.docx]

**Appendix S1** Genomic sequences for primers designed for the marker validation assays

>Pa7_11182911 Pa7:11182611-11183211 Ref (Alt)

ATATCACTCTTCTATTCGGAATATTTTCTGGCCCAAATCTTCGGGCTCCGCCGATGACCACTTTTCCATTAGAGCCATTTTTAACCATTAGCCGCCACCTCTCTTCACCAGCAAGCAACGTCCCATTATCCATCATCTTATTTATGAGTAACTTTTGTGACCACAAAATAAAAGCTTATTATTATAAGTATTTTATACACATTTGCGGTCATATAGTAGCAATGGATTCAATTGATCAATGTCAAGTGTATTATCTAGCTCTTAATTTAATGGTGTGTTTCTTTCTAACGAGATTTTTC**C(A)**TCAACTTATGAAATGAGGTTAGTTTGGGCAACGGACTGTGTTTGACAAGCATGGACTGGCGGACCCAGAATTTTTTTAGCGGAGGTGCAATATTGACTAAGTATGAATATGGTTTTTCGGAATAATCATTTTCTCAAGATAATTTTTGGCGGCTTTTATTCCTTATACATCAAATAAGAAAACTTGATTTTATGGGATTTTAGTATGAAGTATATATTGACTTAATGAGCTATCATTTTTAGCCTAAATCGTATTTTGCACTAAAACCGAATTTTCATATTTTTATTTGGTGGGAATTTG

>Pa7_11727711 Pa7:11727411-11728011

AAATACCAGATAGCCAGATTAGTTTGAAGTTGAACCAATCACAGCATCAGCCCAATTGAGAAAATAATGATGCATGTAGCGTACCTGAATCCCTGAGACGGTGAAATACGGGATTTCAAACTTCACACGAATAGGAGCTTTTCTTTCAGGAACTGCTTCTTCAGAAGTTATACTAGGAAGCCTAAACTCAGCCCTCAACATGTACTCCTGTGCAATTTCATACAAGGTCAAAAATATCTTTGCGAGGTCTTTGCAATTGATAATATACTTAATGGCAAGAGAAAGCTTCCTACCTTATT**A(G)**CCAGGAAAAGATTTAATTTTCCAGACTAAAGCATCACTCTCTGGAGCATATGCAGCAGATCCCATTGATGTCCGGACATTAGGATTTGTAGCATCGGCAGGCACAGGCAAATGAATTTCGACATTTGTAGCCGTGCTGTACCAAGTAACCGGAGCTTACAGTAAGAAATTCTGATAATGGAAATTGAGTGAAAAGTTGTAAACTGTTGCAGTAAGCAACTGTTGCAAGCTATAGAAATCATTCAACTGACTTTTTACAATACAGAACATGTATCACTGCTATTACCTGCGCTCCTTATAC

>Pa7_11728912 Pa7:11728612-11729212

AACCATCCAAGGATGAAATCCAATTATATTTACAAATTTTAGGATACTCTTAATTTATATTCATTTTCCAAGCTCAAGTTAACATCACTAAAGCCTTATAATCAGGTGGGATGTTGGTTGATCAGTGTCACTGTCAGGTGACTAACTCAGAGATGAACGTGCTAATAATCATGCCGGAGTTGAATTTTCAAAATCCTAATCATTAACCATAGGGTTGGCCAAACAAATTTTAATTTGGCATGTAGTTTGCCTTCGTAGCAAATGCAGTTGCTACTTTCTAGATTTTTTTGCCATAATAC**T(G)**GAACTGAAGAAGAAGTTATGCACCTCAAATACGTTCTCATCTTTAGGGCCCCCACAACATCTGACCGTATTATTTGTCCATTGCTGTTGACAAGTATATTGACACTTTCCACCACATCCAGAAAGACCTGATATAAAATGAACAGAGTCAATGATGGTTCACACGTAATTTAATTCATCCAAGATTAAACATGAAGAGAGGATCAACCTACTTCATTCTTCTTGTATCGTATCCCTTCGCTGCGCCAAGACACTGCATTTGTGACAGCCATGGGAGGACGCTGTGTCACCTCCATCCTGT

>Pa7_11729661 Pa7:11729361-11729961

TACAAAAGACCTTACAAGTCAATTACCACTACGACAAAGTTGTCCCTTAGTGATTCCTCTTCTAGCTCTTCAAAATAGTGCTTAAATACCTGAACACATAAACAAAACGCATTGAAATGTATGAAAACCTCACATGGGTAACAACGGATGGTGAATGGCAATGCCATTATGCCATTTAAGACAAATACAAGTGAAAAAATTGGCCAGAGTTGAAGAGTTCGTACATCAATTACACGATGCAGAAAGAGCAGCAAGCTAGCAGCATTGCAGTTCTGCCTCGAGGCAATCATCAAGTAGAC**A(G)**TTGTTGTGCTGTATGAACATGTAGCTGACTCCATTGTCGTAAACCACTGGATCTTGGGAGTAAGGATCGACCTGAAGAAGAATATCAAAATTCCCACAAATGCAAAGCTTAAATTTTACCCAGAAATTTCCAAACTTGATCGTTCTAATTGAGAAATTGATGCAAGCTTCATGGTGATCAAAGCTGAATATATATATTATCACATACTTTACGGTCTTCCTGTTGAGGCTGGCAAGCAAAAATGAGAAAGAAAGATTACCTCTTTTTCAATGAACTTGGTGAAGAAGCGCTCGGCCTGAG

>Pa7_11866123 Pa7:11865823-11866423

CGACGGTAATCTCCGTTACGTCGGTGGCCTTACTCGTGTTCTTGCCGTCGATGCCTCCATTTCCTTTGCAGGTTTGTTCAATCTGCTTCAATTCGTTATTTTCCTTTTGATTTTGGCTTAATTTACTTGAATTTCAACTCGATTTCATTTTCTTCGTTGAATTTAGTGCTAATTTCCATTTTTTCGACATGTACAGAGCTAATGGTGAAACTCGCGGAGTTCTGTGGCTACTCTGTGGATTTGAGGTGTCAATTGCCGGATGGAGATTTGGAGACCTTGATTTCGGTCAAGTCCGACGA**G(C)**GAGTTAGCTAATATCATCGAAGAGTACGATCGAGCTTCTTCTTCGTCTCGTCCTCTAAAGATCAGAGCCATTCTCTCGCCGCCGAAATCGCTGAAGCAAATCTCGCCTCCTATGTCTATGGCTACGAGCGGCGGCGATTTGTCCCCTTCGAAATCGCTCTTATCGTTCACCGATTCGCCTCCGAAACGGTATGTTTCGCCTCTGAAAGGGTATGTTTCGCCTCCGAAACGGTACGCTTCGCCGCCGATTGTACATCCCGGGCCAGTCAGGTTTCAGAAAGGGTCCGGACGTGCTTGCTAC

>Pa7_11918846 Pa7:11918546-11919146

ACATAAATAGCTCTATGAAAGTTTGGATAATAAAATAGTAATATTCTCGTTTCATGAAAGTTTTTCTCCAAATTGACACTAGGTTTTAGAGACGTGTCATGCAACAGCAGTATATATTCTTATCCATAATTGTAAGAAAATACATAAAGAAAACATGCACAGTTTGACCAAGGAAGGAATTAGAATAATAAACTCTTAAAAGTGTTATTAAAAATATATTAACATAGCATACCTCCCCATGAGAACTCTTGCACTAGTTGACAACTTGACATATTTTTACATGAACCATCAAACCAGAT**T(C)**GTAAGAAAACAGAAAAGAGAAAAACAAAAAAGATTATTGGTATTGCTATTTCAGAAAGGTTGGTGCTTGGTGCTTGGTGCTTGGTGCTTGGTGGCTTGGTGGCTTGGTGCTGCTGTCCATTATGGGAAGCATTTTAGTTGTACATCTACAACCACACCAACCACTATGTCTGCCATAATATTATTCATTAATTATTTAAAAAATAATGCAATCATGCAAAGTAAAATAATATTACACTGCTGCCACATTTAATCTTCCATGGCCAGACTTATTGTATGCAAACCCAGAATTCTGGACAAC

>Pa7_11936850 Pa7:11936550-11937150

TGTTTGATCTTTTAACAAGAAACATTGCAGAATTCACATACGATTGAGATAAAGGTTGTAATCCCACCAACCTGCAAAATGGTTTGTACAACATTTGAAGAAGTACACGTAATTGTTGGCACAAGCTCATGAGCATATGCTTTTGTCTCTAGAGAAGTGTTAATGTAGACAACATGCAACGAATTAGGAGACCTAGAAGCTGCCTCAAGATAACTCATATAAGAAGGGCTAGATGCAGCATCTGCCAAAGAACAACCTATACGTTCATTTGACATCCTGTAGACGCCAACCTGCAATCA**A(G)**AGATTATGGAAAAGGAATAAATCCATTAACTGCTTCACCAGTATCTGGCACCAAACCAAAATCCAGTTAACTCTACTACAAATAAAGCACACTACATTTAACTCATGCACTAAGATAGATACAGAGCAGTACAGTTGAAGAATCTGCATTGCATTGAACCTTTGAGATATCAAAGCCTTAACAAGTACAGAACACGTTTTGCATTTCTTAAGGACAAATCCATGAACAGAGACAAGGAACTACCTTTTCAAAGCCAGCCTGATCAAGAATAGCCCGGACATTTTCGGACATAAAATCAAC

>Pa7_11969160 Pa7:11968860-11969460

TAAAATTTTCGGGACAAAGATAATACCTTTGCGGCCTGAGGAACATCCCAGCCCAATCGCGCTCTCTTTCGAGGGCGACGATCCAAGTGAGTGAAAGGAAACTCCGTCACGCGCTCAGTCTCCATGGCCGCAGATCGAGAAATCGAAGATTGCTAGAAAGAAAGTAAAATTTTTTTGACGAGAACGGGAAGGAAAAAAGGGATCGGAGAGATGAAAAATAGGGGAAGAAAGGTTGTGTTGTGTGAGAGATCGGCCAATGCGAACCCTAATTTTAACCTAACAACGATGCGTAAGACGATT**C(T)**AGCCGACATCTAACCGCACACGTGTCGCGTTCCTGTCCGTGTGTTTGACCGATCATTGTTATCCAGCTAATCAATGCTTTCCTTATACTATCGTCCAAAAAAAAAAAAAAGCTTTTCTTTTACTATGGTCAGAATTTGGGTTGGGATTTAACCGTGGTTGAGTTAGCTTTATTTTACAGCGAAATATATACCCACGAAAAGAAATTATAGTCCAACGAGGAAAAGACGTCAAAGTTGGGCCTCACCAGTCTTGTTCTTCTTTGAATTGTGGGCTAAAAGTCCAATTAAGATAAGCCAGA

>Pa7_14544359 Pa7:14544059-14544659

CACGCCTTGGTATGCAAATTGCATACCAACCCTTGTTCTATAAATCCCCACGTAGCCACCAACTTTGCTCTTGGATTTATGAACTAACGTCTTTCTTTTCATCAAACACCCATCCAACTTTCCCTCTTCATACTTCCCAAAGCACTCCGAAACGTAGTTGAATCTCCTTTGTCCATCCATCCAAAACTTGGGGATGATTTAATAGATACCTTAGGCTCAGACCTTGCTTGTGATGAAGGCTCCACCTCAAGTAGAACCCCTTTATGAGTTTGTTGTTGATACCCGTCTTCTTTAACCTT**T(A)**ATGTCAAGCTCCACCCACATTGGAGTCCCATCGATGACCACCTTGGTAAGAAGCATCTTTTTGGTTCCCTCCGACCCAACAACATTCTCTCCATCATCCCTTGTCTTGACTTCACTTTTGTGAACCTTATCCTCTAATACCACCACTTCTATACCTTTGAAATCAAGCACCCTTGATGATGAACTGTTATTCATACAACCTTGACTAACCTCCCTTCCCCAACTAGTCTTGAGCATAATTGTCATATTTAACCTTCTCACTATCAAACATTCTTCTATGTATTTTTGATAGCGCAATGAA

>Pa7_14562431 Pa7:14562131-14562731

AAGGATACGTAAACACGTGTACTTAATTAAGGAAAGTAATTATAAAAAAATAATTTTATTTATTTAAAAAGAAAACAAGTGAGAAAGGAAACAAACAAAAGCATAAAAACACTGCGTTCCTTTGTTGAAATTTACAAGACCCAATCACCCGAATGACCAGTAGCAGTTTCCAGTTTCCATCCTAAAGGCTACGTTTTGTGACACCGCGTTTTTCCAATTGCCAAGTCTACCTTTGAATACCCCCTTCCCTCTTTCCCACCCTTTAAAACTCTCTCTCTCCCCCTCTCTCTCCGTAAAAA**G(T)**ATCTGGCTTTTCCTCCTTGCAAGACATTTGATGCAATTTCTCAGGTATGCATATCTTTTTCTTTTAATTGATTTTCTTTGATACTGGTTCTCCTTTTTGATTATTTTCTCCTTTTCCACTTGGGTTTTCTGTAGCTTCTCTTGCTTTGTTCCTTTTTTGTTGTTATCTAACGGTCAACTTCTCTATTTAATTTCAAGAGCCCATTTCTGGCTATCTCTGATTTTCTTTATTCATCTCCTCTGTTTGGTTGCTGAGAAAAGGGAGGAACAAAAAGGAAAACGGAATTTTTGATTCTTTTCT

>Pa7_14903156 Pa7:14902856-14903456

TTTATGTTCTAGAGACCAAATAACCAATTCATATATTTCTGTTTAGAGAAAACCCACCTCGAAATTATAGCAATCTAATTTATTTTGTGAAATTTGTTAACCAACAATTATCCATTTTCACAGATCTGTCAGCAGGCTGAATTGAACCCAGATGCAATTTTTCTTAAAGTTAACTATGAGGAGCTCAAGACTATGTGTCATGCCCTCAACATTCATGTGCTACCATTCTTTAGGTTTTACAAAGGTGCAGAAGGTCGTGTATGTAGCTTCAGCTGCACCAATGCCACTGTAAGTATTTC**C(A)**CTAAATCAATCTTCTTTCTTAAGTTTCAATATCAACGCATTTAGCAGATGTGTTTGTGGATCAAAATACTAAGTTAAAATTCTTATTTGACAGATTAAGAAATTCAAAGATGCCATGGCAAAACATGGGACTGGCCGTTGTAATCTTGGGGGCCCAGCAAAAGGTTTAGATGAATCTGAGATGTTAAAGTTAGATTCAATGGGTGAATTATCAACAACATCAACATCTCCTTCCATGAAGGAAGAAAGAGTGGAGGATCTAGTCACAGAAATTATAGATATGGCTGGTGTTTGGAGCAAT

>Pa7_14944852 Pa7:14944552-14945152

CATAACGAGCTTCATGGAAGAACTTCTCTGCTGCTTGTGCTCCAATTTGGCTTTTAATCAGTGTTTGTGTGCCTTCAAACAGCTCAATTTGCTTGTAGAGTGAGAACCTCTGAATCTGCAATTGACATTTTGATGATCAAATTGCATGCGATCAATCTTAAAGCTAGTATTTTAACATTAAGGTTGCTGCAACTTACAAAGTAGCCACCAGTTTCATTCAAGATTCCACCACCTCCGGAGGCATAGTTTACTCCTTTTTCTAGAATTATATCCTCAGTTAAAGATGGATCCAGAAAGGC**A(G)**GGTGGCCTTGAGAGGCCCATGCTATCACCTAAATAAACAAACATGTTCAGCAGACAACAAAACAAAAAAAGGAAACAAATTAAGCATATACATTAAAGAAAATTGGATTGCAAAAGAAACACTCATGCAGAACAAACTATATGCTTTTTTGGGTTTTTACATATATATGTTACCTATTATATCAGCAACTGTGCGGCCATTAGAGAACCTCCCATTAGGCAGTCCATTGCCAAAATCAATCCCATACCATGGCAGGCTTGCTTGGGCAAGGCTCTTGCTTAGGTACTTGTTGTTTCCGAC

>Pa7_14956008 Pa7:14955708-14956308

TAGTCGGCTTTGCTATCTGATCGAAGAATCGGCTGGAATAGGAGATGCTCTTCAGTGGTGAAAGTGCAACGAGGGACAAGAACTGGAGGCAAATGATGGGTTCCAGAACAAGTGATGTTCGGACGATGTGGGCGTGGATTTGGAGCAAATGTGATCTTCTGGCGCATGATTTGATGAGGTCAAGAAGGGATTGTTTTGGGGTTTGAGTGTGGGAGATGTTGGCGGTAAAGCAAACAGGAACGTTGAGGTGATGGGACTGAGAAGAAGAGAGTGAGCTTAGGTGACGTGAGACTGCTGCT**C(A)**TTCTCATATTTTCGCTCTTTAGTTCCCTTTGCTTATTACAAATATGTGAAATTTAATGTCAACTTTCGAACTACACCATGGCGTCGAAACCTTAACTATTCTTACAATTAGGAATGTACATAAGGCCAGGCCCGATGGGCTACCCAAATTCAGCCCTATGTTTTAGGTTATAGGCTGGCCGGACTCGATACCATGGATATGTTCGGCCAGGCTCATTATGCAAAGGGTAGGGCCACGACTAAGATTTTTAGCCCATGGGCAGCCCGGCATGGTCTGATATTTAATTTAATTTCTTAATAA

>Pa7_14972283 Pa7:14971983-14972583

TCTAAACTGATAATTGCTAAAACAATAGTGTATGATGTAAAGGAAGAAGGGAAGCTCCGCAAAATATAAATAAAAAATAAAAATAAAAATAAATAAATAAATAAAAACCCTGCCTTTTGGAAACAAGAGCCAGCAATAATGACAAGAGCGCATCAGAATTCAGAATAAATGATATGATTATGAAATGGATAATGTCAGTCATAATTCAAACACTTTGTTGCTTCATTGGAGTTGGGATATATTAGCATATCAAATTAGCAATCAGTTTTGGAGACTGCCCTGCATCAAAGGACTAATTT**A(G)**TGCATTTAAATTCCATATATAATGAAATGACTTGTTTGCTCCTCTATATGAGATAGCCCTTTTCCAGGAAATCCTAATGGCCGGATAACTCATTCCACTATCAAATTCTGGGTAATATGAGCTAATCAAAACAAATTTCATCATGCATCCTATCACCAATTGAAAACAAATTGAATATTAAAGACTATATAATTCATGACAAGAGGTAAAAGAAGAAAATAACATACATCTTTTCTGTATATGGCTCCTCGGAAATATAATAGTATAGGACGTTCCTCAAATGAGGCTGATTTGCTGCTG

>Pa7_15008826 Pa7:15008526-15009126

CTAACAAAGTAAAACAATCAGTAAATTTACTTTTTTCCCTCATTCGATGGGGGTTGGGAAGCATGATGTGTTTCAAGTAACTATATGTAGTTAAGAAGGTCGAGTACCTTAACACATGCATGATGGAACAAGATGTGTACATCATGCTAAGAATGCTCTAAATAGAATGCAGATAAACAGTAGTGGTATCTTCATAATATCATTAACCAAAGAACAGTAGTTACTCATCAACCTGAGCCGCTTATAGGACCATCAGGTGCATTGATGAAGTCAACCAGAAATTTCTCCATGACATCAGC**A(G)**GTGTAGTCCATCTTTGGCCTATCAGAGTACCCCAGACCTGGCCAATCAACAATGGTTGCTCGCCAATTAATATTACCATCTTGCTGAACAAGATTACTTGCTACTGATCTCCATTCTTCGACAGTGCTTACATCAGAAATGGTTGGTATCATAAGGATGTTCTTAGAAGGACCCTCTTTCTCCTTTTCATGCTCCTCATAGTAGATGTTAATAGAATTTTCCTTGAATTTCCATTGCCAACTATTGCTCTGAAACAAAGTACAATCATTTGTCAGGCTGACAACACATAATACTGGACTC

>Pa7_15017649 Pa7:15017349-15017949

GAAAACAAAAGGCATCCCCTTTGAGTCTGTGGATGAAGATCTGAATAACAAGAGCCCATTGCTCCTCAAGTACAATCCCGTCCACAAAAAAGTTCCTGTGCTTGTCCACAATGGAAAGCCTATTGTTGAGTCACTCATCATTCTTGAGTACATTGATGAGATATGGAAAACTGGTCCTGGACTTCTGCCTGAGGATCCTTACGAAAGGGCCCGAGTTCGCTTCTGGGCTAGCTTTGTCCACCAACAGGTTTCTAAGTTTTGTCAAACAAATTAATGAGATTTTTTCGGCTAAAATTTTC**G(A)**TGGATTAATAATGGTTACAAGTTTTGAGTGCAGGTATTTGAAAGTCTGTCCTTGGCCTGCACATCTGATGGAGAAGTACAAGAGAAAGCCATCAAGGAATTGCTTGAAAAATTAAAGACATTTGAAGAGGGAATGAAGGAATTTTTCTCAGATGGCATTGCTTCGATTGAAAGAAGCAAGACTTTGGGACTTCTGGACATTGTATTGTGTTCAGTATTTGGACCTCATAAAGTTCAAGAAGAAGTTCTTGGTATCAAAATTGTAGATCCAGAGAAGACCCCACTAATATTTTCTTGGGTG

>Pa7_15101968 Pa7:15101668-15102268

ATTTAGAGAGATTGAGAAAATTATAGCTTGAAATGTAAGAGAATAAAGGAATGAGACCATCACCACCAAAGGATTGAGGATTTAGACAGATTGAGAAAATTGCATGAATAAGTATAGTATTTAATTGGGTATTATATTTATTGTTATTATTATTTATTATATTGATAGGCGGGGTGGTCTGGGCATAGACGAGCTTAATCAGGTCGGGCCTAGGAAAACTCAAATGGGTTGAGCTGCCGGTTGACTCAAGGTTTCAAACCCTTGGCCCAAACCTATCCGATCTTAAAGCAAGTTAAGCT**G(A)**GGCAAGAGTAAGCAACAAGTCGGGTCTGGCTTTCTCCTATCAGCTTTGGCCCATTTGCTGCGGGCCAAATAATGATCCCTAAATATTCTTTATAATTGTTGACCCCCTTTTGTTTTTCCTTTGGAGAATTTTCTTATTCTATGTGCAGTCGTAGTCTACCTCAATGGAAAAGGACTTTGATTTGCATACCAATAGTGGTCTCATGGAAAAACTCATGCACATGATGGCTTAAACCCAGGATCACTTAGAAGCACACCAAAGGTATGCGCCAATCCAACAAAGGGCAAAATCAAAAAAATT

>Pa7_15196176 Pa7:15195876-15196476

ACAATGCCACTGAAAGCAGCAATTACTAACCAATCAAACCTCAAAAACATAAGCTTGGAGAGCAACAAAGAACAAAACTGCAATCCTAGTTTGACCGTTCGATGAAATTCCCAACCATAAAAGAGACATAAGCAAACTCAAAAGCAACAATACAACATTCATCATGAAAAGCCCAGAACAAAAAAAAAACAAACCTTTTTCATTGCAGTCTATCTATTAACCAAACTAAAGGGATCAAATTCATGCATAAACAGATGAGATGGTAACAAAATGCGTAAAGTCCAACCATTTCTATCACAA**C(T)**ACCGAATAAAAACTCCAACTTTTGTATTAGTCCATTTTCACAGAACTCTAAAAGCTAAAACACACAATTCTACTCAATGTTCATAATACCACCACAAAAAAAAAAAAAAAAAAAAGATTAAATCCTTAAAAAAAAAAATGAAATTAAGGGAGGAGGAGGAGGAAGGAGAGCAAACCCAATTCATTTCTGGTAGCCTCCGCCTCGGGCGTTGCAGTCTTCTTCAATAACATCGGTCAAGTAAACCGGAGGGAAATCCGAGACGTCGAAGGTCTTCTTCTCCTTCATTAGCTTCTCGACAT

>Pa7_15909400 Pa7:15909100-15909700

ACGCATAAAAAACAAGTGGCGGGAAGGTATTAGAGAGTTCATGTGAAAAATGTGTAAAAAAAAACATTGATGAGGAAAAACAAAGGTTCACACTCACAGTCACACATTGAGTTTGGCCATTGGCCGGTCGAAACTTCCCGCGTGTTAGAGCATGACTGACTTTTGTGAGTCTAGGTCGACCAAAAAATGAACGGCAAATGCATACCCCAACCCATCAGACCCACAATTCAAAATGACGAAAGTGCCCCGAGTCTATTTTTCATATTATAAATACGAGTGAAACTCTCATCGTAGTCATT**C(T)**ATAGGAATCGGGCAAGACGGTGCGGTGCAGGCTACATTTCTATATGCAGCAGAAAGGCAGAGCTCACGACCGAAGATCAAGGAGCTTTTCCAGATCACGCATTGCTGTTGAGGGTTCTTAGCCCCATTTATTCTTCACATTTCACTCTCTCTTTCTCTCTCATTCGAATTTTCAGCAGCTCAGTCTCTCTCACTCCCATTTCGGTCTACAATGGCTGCTGCTAATGGTATGAATATTACGGGGGCATTTTCGTTGTTTTTACTTCATCCTGAGGCTAATTCTGTATGTGATTCAGATCTC

**Genomic sequences of QTL-derived markers for primer design**

>Marker313919 Pa7:11324765-11325430

GAAATTGGCATATCATTATTGAGAATCAATAATATGATCAAGTTTAAGGAAACACAACAACTAATCAACATGAAACCTTACTGTTTGTCAGCTAGAGATCCCACAACTGTCCCAAACAACATGGATGACCCAAACCCAGCAATAAAAAGCTGTCCAATATCCCCCTTGCTGAACCCATACTGGCTGTAGAGGTAGTACACATACGGACCCTGCAACCAATCCCCGGCTATAGCCACAACCAATTTAATCAGATCCAGAATGATAAAACATTTCAAGGATGATTCATAATGAAAAGCAACAATCTACAATTCCAAGCAGTTTTCAAATAACATAATACTCTTGACCTTAAAGATATATATTACCATCCATAACCAATGCAATCAAGTGTAGCACTACAATCAAAAGACTCAGAC**G(A)**CCATAGCAAAGAGCAGTACATCAATTACAGTACTGATACCATCAACCAACGTAAACAGATAAGGGTGAAGCAACAAGAAATAATTAGAAGGGGTTATAATGACTGGAATATAACTAGATCAATAATCTTGAACAAACAACATGCTTGCTCATTACATAAAATTGCCAATCCAAGAAACTGTATATAGTTGAATCTCTAATTTTAGTCCACTTCGATCTGAATATCCAAATCCAAAAGAAGAGCGACATTGC

>Marker334902 Pa7:11386218-11386863

CAGCAAGCAATGGAGGCACCAAAGGCAATCACAGGCCACACCCGCCTTGGAGCCACCCTTCTCATCACATATTGTGTTGCTTGTTCTCCCTTTGGTATAGGGTAATATGGCTGCCACGTCATAGGTTCAAAAAATAGGGATAACGCCAATTAAACTTGGCACAAACTTGTGGACAAATAATGCCCCCAATAAATGTACACTTCTACAGATGTGACCATATGATATAATGACAATTTTTGTGCTTATGTTCAAAATTACGATCTTTTAAAAACGACATGGTTTTAAATATGATTAAGCATAACATCATGACAAAAAAAACACAGAAATGGAAAACATTGTTTTCAAAAATTTGGGTAAGGGTC**G(T)**TATATAATCAAGAGGTTGAACTTACAGGTGCAAGTAAGGTGAGGGACTTAATGGAGAGAGGGTGTTTAACAGCAAGAGCAAGGGCCAAAATGCAACCCAAAGAATGAGCCACAATGTGGAAGGATTTAACTTTGTTGGGTTCAAGCACAGATCTTTCTATCATCTCCAAATGCTCTCTGAGGGTGTACATGGAGTCAGTTGGCTTTGGGCTCCTCCCAAACCCCAACAGATCAATAGCAAAAAGCCTAAAATTTGATTTTGCAGCACTTGAAAAGTTGGGGA

>Marker339371 Pa7:11068429-11069047

TACCCGGGGTTGCTCATGTCATGCCTAGTAGCTTTCATACGCTGCAAAATACTAGGAGTCGGGATTATCTAGGCCTCTCTTCTCATTCTCCCACAAATCTTATGCATGACACAAGGATGGGAGATGGGATTGTCATTGGTATAATTGGCTCAGGAATATGGCCAGAGTAAAAAATGTTAAATAATGAGGGGGTTGGGCCAATCCCAACATGTTGGAAGGGTCGGTGCTAATCAGGAGAACGCTTCAATGC**C(A)**ACAACAGACTGCAACAGAAAACTTATAGGAGCAAAGTGGTTCAAAGCTGGTTTCCTTGCTGATAATGAACGACCATTCAACACCACTGAATTCCCAGAGTTCTTGTGTCCTAGAGATGCAATGGGACATGGCACACACACTGCCACAACCTCTGCTGGTTCCTTTGTGGCCAATGTAAGTTACAAAGGCCTCATCCTAGGAGTAGTTAGAGGTGGTGCACCCCGGGCTCATTTGGCCGTGTACAGGCCATGTTGGTGCTGCGGCTGAATCAGCTGAAAGCTTTCAATGAGGCTATTTATGATGGGGTTGATGTGTTGTCACCGTCTATGGGAAACTACCTTTCTAAGTTTGCAGAGTCTCATGCAAT

>Marker359157 Pa7:11255628-11256253

CATTTCCAAGTTTATTAGCGGCGGTGCTGATATCATCGCAGGTAAACAAACATATTTTCTCCTAATCATTTTTTTCCCTTGAGGTTTTCCAACTAATGTTTTCCAACCACATAGTAAATCACATCATTAGCAATTTTCTTTCGCTTAAAAAGTGTGACCTTAATAATACTACATTGCTGATCATATCTCT**T(G)**ATAGTGTACGGTGTTATGTAAATGTGGTTCTAATTAGTGTTAATTTTATATGACACGGATTGTGATTCGTAAATATGGTACGAAATTGATATTATTGATGATGGAATATAGGGATGAAATAGATGGTTACAGAAAAGTTTTGGGGTTACTTTTTGCGAGGAAGAGACATGTTTCATGTGAGGCTGTTGCTCTTTTCCTCGTGATTCCTTTCTGGCAATACTTCGATAGCAATCAGGCCAATATGTTTCTGTTCGTTTTTGTTTGCTTGTGTTTTTTCTTTTTCTTTTTCAGGTACAAAGTTAAAATGTTTGTATGCGTGTTTTGTTAAGGACTGTGTTTGTTTGGTTTCGTTTTCCCGTCTGCACCCAACTTCGTGGCTTAGCATGGGTGGAGTGAAGGAGTTTGAGTATGAATTGTATTCTTCCTACTCCAAG

>Marker398293 Pa7:10944201-10944886

TTCATTGGAATTGCAGTTTGTTTGGTTGCATTGTTCATGCATTGTACGGACTGAAGATCCAATTTTAACATTGGATAGGCTAAGCTTCAGTAAACCCCCAATTTACTCTGATTGGAATTCAAAAATTTTGTTGAGTGGAATTGAGTTGAGTTTATGCATATGCATCATTGGATTGGAAGGACAAATAATGATTGGTGCACTATTTTGAGAAAACAAATTAATCAATGGATAGGAC**G(A)**TTGTCAAGTTGATTTAGAATTATGGGTTTGTGTTTTCATAATCTCCTAATTGGATACTTGATATATGAGATTTTTATCCCTCTCCCATTGTAGGATAGCAGTACATGCCTAGTAAATATGCTCACAAGGCAGTATGATTAGTAAATTATATGTTCAGGGCAGTGTATAAAATGTATCATATTTGGCTTAAGGGACGTGGTCCCCCTCTATTTCTAATGTCAGTCTATAGGAAACTTCTACTGGGTTTTGTTCACAGTATTGAGTAGCTCATTTTAATGCTGTACTGCTCTCTCACATGATCAAGTAAGAACACTCACGTGTTCTTATTCAACATGGCATGTGCTGGTAATTGTAATTGTAGGTGAGTCGGTGACATAGCCTTATAGATCCCACCATATTCAGTTCATTGTTTCTGTAAGTGAAATAACTTCTGCATAGATTTGTATAGA

>Marker436423 Pa7:10540778-10541406

ACTCTGTTCTATTTAGTCCGACTGCAAGACTAGGAATTTAGGATCTACCTTTACTTAAACTCTAATTAATCTCTCTCTCTCAACATTAAGCAAACCCAAGAAAGAAAAAAAAGAAAGCAAAAGCAAAAAGCTCCTCCCTTCCACTGCTCCCACCTTCCCTCCCTTTTCTTCAAGGCTCCTACAGCCTCTCCACAGTGTACACCACTGCCAACCCCAATTATCTCTACCTACTCCAACTCATCAAATCAACCTTCAGCTTTCTGCTTGCTAGCTAATCAAGCTTGCAGCTTTATGAATTTATGACCAGCTAACACCACATAAAGAAACCCAAAACTCCTACAAAAACAAAAACGGATGCATAATGACAGCTGAAGAGATATTAATTGTTAAAAATCAATTCTTTTTTACACTTCCATTGATCAAATGGGTTAA**C(T)**AAATTGGGCACCCAAGTTACCAATTTTGGCAAAAACTAAAAAAGCTACACCCTTTTTCACTCATCAGAAGAAAGAAAACCCAAAACTAACAGCTAACAATTAAAACCCAACACAGATCCATCATTCACAGAAATCACAACTCTACACCCAGATTTAGCTTCCACATGGTGAGTGAAGAAGTGCATGACCCGAATC

>Marker437413 Pa7:11037571-11038196

CCATCGTAACACTTTGTGAGGACTTTGTTTTGTGTCCAAGAGGTGGGCGTTGGTTGATCCCACCTCTGCATTATCTGAAATGTGCTTCAGGAAAGCCCATAGCTGATGCAGATGGGTCAAATGCCTGCCTAAGTAAAATTTGCAGGAGTTGTGAGCTTTTTTCAGCAGTCAGCATAGCATTTTTTGTATAATAAGTTCACTCGTAATCTGATTTTATGCATTTCTAAATCTTAAGCATTGTTCCCTTATTAGAGCTGTCGCGG**C(T)**TGAAATTTGTGAAGAGCTTTTTACTCCCACTCCTCCTCACGCCGAGCTTGCATTGAATGGGGTTGAAGTGTTTATGAATGCAAGTGGAAGTCATCACCAACTAAGAAAGCTTGATATTCGTCTTCGTGCTTTTATAGGTGCTACTCACACTCGTGGAGGGGTGTACATGTACAGCAATCACCAGGGGTGTGATGGTGGTCGCCTATACTATGGTACGTGACATTATTTATTCTTCAGTGGTTCTATGCAAATTTTCTTAGGATAATATACTTATCAGTGCTCCATATTTTAAGTTAAGGACAAAATACTGTTTCGTTGGTGGTATTTTGGGTCTATCTCTTATGGAATCTCTTTTAGTGTT

>Marker442696 Pa7:11066424-11067046

ACAGCCTTTATTTTGAAAGGTTGTTATTGTTTTATCTATACTTCTAAATGTAAGTATCTAATTCCATTTCAATTTTTTTTTTGGTTATGAAGCAATAACTAAATTTTGATTTTTTTTAAGCCTCAGAATTTCATTGATCTTGTTTGATCTTAGATCCAATGAAAGAAAGAAGAGAGTGGAAAGCAGACAAAGCAAGGCCTTGAAGAAACAA**G(A)**AAGGGGGAGACCAAGCATGATAGATAAACCATTCATATAGTTGGTTCTACAGTTGAATGGATATATTTTCTACATTAAAAATACATGATAGATGAACCAAGTATATAGTTGCCTATAATTGTGGCAACAGTTTTAAGAGATGCTTAATATTATATTTTCTTGGAAGTAGAGATGCATTTGCTAAATACAATCTGGAAAGAATTGGATTTGTGGACTGCCTATCAGACTTCGCTTTGTGTATGTACCTGTGGGAGCAAAAATTAAGGCCCAATTAACAAGCCACTTCCCATGAACCCTAAATTTAGGGAGTGGAGGGAATCAAATAACATGATATCCACTACCATTGAGCATGGAGATAAATAATGGATATGGGACTTGGAGTCCAAGGGTTCAATGCCTATAAAAGGCTA
