# Supplementary material for: Identification of the PmWEEP locus controlling weeping traits in Prunus mume through an integrated genome-wide association study and quantitative trait locus mapping
Source: Hortic Res. 2021 Jun 1;8:131. doi: 10.1038/s41438-021-00573-4 (PMC8167129; doi:10.1038/s41438-021-00573-4)
Supplement: Supplementary file 3 — Supporting Appendix2 [file 41438_2021_573_MOESM3_ESM.docx]

**Appendix S2** Protein sequence of Pm024213 and the conserved domains identified using the Phytozome database. **(a)** A putative thioredoxin (Trx) domain was searched in *Malus domestica.* **(b)** Protein sequence. The grey shading indicates the thioredoxin domain contained in Pm024213 protein.

**
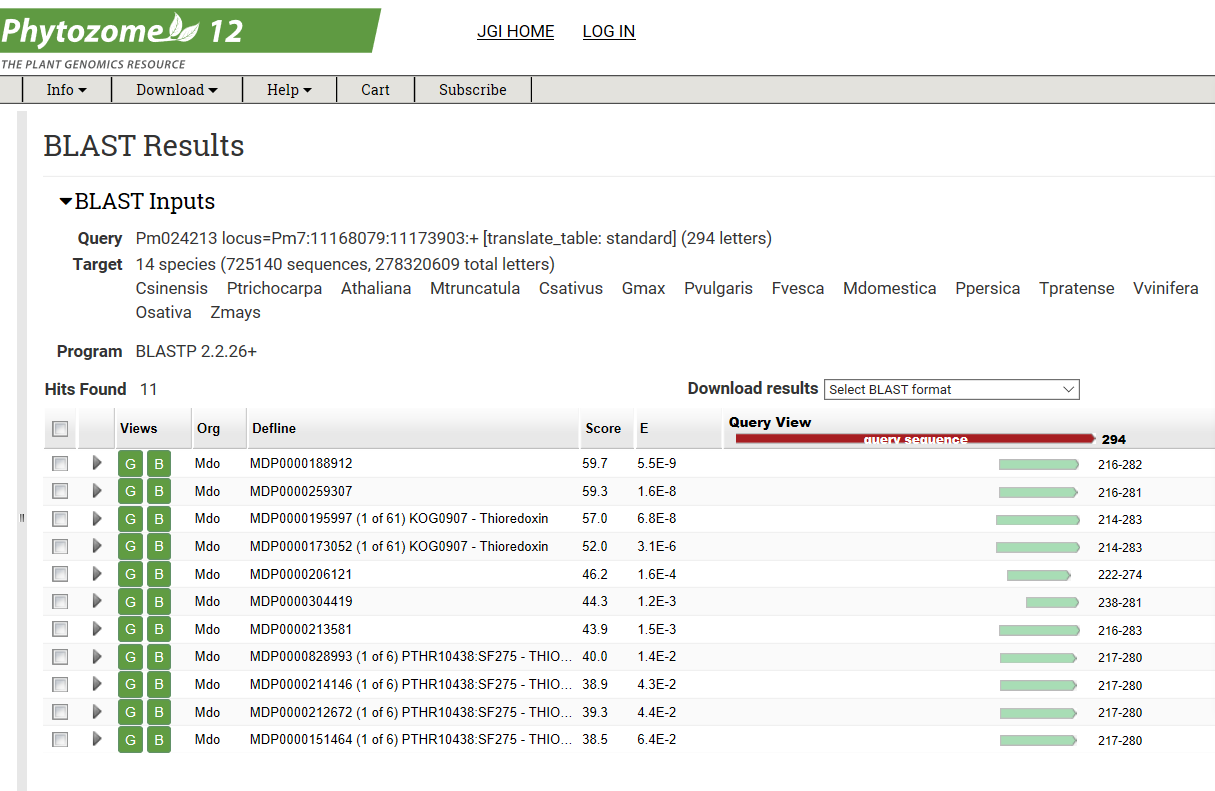
**

(a)

**
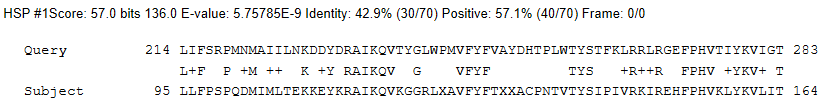
**

(b)

**>Pm024213 locus=Pm7:11168079:11173903:+ [translate_table: standard]**

**MRRKSWRGLWAASLQLPPLPNNDPHASLKRERERERERERVRGRRGWGEWVGVRFGVGVGAWGIRNRRGFLGLWLLVGWGLWVCGRGVVRNDPKCPLDIFGIPYKGNDPILVFCFYQKVEDVVVVIVMVADKKVVVAVAVGGGGDGGSIVVVVEVVMVGKMEKDVVMVEVVVVMKVEVEDVVVVTVMVVEKKVVVAVAVGGGGDGGVGCGGGGLIFSRPmNMAIILNKDDYDRAIKQVTYGLWPmVFYFVAYDHTPLWTYSTFKLRRLRGEFPHVTIYKVIGTPMIKVRTTYGC**
