# Supplementary material for: Identification of the PmWEEP locus controlling weeping traits in Prunus mume through an integrated genome-wide association study and quantitative trait locus mapping
Source: Hortic Res. 2021 Jun 1;8:131. doi: 10.1038/s41438-021-00573-4 (PMC8167129; doi:10.1038/s41438-021-00573-4)
Supplement: Supplementary file 4 — Supporting Appendix3 [file 41438_2021_573_MOESM4_ESM.docx]

**Appendix S3** CDS sequences for primers designed for RT-qPCR.

>Pm024213 locus=Pa7:11168079:11173903:+

ATGAGAAGAAAATCATGGCGTGGATTATGGGCTGCAAGTCTGCAATTGCCGCCACTGCCAAATAACGACCCCCATGCGAGCCTGAAGAGAGAGAGAGAGAGAGAGAGAGAGAGAGAGCGAGTGAGGGGCAGGCGAGGCTGGGGTGAGTGGGTGGGTGTTAGATTCGGGGTTGGGGTGGGCGCCTGGGGAATAAGAAATCGAAGGGGGTTTCTGGGTTTGTGGTTGCTGGTTGGCTGGGGTTTGTGGGTTTGTGGGAGAGGGGTAGTAAGGAATGACCCTAAATGCCCATTGGACATATTTGGAATTCCTTACAAGGGTAATGATCCGATCCTTGTATTTTGTTTTTATCAGAAGGTTGAGGATGTGGTGGTTGTGATAGTGATGGTGGCAGATAAGAAAGTGGTAGTGGCAGTGGCGGTCGGTGGTGGTGGAGATGGTGGCAGCATAGTGGTGGTGGTGGAGGTGGTGATGGTGGGGAAGATGGAGAAGGATGTGGTGATGGTTGAGGTGGTAGTAGTGATGAAGGTTGAGGTTGAGGATGTGGTGGTTGTGACAGTGATGGTGGTAGAGAAGAAAGTGGTGGTGGCAGTGGCGGTCGGTGGTGGTGGAGATGGTGGAGTTGGATGTGGAGGTGGAGGTCTTATATTTTCAAGGCCCGCAAACATGGCGATCATTTTGAACAAGGATGACTATGACAGGG

CAATTAAACAAGTTACATATGGACTTTGGCCAGCGGTTTTCTACTTTGTTGCTTATGACCACACACCTTTGTGGACTTATTCAACTTTTAAGCTTCGTAGGCTGAGGGGGGAATTCCCACATGTAACCATATATAAGGTTATCGGTACACCTATGATTAAGGTGAGAACAACATATGGATGTTAA

>Pm005182 locus=Pa2:9685220:9690217:+

ATGGACAGAAACCAATTGCAACGCCAGGTTGCCCTCAAGAGAAGCTCTCTGTTTGAGCAGCAGTTCCTGGATTCACAGCAATTCGTACAGCTGGAGGATTTGGAAGACGATGCTAACCCAAATTTTGTGGAGGAAATTGTGACATTGTTCTACAAAGACTCAGCTAGACTGTTCCAAAAAATTGAACAAACCATGCAGAGTAGATCTATTGATTTTGGCAAGCTGGATGATTACATGCACCAGTTCAAGGGTAGTAGCTCAAGCATTGGAGCTATAAAGGTGAAGAATGAATGCTCCCAATTTAAGGAATTTTGTTCGGCAGGAAATGCTGAAGGCTGCTTCTGGGCTTTCCAGAGAGTGAAACAAGAGCACCACACCTTGAGGATCCAACTTGAATCTTACTTTCAGAGGCTTTTGTTGCATGCTTCGGAAATGGGTCAGTTGTATAATAAGAGAAGCTATGTTGCATCTGAAGACAAAAAGCTGGAGCTGAGGCTTGGTCCTCCAGGAGAAGACCAGTCTCTTCTCTCTCTTGGTTGCATCATCATCAACAACAACATTTCTCATGAACCCAAAAGAGTCTGCCATGAAACTTTTAAAGAGAAAAAAGAAGAGAGAGAGAGCAAGTGGTTGACAAACTCTGCTCCCAGCAGTCAGTGCCACCAAAACCCTAAGCCTTCCCATTTTCAGTGTCCAATGATTTCCAAACCCTGCACCCCTGGAGTGGCAGAGTTCCACAACTCAGATAAGAAGGCATGTTCAGATCCTGCTATTGCTTCAGAGTTTACAAATCCTGCTGCAGTTCATGGCTCTGATCAGAAAAGTAAAACCAGAATTGCACAAGCTGCAGTTGTAGGGTGGCCTCCAGTCCGTTCATCCAGGAAAAATCTTGCAAGCAGCAAATCAAGCTTTTCGAAGCCCCCGAATTCTGAGTCACCAAATGAAATTCTGCAGGACGGGAGCAGTGGAAAATCTGATACTAATTCCAAGCCTCATATGT

TTGTAAAGATCAACATGGAAGGGGTTCCCATTGGAAGAAAAATCAACCTCAAAGCCTATGACAGTTATGAGAAACTCTCCCTTGCCATAGATGAACTCTTCCAAGGTCTTCTTGCAGCCCAAAGGGTTTGTTCTGATGTGGAAAAAGAAGACAAGAAGGGAGAGACCAAATCAATAACTCATGGCAATGGGGAATATACTCTGCTCTATGAGGATCATGAAGGAGACAGGATGCTTGTTGGTGATGTCCCATGGAATATGTTTGTATCCACTGCAAAAAGACTGCGCGTGCTGAAGAGCTCAGAGCTTTCCACTCTAAAACTTAGCAGCAGTCAACATGAAAAGACACCACTTGATACTCCAATGGTAGTTGGGAAATGA

>Pm011163 locus=Pa3:9358924:9360357:+

ATGGTGTTTGTGACTAAACTACCTACTGAAAACTTTTCTTATCCTAGAGGCAGCAAAGTCTCCAAATTCTTTGCTGGGATTCCTCTAATAGACCTTTCAAAACCAGACTCCAAACAGCTCATCGTCAGGGCTTGTGAGGAGTTTGGGTTCTTCAAGATCATCAACCATGGTGTTCCAATGGAATTCATTACCAGGTTGGAATCTGAGGCCATCAAATTCTTCTCCTTGCCACTTTCTGAGAAAGAAAAAGCAGGGCAACCTAATCCATTCGGATATGGTAACAAGTATATTGGGAAGAACGGTGATGTTGGTTGGGTGGAGTACCTTCTTCTAACAGCCAACACAGAATCCAACTCCCAGAGATTTTTATCAGTTTTTGGACAGAACCCAGAAGAGTTTTGTTCTGCTTTGAATGATTATATATCAGCTGTGAAGAAAATGACATGTGAGATTCTTGAACTGATGGCTGAAGGATTAAAGATTCAACCGAGGAATGTGCTCAGCAAGCTTTTGATGGATGAACAGAGTGACTCTTACTTCAGGTTAAATCACTACCCACCATGCCCAGAGCTTCAAGATTTGAGTGCCAGAAATGTGATTGGATTTGGAGCGCACACAGACCCACAAATCATCTCTGTGCTGAGATCCAACAACACATCTGGCCTCCAAATTTCATTGAAAGATGGGAATTGGATTCCAGTCCCACCTGATCATAACTCCTTCTTCATCAATGTTGGTGACTCTTTGCAGGTTTTGACCAATGGGAGGTTCCAAAGTGTGAGGCACAGGGTTTTGGCCAATGGTTCAAAATCAAGAGTTTCAATGATTTATTTTGGGGGGCCACCCTTGAGTGAGAAAATAGCTCCACTGCAATCTGTCATGAAAGGAGAGGAGGAAAGCATGTACAAAGAGTTTACATGGGTTGAGTACAAAACCTCTTGTTACAACTCAAGACTGGCTGATAATAGGCTTGGAAACTTTGAGAGAATTGCAGCCTCATAA

>Pm012630 locus=Pa3:21656184:21657564:-

ATGGGAAGTGGAAGTGAGTTGGAGTTACCGCCAGGGTTCAGATTTCACCCAACGGATGAGGAGTTGGTCAATTATTACTTGTGCCGGAAATGTGCTGGGCAGCCTCTTGCTGTTCCCATCATCAAAGAGATTGATCTTTACAAGTTTGATCCTTGGCAGCTACCTGAATTGGCTCTTTATGGAGAAAAAGAGTGGTATTTCTTTTCGCCAAGGGATAGAAAATATCCGAACGGTTCAAGGCCGAACAGGGCAGCAGGAACCGGGTACTGGAAGGCGACCGGGGCGGACAAGCACATTGGAAAGCCCAAGGCACTTGGGATTAAAAAGGCACTCGTGTTCTACGCGGGCAAAGCTCCTAGAGGAGTTAAAACCAATTGGATCATGCACGAGTACCGCCTTGCAAATGTCGATAGGTCGGCTTCCAAGAAAAACAACAACTTGAGGCTTGATGATTGGGTGCTATGCCGAATATACAACAAGAAAGGTAGCATAGAGAAACATAATGTTGCCATGGAGCGCAGTAAAATGACCAAATACCCAGAAATATTGCATGAGCAAAAACCGGAAATGACCCAAATGCCACCACCCCATACGGATATGTCGTCGATGGATTCAGCACCGAGGGTGCAGCAGACGACGGACTACTCTAGCTGCTCGGAGCACGTGCTGTCGCCAGAGGTCACGTGGGAGAAGGAGGTCCAAAGTGAGCTACAATGGAGCAGTGATGAATTAGAGAATTCCCTTAATACCCTTGATAATCAGTTCATCAATTACATGGATGGCTTCTCAGATATTCTTGACCCTTTTGGCGCTGCTCAGCCTCAGTACCAAATGGACCAGCAGAACATGTTTGCCTACTTACAAACCCAATTTTAA

>Pm012998 locus=Pa4:1017340:1019721:-

ATGGTGGGTGGATTAATGCTCTCTCCAGATATGGATGCTGCTCAAGATCAATATGATGGTAGTCGTTCAGAAAGTGTTCTCTCAGCAAACAATGGGGTTTCATTTAGTGTTTCTGGGCTTCAGTTGAAGGATCAGTTCTCTAATGGAAATGACTCTGCTCCCAAGGCGAGAAAACCATACACAATCACAAAACAAAGAGAGAGATGGACAGAGGAAGAGCATAAGAAATTCCTTGAAGCTTTAAAGCTGTATGGTCGAGCCTGGCGAAAGATAGAAGAGCATGTTGGCACCAAGACTGCAGTTCAGATTCGAAGTCATGCTCAAAAGTTGCTCGTGACTCGAATGGCAGCAACCATTGACATCCCTCCTCCTCGACCAAAACGGAAGCCAATGCGTCCTTATCCCCGAAAACTTGTACACCCTGTTAATAAAGAGACCTTTATTGTAGAGCGGCCAACAAGGTCTGCATCTCCAAATTTATCAGTTTCGGAGCCAGAAAACCAGTCTCCAACATCAGTATTATCTGTGATTGGCTCAGATACACTGGGTTCCGCTGATTCAAATACACCCAGTCGTAGTTTATCACCCGTTTCATCTGCTGCTGATGTCCATGGTGTGGACTTAAATCATTCTGAACCCCCCAACCCATCACTGGAGGAAAGTGGATCTACATCACCAGCTGTAGCAGAAAATGGTTCACTTCCTAACATGCAATTATCAATGAAGCTTGAGTTATTTCCCACGGATAATGTTGATGCTAGTGGAGTTTCAGCTGAGGAGGTATCCGCACGAAGCCTTAAACTCTTTGGAAGAACTGTATTGGTCACGGATTCCCACAGACCATCTTCTCCAACCATGGGAACTTCTAAATCACTGCCTTCTGATGTGAAAGAGGAGAAACCTGTGCAGACATCAACACTATGTAACTTTACAGCTACAGAATCTGCATCTGGGAGTGTGGAACATGTTTGGGACAATTTTCCCTATGGAGTACATCCAGGCATGTATTTTATGCAATTTCAGAATCAGAACTCAAATCTGGTAGAATCTGGTTCTGCTTATCCTGTACCTTGGTGGAGTTTATGTCCAAAATTGCCATTTCCTTTTATTCCATTCCATAAGCCACAAGCAGTAAAAGAACATTTTGATGGTAATCTTGGAGATCCTAAGGAAGTTGAGAAGGAAGGGTCTTGGACTGGTTCAGATGCTGGATCAGTCAACGATGAGGAAAGCGGTGACAAATGTCTAGGTATTGAGACTGAGGGTAAAGAACAAGAGCCAAATTCGGTTCTCCAATTTA

AGGCAAGTGCAAACTCGGCCTTCTCTGAATTAAGAGCAAGCCCTAGCCCTGGAAAGTGTAGAAAAGGATTTGTACCGTATAAAAGATGTTTGGCTGAGAGAGACACCTCTACAATAGCAAGTGAAGACAGGGATGGGAAAAGAGTCCGCCTTTCCTTGTAG

>Pm013791 locus=Pa4:6725543:6727134:+

ATGGACTACTCATCTGCTGCATATGATGATACTTCTTTGGATCTTAATACCAAGCCTCTCCGACTTTTCGATGATACTCCGATCAAGAAAGAGGCGCAAAGCAAAATATTGATTGGCTTTGGGAGGCAGCTTTCACCAGATGAAGAGAGTGGTGCTCTATTGGAGGAATTGCAACGGGTGAGTGCAGAGAACAAGAAGCTAACCGAAATGTTGACGGTGATGGGTGAGAGCTACAATGGTTTAAGAAACCAGTTGCTGGATTACATGAGCAAGAACCCAGAGAAGGAGCTTAGCCCAATTTCAAAGAAAAGAAAGTCTGAAAGCAGTAACAACAACAACACCAACAGCAACAATAACATCAATGGAGCAGTGAATGGAAACTCTGAGAGCAGCTCCAGTGATGGAGAATCTTGCAAGAAACCAAGGGAAGAGAACATCAAGGCAAAGATTTCAAGGGCTTATGTTCGTACCGAAGCATCAGATACAACAAGCCTGGTTGTGAAGGATGGATATCAATGGAGAAAGTATGGCCAAAAAGTTACTAGAGATAATCCTTGTCCTAGAGCTTACTTCAAATGCTCTTTTGCTCCAAGCTGCCCTGTCAAAAAGAAGGTGCAGAGAAGTGTTGAAGATCAATCTATTCTGGTGGCAACTTATGAAGGTGAACACAATCATTCCCACCCTTCTCAAATTGAAGCAACATCAGGCTCAAACCGCTGCATGACCTTAGGATCAGTCCCCTGCTCAACCTCCCTTGCCTCATCCGGACCTACCATCACTCTTGACTTGACCAAATCCAAGTCCAGTGCTGACACCAAAAGTACGAAAACAAAAACCGAAACACCGGAAGTTCGAAAGTTTTTGGTGGAGCAGATGGCTTCTTCCTTGACAAAAGATCCCGATTTCACGAAAGCACTAGCAGCAGCCATTTCAGGAAGAATACTTCAACATAATTCTTACTGA

>Pm021243 locus=Pa6:7546969:7548992:+

ATGGCCGTTGACTCTTCCCTTTCTCCACTCGGGCCACCGGCCTGCAAGAAAGACGCCAAGGCGCTGAAATTCATTGAAGAAACAACCCGAAACGCCGACCCGGTGCAAGAGCGGGTGCTAGCTGAGATACTGGCCCGAAACGCCGAGACTGAGTACCTCAAAAGATACAAACTCGGCGGCGCCACCGACCGCGAAACGTTCAAATCGAAACTGCCGATCATTTCCTACGAGGATCTCCAGCCCGAAATCCAACGCATCGCCAATGGAGATCGCTCTCCCATCTTGTCTGCTCACCCAATCTCTGAATTCCTCACAAGTTCTGGGACTTCAGCTGGAGAGAGGAAGTTGATGCCAACAATTCAGGAGGAGTTGGATCGCCGTCAATTATTGTACAGCCTTCTCATGCCCGTCATGAACCTTTGTGTGCCTGGACTGGACAAAGGCAAGGGCCTGTACTTTCTGTTCGTGAAGTCCGAAACCAAGACGCCGGGTGGACTCTT

GGCCCGACCTGTTCTCACCAGCTACTACAAGAGCGAGCACTTCAAGACCCGCCCGTACGACCCGTATAATGTCTACACAAGCCCCAACGAGGCCATTCTTTGCCCCGACTCGTTCCAGAGCATGTACGCCCAAATGCTCTGCGGCCTCCTTGAGCGCAAGCAAGTCCTCCGACTCGGCGCCGTTTTCGCATCGGGTCTTCTCCGGGCCATCCGCTTCCTCCAGCTCAACTGGCAGCAACTGGTGAACGACATCCGAACCGGAACTCTCAACCCGAAAATAACCGAACCGAATTTGAAGTCATGTATGGACGGGATTTTAAAACCCGACCCGGAGCTGGCGGACTTCGTGTCCAAACAATGCGGAGATGAAAACTGGGATGGGATTTTAACCCGGATATGGCCAAACACCAAGTACCTGGACGTGATCGTAACCGGAGCAATGGCTCAATATATTCCGACCTTGGAGTACTACAGCGGCGGCTTGCCGATGGCGTGCACTA

TGTATGCTTCCTCCGAATGCTATTTCGGACTCAATCTCAACCCGATGTGCAAACCATCCGAAGTTTCCTACACCATAATGCCAAACATGGCCTATTTCGAGTTCCTGCCGTACGATCCCAACTCGGCGAGTCGCGGCGACTCGATGACGACTCGACTCGTTGACCTGGTCAACGTTGAGGTCGGGAGAGAGTACGAGCTCGTCATCACCACCTACGCCGGGCTGTACCGGTACAGAGTCGGCGACATCCTCCGAGTCACTGGGTTCCACAACTCAGCGCCGCAGTTCCATTTCGTACGGAGGAAGAACGTGCTGCTCAGCATCGACTCGGACAAGACCGACGAGGCTGAGTTGCAACTCGCCGTCGAGAACGCCTCTAAGCTTCTCCGATCCTTCAACGCCAGCGTCGTTGAGTACACGAGCTACGCCGACACGACGACGATCCCGGGGCACTACGTGATCTACTGGGAGCTTCTGGTGAAGGACTCGGCAAACTCGGAG

ACCGAGTCGGTCGACTCGGTGATGAGTCAGTGCTGCTTGGCAATGGAGGAATCGCTGAACTCGGTTTACCGACAGGGCCGAGTCGCGGACAACTCGATCGGGCCGCTGGAGATACGTGTGGTGAAGAGTGGCACGTTTGAGGACTTGATGGATTACGCAATCTCAAGAGGGGCGTCCATCAACCAGTATAAGGTGCCGAGGTGTGTGAATTTTACCCCGATTATGGAGTTACTGGACTCTAGGGTGGTTTCTAAGCATTTTAGCCCATCTTTACCGCATTGGACCCCAGAAAGGAGACGGTAA

>Pm023083 locus=Pa7:1997951:1999600:-

ATGGCAAATGAGATATGGATCGTTCCATTTTTTGGGCAGGGTCATCTGTTCCCATTAATGGAGCTCTGCAAGCAAATAGCCTCCAGAAACTTCAAAGCCGTTTTCGTCATTTCCTCCAACCTTTCCTCCTCTGTCCCTTCATCTCTCCGCCAATTCCCACTTGTCCAAATCGCTGAAATCCCAGATGAAATCCCACCACCATCGTCGTCTGGTTCATCTCCGCTGCCTCAGCCCAGCTCAGGCCCGCCCCGCCCCCACCATGATGGCCATAATCAGATGGGCGTTGGCCTTGAAAAGCTGCTCTCGACCCGATCCGATAATCCGGATTCTGCAACACCCGTTTGTGCTGTCCTTGACGTCATGGCGGGCTGGAATGCCAAGGTTTTTAAGAAATTTGGAATTCCAACTGTGACCATCTTCACTTCCGGTGCTTGTTCTGCTGCCATGGAGTATGCCATGTGGAAGGCCCAACCCTTGGATATCAAATCCGGGGAGACCCGTTTGCTCCCCGGGCTACCCGAAGAAATGGCCCTCACGCTATCTGATCTGAAACAACATTCTCGGGAGCCACCGCCACCCAATCATGGCGGCCGTCCACCACCAGGGGCAGGTGCAGGTTTTCCTCCACCTGGTCCACCTCCTAGTGGGCATAGCGATGGCCCACCACTAAGCTTAGGTGGAAATTTTCCTCCACATGGCCTGCCTCCACCATCAGGCCCAGGTGTAGATTTTCCTCCACCTGGCGCACCTTTCGATGGACCTGGGCAGCAAAGAAGGGGCCCACCCAAACCAGGCGGTCGACCTCTTTGGGTAGATGAAGCGGACCGCTCTATTGCCTTAATGATCAACACGTGTGATGATTTGGAGCGCCCATTTATAGAATACCTAGCCAAACAAATTGGGAAACCGGTTTGGGGAGTGGGCCCGCTTTTGCCTGAACAATACTGGAAATCAGACGGTTCAATTCTCCGCGACGGCAAACTCCGAACCAGCAGTCGAAGATCCAACATCAGCGAGGACGAGGTGATTGAGTGGTTAGATTCGAAGTCAAATGGATCAGTACTATATGTGTCATTCGGAAGCGAGGTTGGTCCTACTGTGGAGGAGTTCTCAATACTGGCAGAGGCATTAGAAGCATCAAACCGGCCATTCATATGGGTGGTCCAATCCGGTTCAGGTAGACCGCCGGGTCCGCCCCATGCCGGTGCAAAAGCTGAAGAAGGATATTTCCCTCATGGATTGGAAGAACGGGTGGGTAAAAGGGGTCTGATAATACATGGATGGGCACCACAGCTGTTGATACTGAGCCACCCATCAACAGGAGGATTTTTATCGCACTGTGGATGGAATTCAACTGTGGAAGCAATTGGGCGTGGGGTCCCATTTTTGGCGTGGCCAATCAGAGGCGACCAACACCATGATGCCAAATTGGTGGTGAGTTTTCTGAAAGTGGGGTATCCGATTTCTGATGAAATTTCAGAGAAAATCAAGAAGGATGATATAGTGAAGGGAATTGAGAAGCTGATGGGTGATGAAGAAATGAAACAACGAGCTGTTAGGCTTAGTGCCAAGTTTGAGCATGGCTTTCCAACGAGTTCGGTTGCTGCATTAGATGCTTTTAGGGATTTTGTAGCTCAAAAAGCAGCTTAA

>Pm028731 locus=scaffold162:908301:914154:-

ATGGACACACAGTCATCTGGAGAAGATTTGGTCATTAAGGCAAGAAAGCCATATACTATTACCAAGCAACGAGAGCGATGGACAGAGGAGGAGCATAACAGGTTTCTTGATGCCTTGAAGCTCTATGGCCGAGCATGGCAGCGCATTGAAGAACATATTGGAACAAAGACTGCTGTGCAGATCAGAAGTCATGCTCAGAAATTTTTTTCAAAGCTGGAGAAGGAGGCACACGATAAAGGTGTTCCGGCAGGACAATCAATTGACATAGATATTCCGCCTCCACGCCCCAAAAGGAAACCAAGCAATCCTTATCCTCGAAAGTCTTGTTCAGCTGCTTCTACATGGGCCACATCGCATGTGGCAGCAAAGGATGGAAAACTTTTATCATCAACATCATCTTCGCATTGTAAACAAGTAGTGGACTTGGAGAAAGAACCACTTGATGAGAGACCTATCAAAGAAGAAAATCCAAGAAATGGAAAAGAAAATCAGGATGAAAACTGCTCAGAAGTCTTAACTATGCTTCTAGAAGATCATTGTTCCTCTGTTTCTTCTGCAAACAAAAATTCCATACCCACACAGGTGGCACTAAGAAATGCTCGCACTTTTAGGGAGTTTGTGCCTTCCCTGAAAGAGGTAATAAGTCAAGATGTAACAAATGATTCTTATGTCACTACTGAACTTAATGGAAATCAGAATTTGAAGAAAAATGATGCCAAAAAGATAGTTCAAGATAATGGTACAAGTGGAGCCTCAGAGTCAGAGAACACTAATGCTTTTCATAAGAAGTTGGTTCAAGGTGAGAAAGCAGATTATTTGAATTGTGCATTGCCAACAGATGGGATGCAAGGAACTCAGACCTACCCGAGGAATGTTCCTGTACACGTACTGGATGGGAGCCTAGGAGCATGTAATCAAATTACTCCAGCAGATATGTCATTCGCGGATACTGCTTTTCATCCTATGGGCAGGGTTCATGGACAGCCTAACCTTTTTGCAA

ATCCAACTGCATCTACTACTACTGAACATGAAAGTAATGCATCAAGATCTTCTGTTCACCAATCATTTCCAGCTTTTCACCCTCCCTTCACCCCATTACACCATGGTCAAAAGGATTACCAGTCATTTCTGCACATGTCCTCCACATTTTCAAGTCTTATTGTGTCTTCTCTGTTACAAAACCCTGCAGCCCATGCTGCAGCTAGCTTTGCAGCCACATTTTGGCCTTGTGCAAATGCAGAAAATACAGAAGATTCTCCGGCATGCCCCCCGGGAGGTTTTCTATCTAGGCAAATGAACTCCCCTCCAAGTATGGCAGCAATTGCTGCTGCCACCGTAGCTGCTGCATCTGCATGGTGGGCATCCCATGGATTGCTTCCCTTGTGCGCTCCTGTTCAAACTGCTTTTAGCTGTCCTCCTGAGTCCATGACTGGGGTTCCATCAATGGATATTGGTGAAGCTCCTGCAGCCAATATAGAGAGAGGAGAGAATTCTCTTCAAATTCCTTCTTTGCAGGATCAACAAGTGGACCCAGAACACTTGGAAGCTGTGGAAGCTCAAGATCCAGCTTCAAAATCACCAAGTGTGTCATCATCACACTCTGATAACGGAGGTGCAGAACCTAATATTGTACTTAAAGCTGCTGCTGATGAGAAGGTGGTAGCATCAACCGAAGAAGTTAATGATTCAAACAATGCAAAGAGCAGAAAACAGGTTGACCGTTCTTCGTGTGGTTCCAACACACCTTCCAGCAGCGAAGTAGAGACAGATGCATTAGAGAAGCAAGAGAAGGGGAAGGAAGAACTGAAAGAACCTGATCTAAATCACCCAGCTTCCGACTCTACTTATCGTCGCAGTCGAAGTATCATCAACATTAGTGATCCATGGAAGGAGGTTTCCGAAGAGGGGCGTATGGCCTTTCAAGCACTATTCTCGAGGGAGGTATTGCCCCAAAGTTTTTCACCTCCCCCCAAGGACAAAGAGCATCAGACCACTACAAAAGAAGGAAAGCAGAATGTTGAGGACAAAGGTGAAGATGCATCGCTATTAGACCTAAACAAAAAGACATGGGTGCCATTTTCGTGCCACCCGGAAGTGGAGAAAAATGTGTCACCCGTAGGAGACAACAATGCAGAGGGGCTGCTGACAATAGGACTTAGCCAGGGAAAACTTAAGGCTCGTCGAACAGGATTCAAGCCTTACAAAAGGTGCTCGGTAGAGGCCAACGAGAACAGGGCAGCCAATTCCATCAGCCACTGTGAAGAGAAAGGTCCCAAGAGGTTACGCTTGGAAGGGGAAGCTCAAAATTGA

>Pm029452 locus=scaffold265:330024:331790:-

ATGGACTCGCACCAACATTTTGGATTTGGTGTGACTGGTGCAGGATTTTCATATACAGCTCCACATTCCACTGTTCCATCTTTACCTACAAGGTTACTGGGGTCACTGAAATTTGATATAGGGAATTCACCAAATTCACCCTTTTCTACTCACTTTGATTCTGATACCCTTACTACACTGAGTGACGGTCAGGAGCAGCACAGCTCCACAGAGAATCTCTCAGGAGCTAGCCCTTCCTGTAACTCTTCATTTGAAACAAACAGTTACATTCATCAATTAAGCTTCAGTCCTTCTGTGGACTGTCGTCGAGACAGTCTGCAGCTCTATTCTGGCAGGTCTTCTTTATTACAGGATGCAAATTCTAGCCAGAATATAAAGCACGCTTTGCAGGAACTGGAGAGTACTTTAATGGGGCCTGACAATGAAGAAGAAGAAGTCACCACACTGAACACTTCTTTTGGAGAAAGTAGCAAGCAGCAGACACAGAGGTCCACTTCATGGATCCAGGAGATCCAGGAGCACCAGGGTTCACCTGTAGTTCAACGTCAGACATCTTTTGTCTCTAGGCAAAGGCAGTTGCGTGAATTTCAGATTGAGAAACGTTACAAAGTAATAGATGAAGGGTCTCTAAAGGGTTTACCAGCAGGTAATCTGAAGGAATTGCTGATTGCATGTGCTGGAGCTCTCTCTGAGAACAACATTGATAGTTTTGATAAGTTGATTGAAAAGGCTAGAGGGGCTGTGTCTGTCAGTGGAGAACCAATCCAGCGACTTGGAGCTTACTTGGTAGAAGGGCTGGTAGCAAGGAAGGAGGCGTCGGGTGCCAATATTTACCGTGCCCGGAGGTGTAGAGAGCCTGAAAGCGATGACTTGCTCTCATACATGCAGATTCTGTATGAGATCTGCCCCTATTTAAAGTTTGGTTACATGGCAGCCAATGGGGCCATTGCTGAAGCCTGCAGAAATGAGGACCGCATCCACATTATAGACTTCCAGATTGCTCAGGGAACCCAGTGGGTGACTCTCCTTCAAGCACTTGCAGCAAGACCCGGTGGGGCACCCCATGTACGGATTACAGGTATTGATGATCCTCTTTCTCAATATGCCCGTGGTGATGGATTGGAGGCAGTTGGGAGACGGCTGAAAGCTA

TCTCTGAGAAATTTAACATCCCAGTTGAATTTCATGGAGTGCCAGTTTTTGCACCTGATGTCACACAGGACATGCTTGATGTCAGGCATGGAGAGGCTCTTGCTGTGAACTTTCCCCTGCAGCTTCATCACACACCAGATGAGAGTGTTGACGAGAACAATCCGAGGGATGGGCTGCTGAGAATGGTAAAGTCACTATCTCCAAAAGTGACCACTTTGGTGGAGCAAGAGTCAAACACAAACACAACCCCTTTCTTCAATAGGTTTGTGGAAGCTCTAGAATACTACTTGGCAATGTTTGAGTCTATTGACGTGACCCTGCCGAGGAACAACAAGGAGAGGATCAACGTGGAGCAGCATTGTCTGGCAAGAGATATGGTGAACGTCATTGCTTGCGAGGGAAAGGAAAGGGTGGAACGCCATGAGCTCTTTGGCAAGTGGAAGTCCAGGTTGACAATGGCAGGGTTTCAGCAATACCCATTGAGCTCATATGTCAACTCT

GTCATAAGGAGTCTGCTGAGGTGTTATTCGGAGCATTACACGCTGGTGGACAGGGACGGGGCTATGCTGTTGGGATGGAAGGACAGGAACTTGATATCAGCTTCTGCCTGGCATTGA
