# Supplementary material for: Identification of the PmWEEP locus controlling weeping traits in Prunus mume through an integrated genome-wide association study and quantitative trait locus mapping
Source: Hortic Res. 2021 Jun 1;8:131. doi: 10.1038/s41438-021-00573-4 (PMC8167129; doi:10.1038/s41438-021-00573-4)

Figure S1 A diagram of branch dissection for the analysis of specific expression patterns.

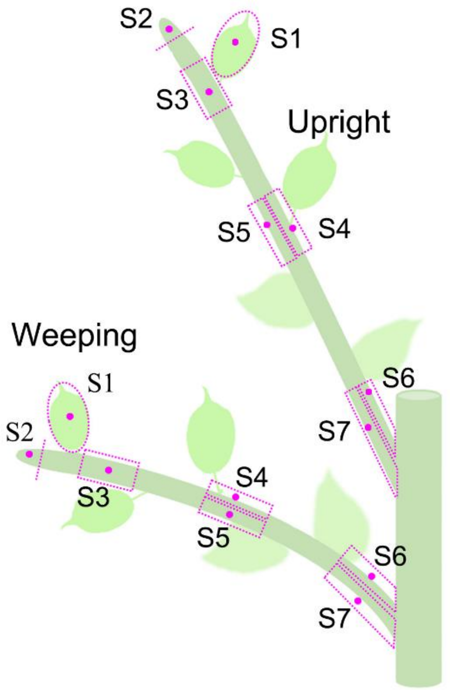

Figure S2 Variations in the tree architecture of *P. mume* in the F1 segregating population derived from ‘Liu-Ban’ (upright) × ‘FentaiChuizhi’ (weeping).

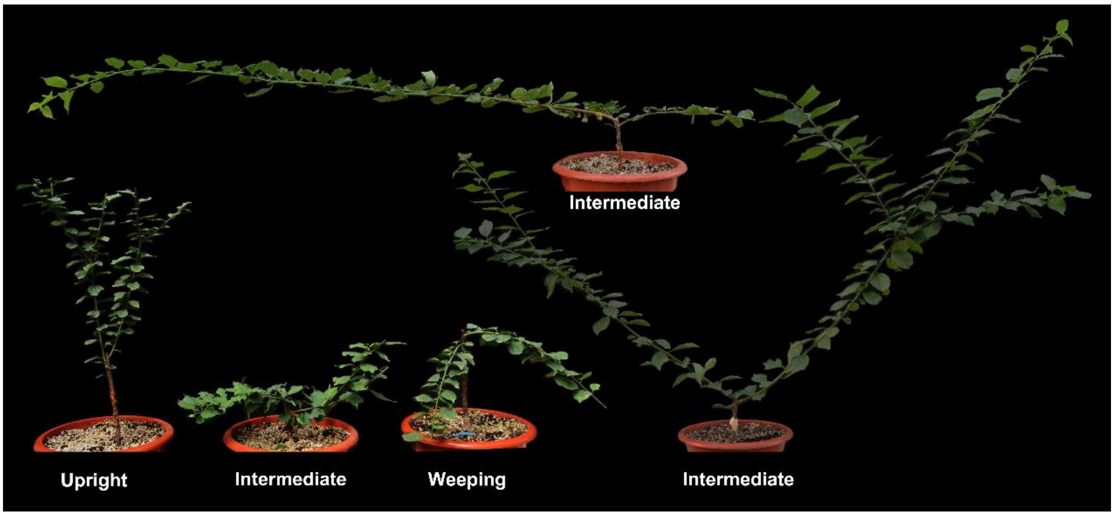

Figure S3 Diagram of the analysis of the weeping trait phenotype using nested phenotyping. **(a)** Branch angle (A1) at growing stage. **(b)** Branch angle (A2) at dormancy stage. **(c)** The branch curved angle from T1 to T5 sections were measured.

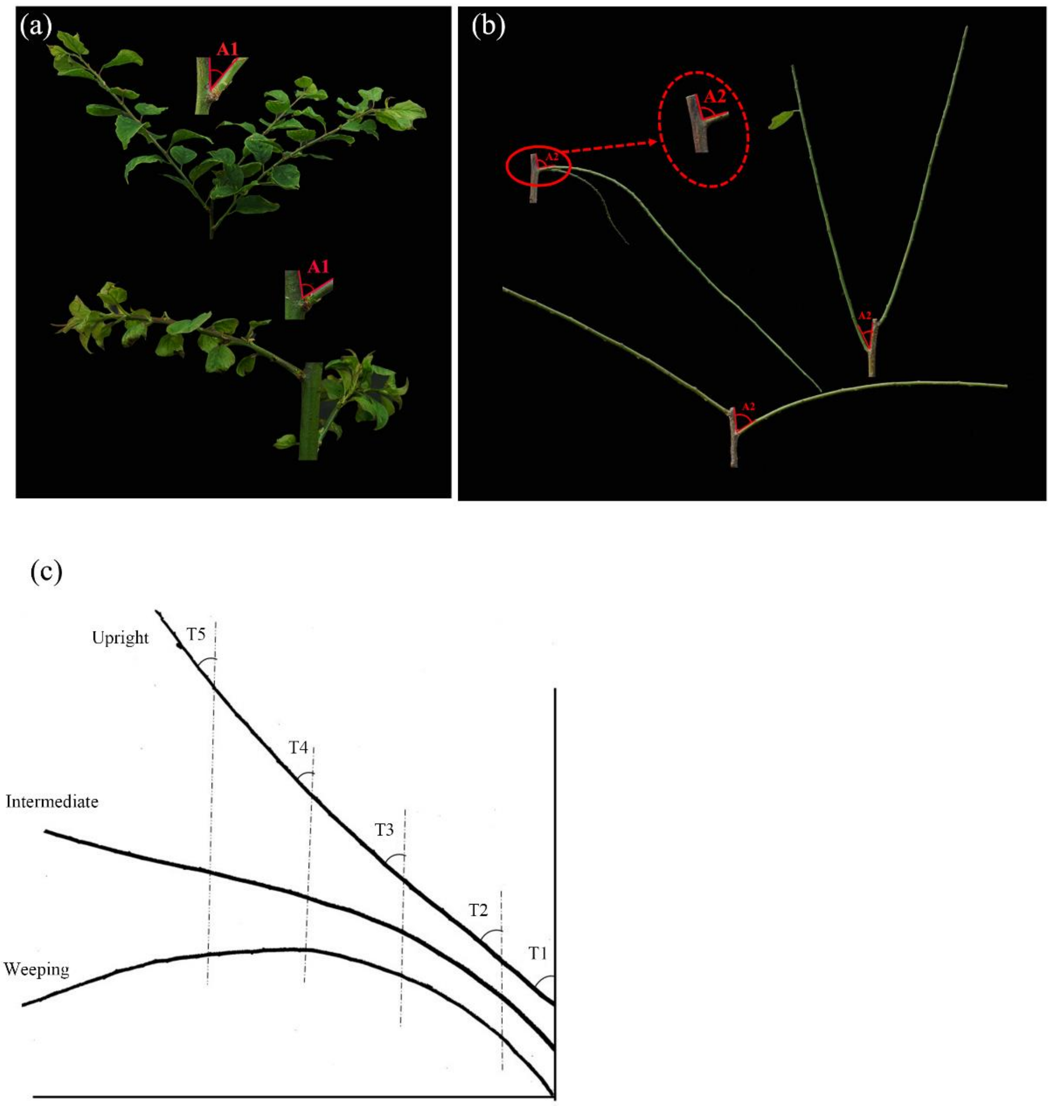

Figure S4 Distribution density of SNPs on each chromosome.

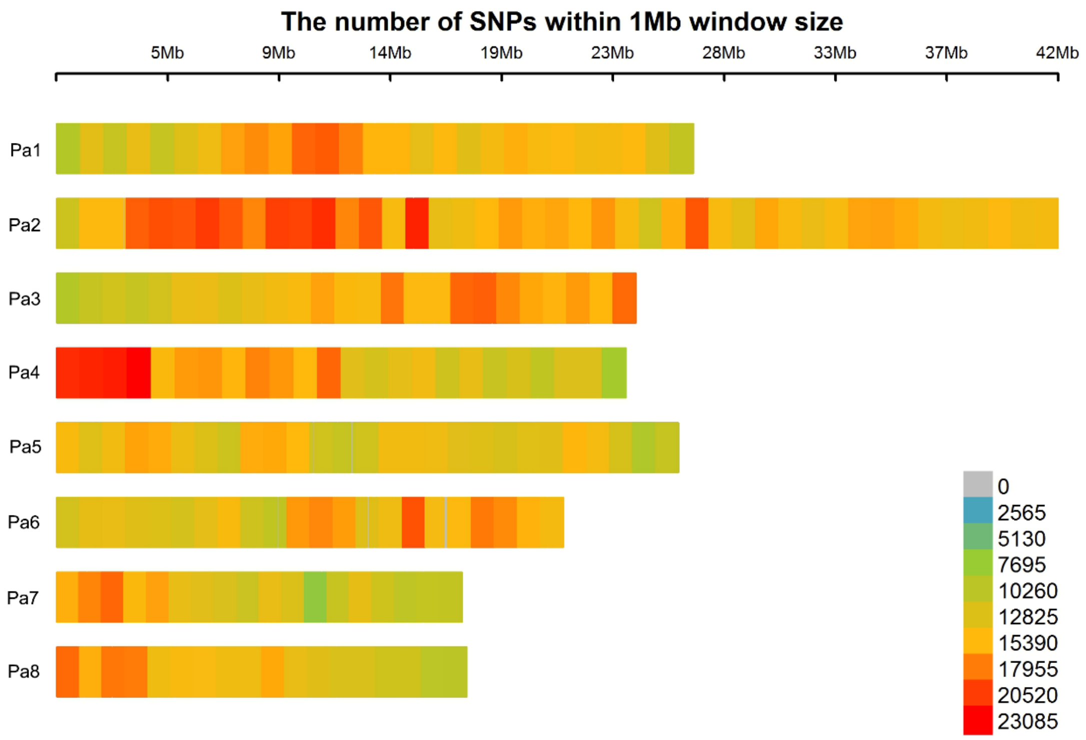

Figure S5 Estimation of the population genetic architecture for the GWAS panel. **(a)** A k=14 minimizes the cross-validation (CV) error value within the panel inferred with ADMIXTURE1.3 program. **(b)** Scree plot of former 11 variant proportion.

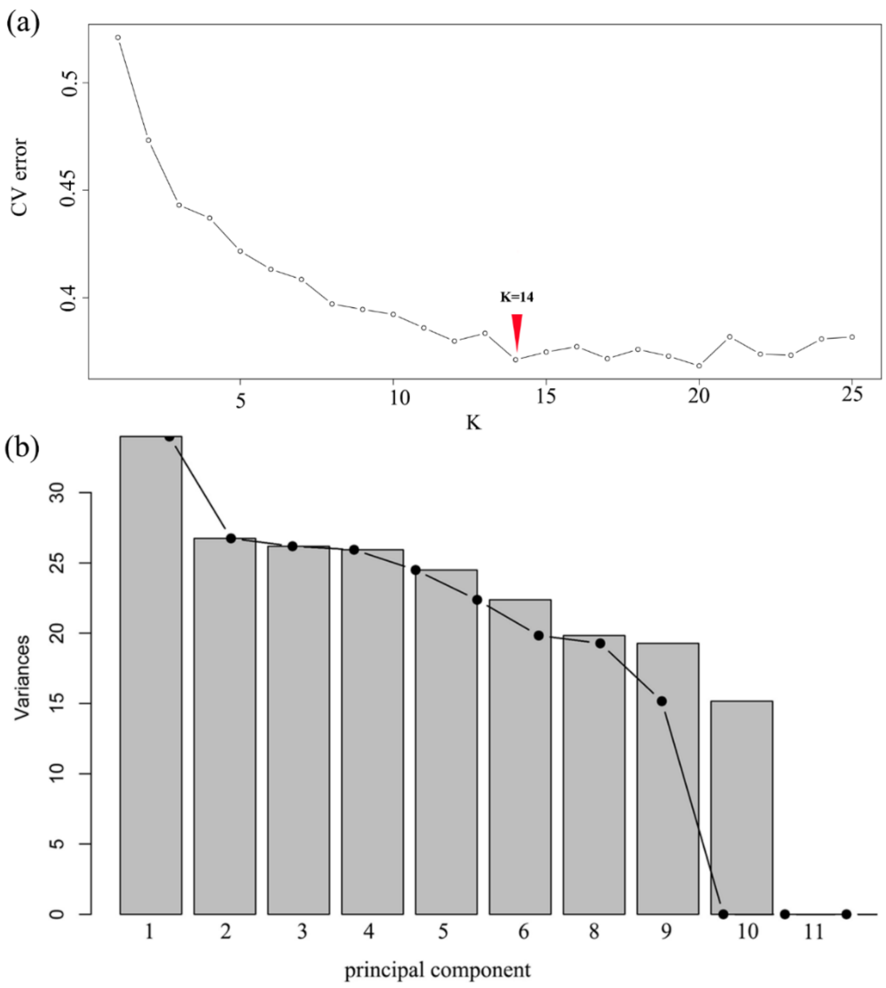

Figure S6 Correlation analysis of seven sub-traits in **(a)** the F1 population derived from 'LiuBan' × 'Fentai Chuizhi' and in **(b)** the GWAS panel.

(a)

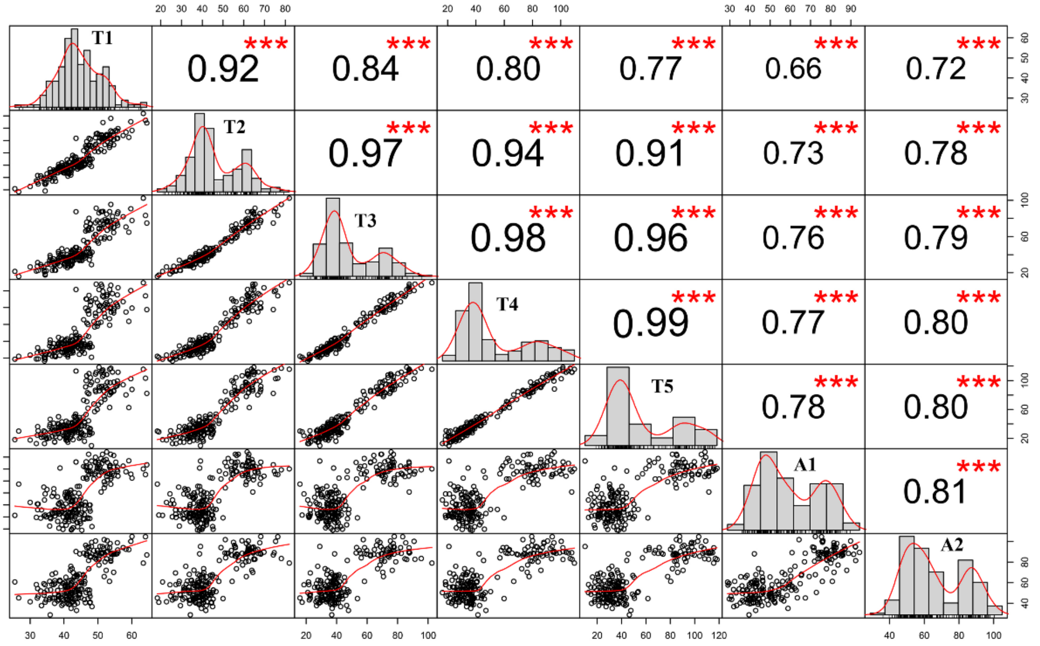

(b)

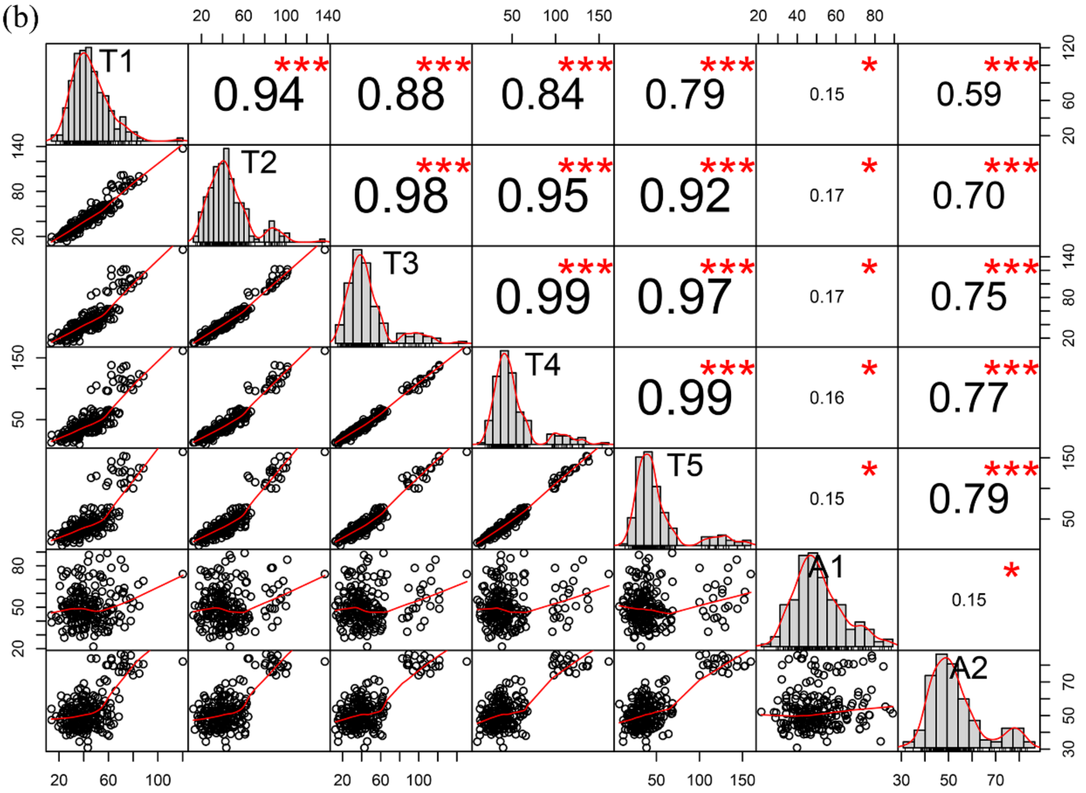

Figure S7 Frequency distribution patterns of seven sub-traits in the F1 population and GWAS panel. **(a)** 342 individuals derived from ‘LiuBan’ × ‘Fentai Chuizhi’. Red arrow indicates the phenotypes of ‘Liuban’ (LB) and ‘FenTai ChuiZhi’ (FT). **(b)** 214 landraces of *P. mume*.

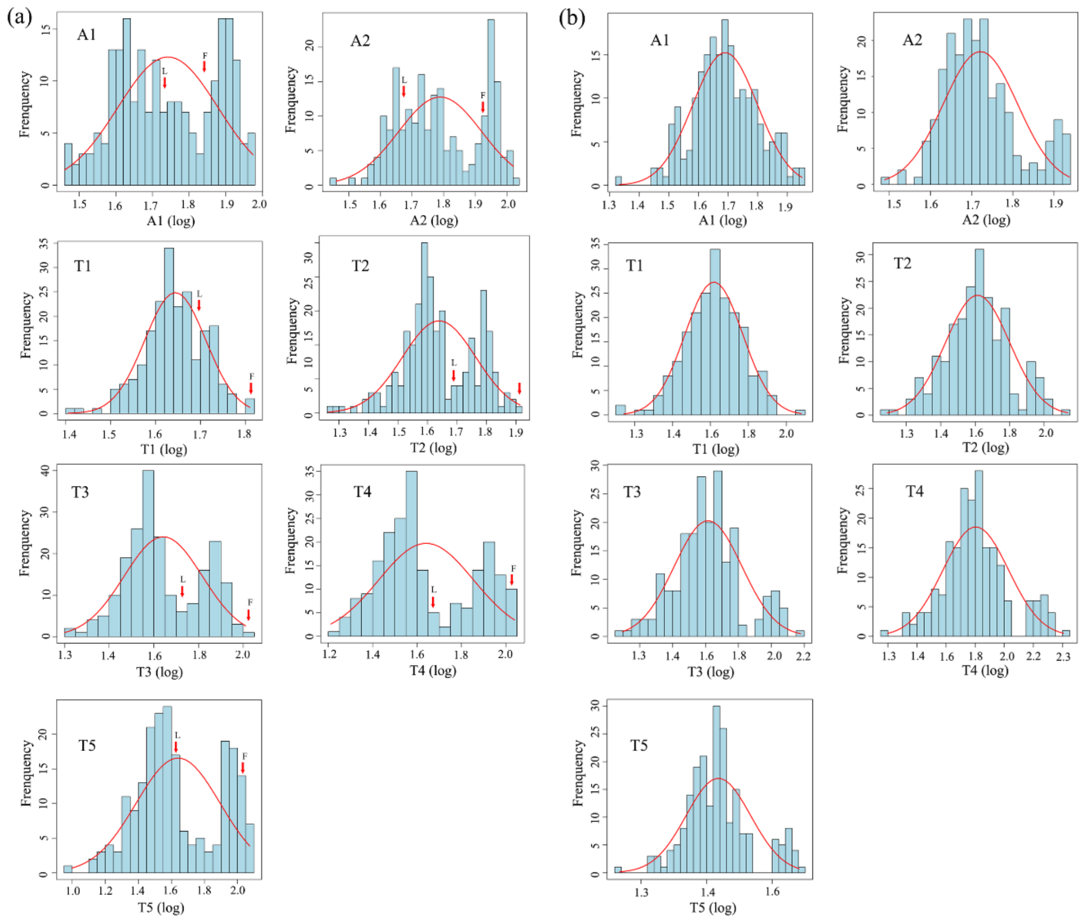

Figure S8 Selection of suitable models for the association analysis. (**a**) A multinomial logistic model (MLM) with principal components scores (PCs) and kinship matrix (K) used to correct subpopulation structure. **(b)** MLM with population structure matrix (Q) and kinship matrix (K) used to correct subpopulation structure.

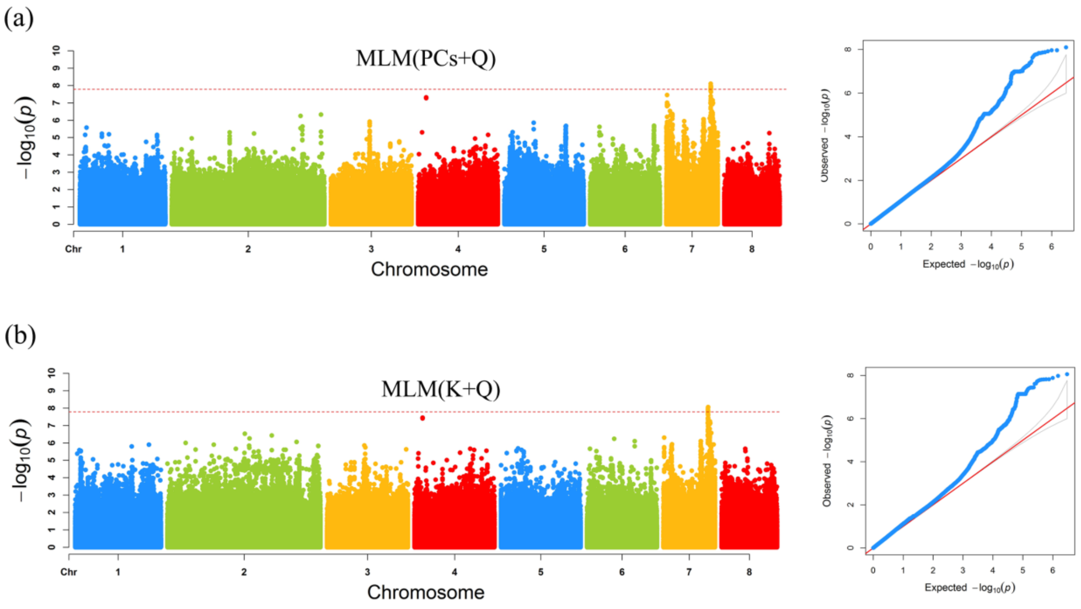

Figure S9 Validation of nineteen GWAS-derived significant SNPs in hybrids and landraces populations using the Sequenom MassARRAY analysis. 289 hybrids (126 weeping and 163 upright) and 69 landraces (25 weeping and 44 upright) were genotyped. SNPs exhibited remarkably linked to weeping trait except Pa7_15909400 allele.

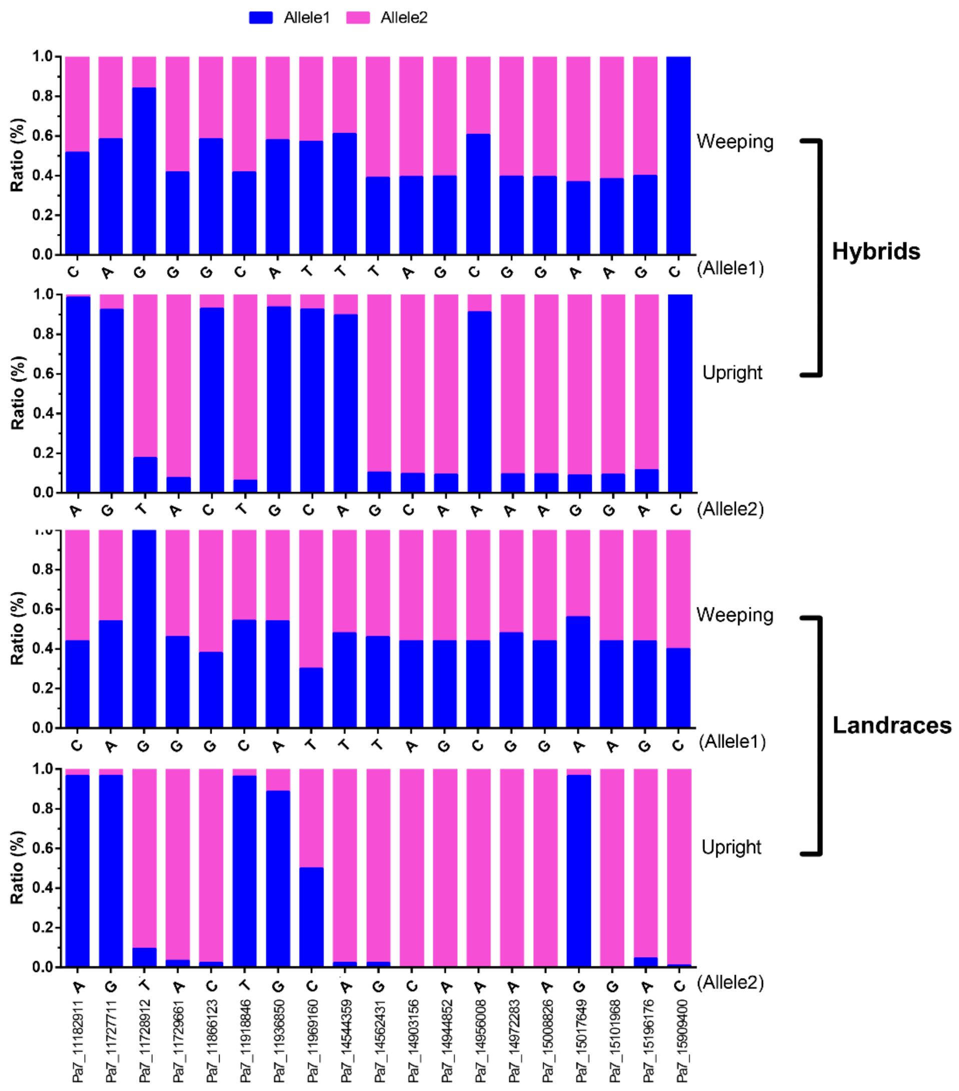

Figure S10 Validation of eight QTL-derived significant markers in 129 landraces. 30 weeping trees and 99 upright trees.

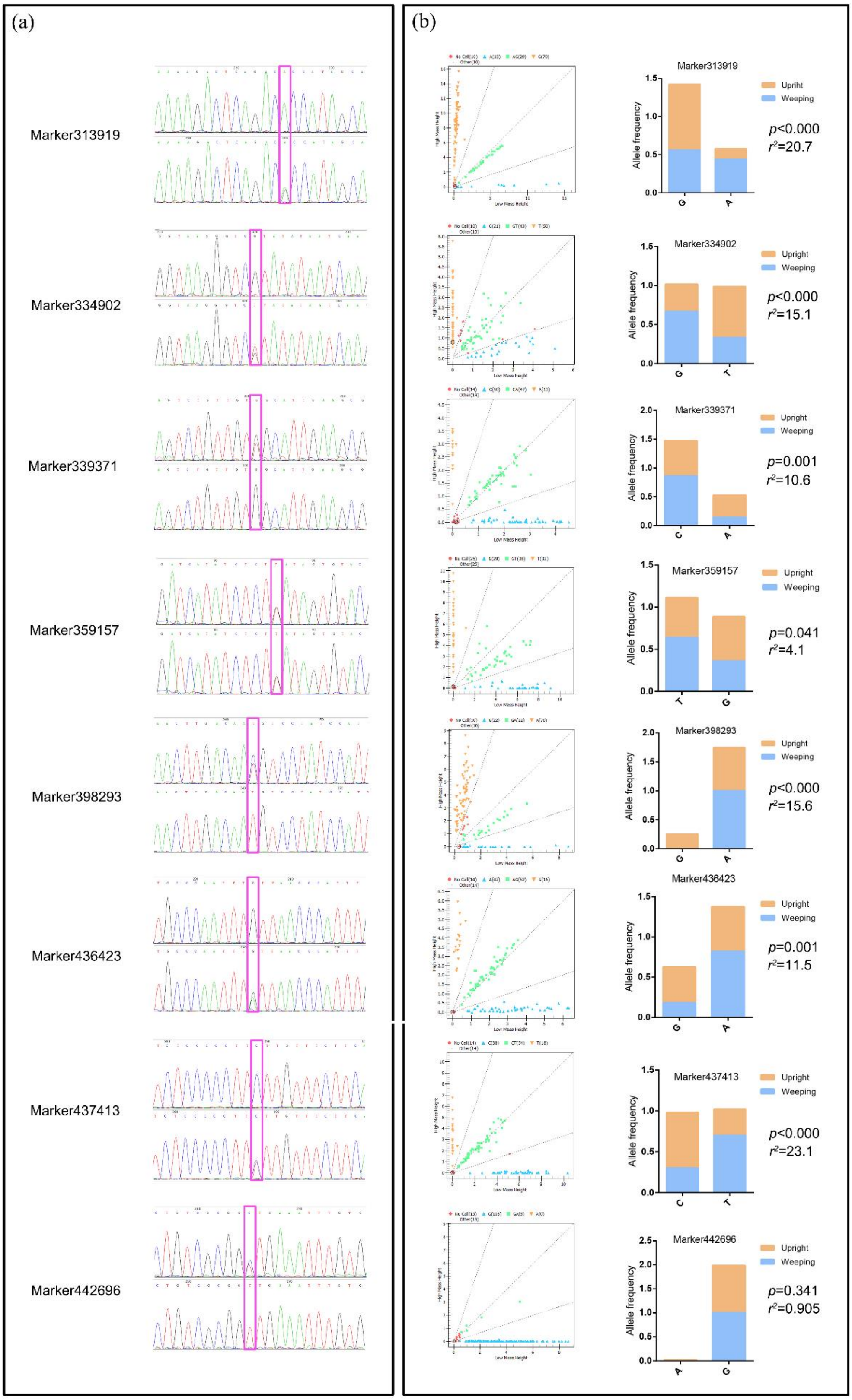

Figure S11 Prediction of Pm024213 transmembrane domains and subcellular localization. **(a)** Prediction of membrane-bound. **(b)** Subcellular localization.

(a)

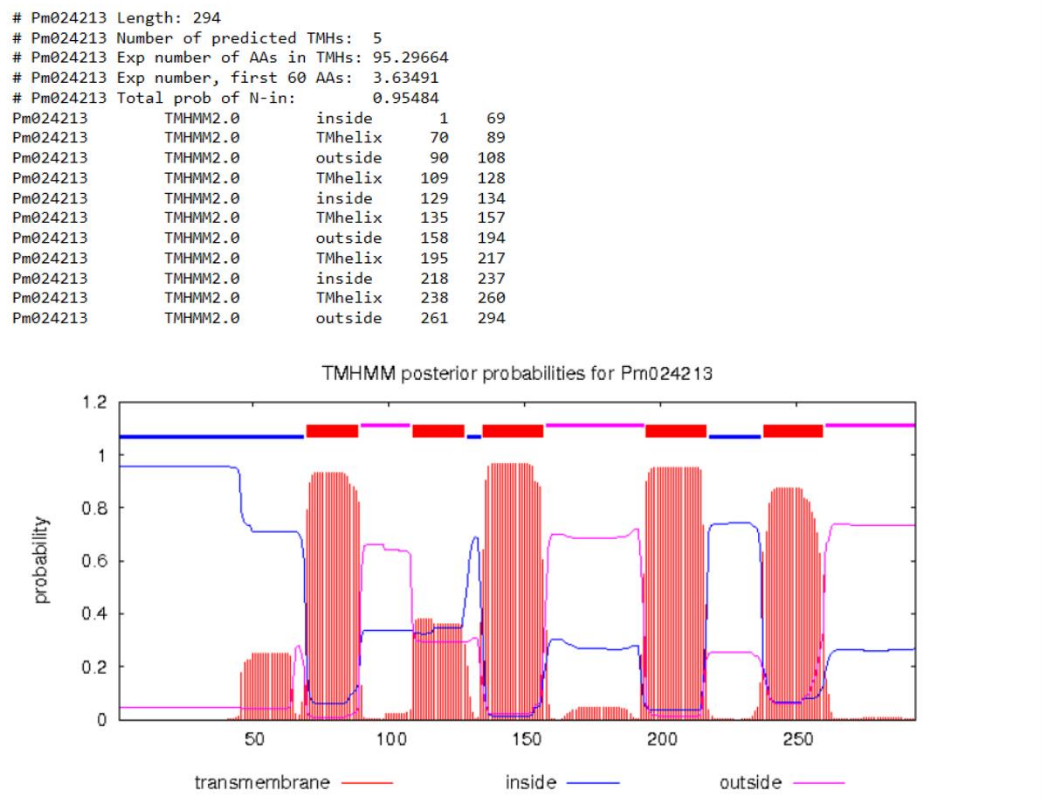

(b)

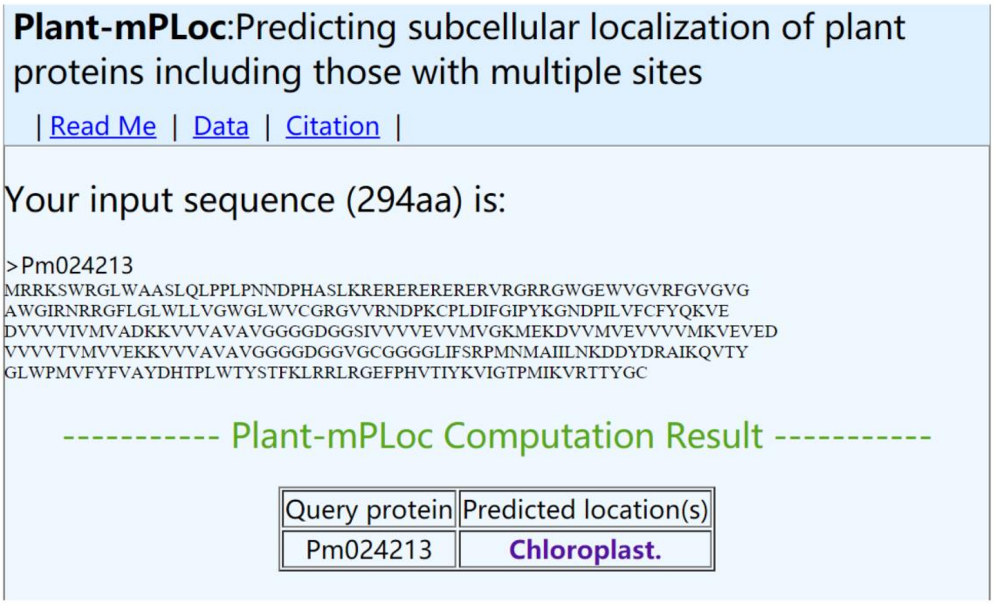

Figure S12 Neighbor-Joining phylogenetic trees of Pm024213 homologous protein from thirteen species.

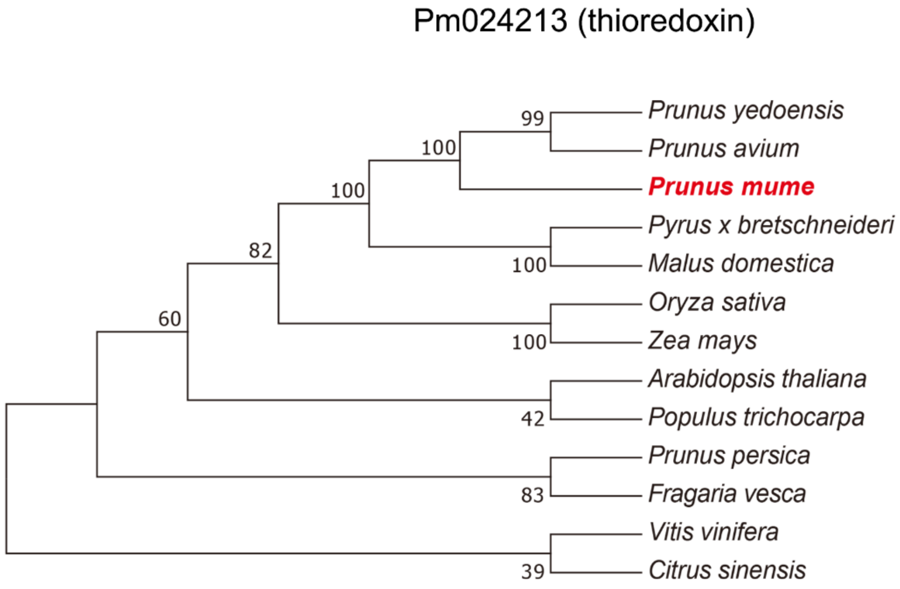

Figure S13 Co-expression analysis of *Pm024213*. **(a)** Identification of genes co-expressed with *Pm024213* genes using k-means clustering. T1-T3: bud pools of upright trees; T4-T6: branch pools of upright trees; T7-T8: bud pools of weeping trees; and T9-T12: branch pools of weeping trees. **(b)** Thirty-five of the 85 associated genes were located in epistatic loci. Red words: up-regulated genes; blue words: down-regulated genes.

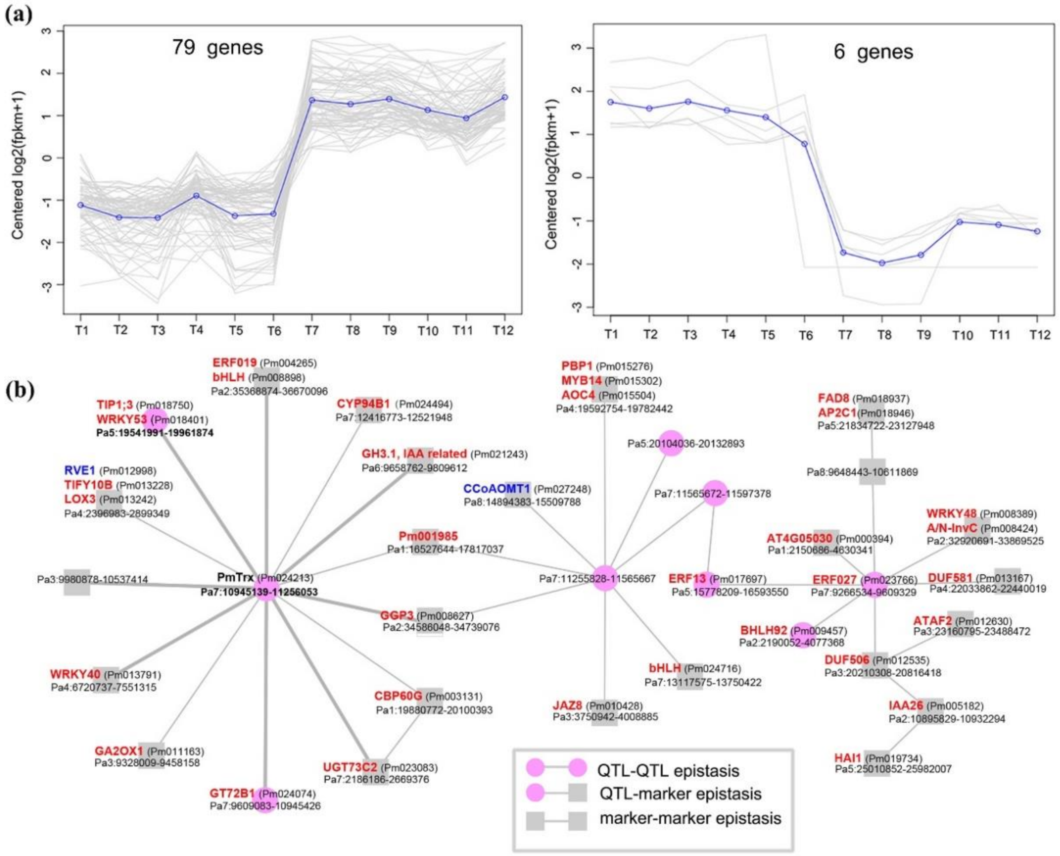

Figure S14 Functional visualization of 85 genes associated with *Pm024213* identified using the co-expression analysis.

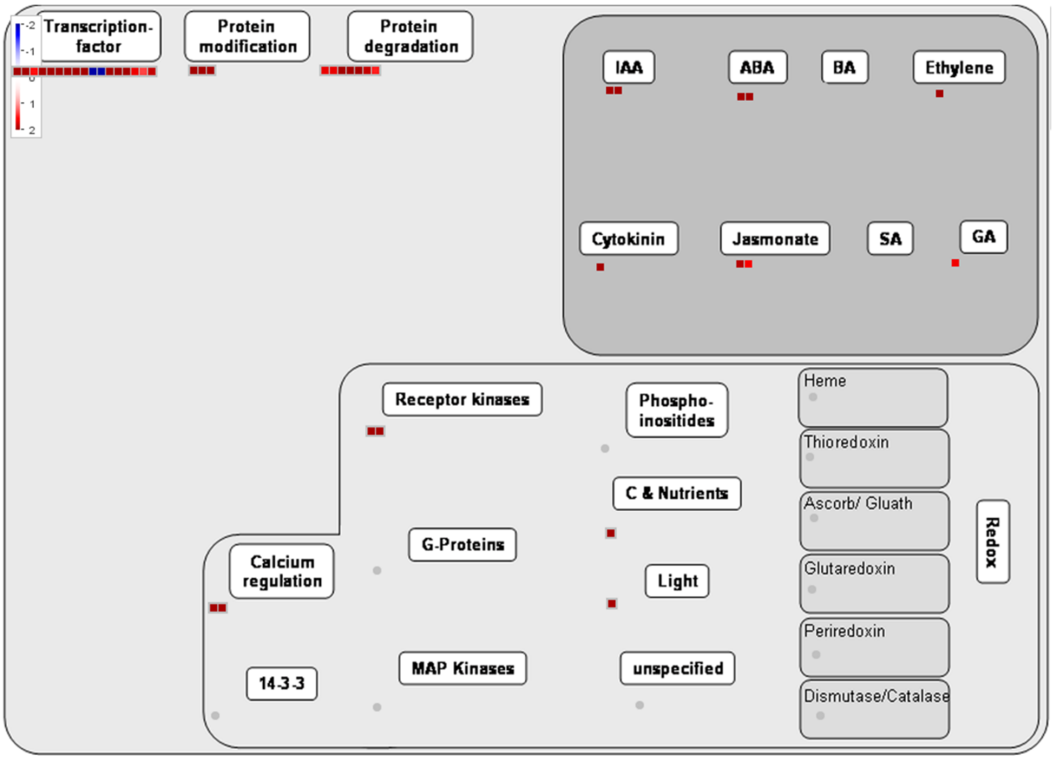

Supplement: Supplementary file 5 — Supporting figure [file 41438_2021_573_MOESM5_ESM.pdf]
